# Healthy dietary patterns, longevity genes, and life expectancy: A prospective cohort study

**DOI:** 10.1126/sciadv.ads7559

**Published:** 2026-02-13

**Authors:** Yanling Lv, Jing Song, Ding Ding, Mengyun Luo, Feng J. He, Changzheng Yuan, Graham A. MacGregor, Liegang Liu, Liangkai Chen

**Affiliations:** ^1^ Department of Nutrition and Food Hygiene, Hubei Key Laboratory of Food Nutrition and Safety, School of Public Health, Tongji Medical College, Huazhong University of Science and Technology, Wuhan 430030, China.; ^2^ Ministry of Education Key Lab of Environment and Health, School of Public Health, Tongji Medical College, Huazhong University of Science and Technology, Wuhan 430030, China.; ^3^ Wolfson Institute of Population Health, Barts and The London School of Medicine and Dentistry, Queen Mary University of London, London, UK.; ^4^ Department of Health Services Research and Policy, Faculty of Public Health and Policy, London School of Hygiene and Tropical Medicine, London WC1H 9SH, UK.; ^5^ Prevention Research Collaboration, Sydney School of Public Health, The University of Sydney, New South Wales, Australia.; ^6^ Charles Perkins Centre, The University of Sydney, New South Wales, Australia.; ^7^ School of Public Health, the Second Affiliated Hospital, Zhejiang University School of Medicine, Hangzhou, China.; ^8^ Department of Nutrition, Harvard T. H. Chan School of Public Health, Boston, MA, USA.

## Abstract

Associations between healthy dietary patterns and life expectancy remain unclear. Here, we reported the prospective associations of five dietary patterns with mortality and life expectancy in 103,649 UK Biobank participants. Over a median follow-up period of 10.6 years, 4314 total deaths were documented. Alternate Healthy Eating Index-2010, Alternate Mediterranean Diet (AMED), healthful Plant-based Diet Index (hPDI), Dietary Approaches to Stop Hypertension, and Diabetes Risk Reduction Diet (DRRD) were associated with lower all-cause mortality and longer life expectancy, with DRRD showing slightly stronger associations than hPDI. Compared with the bottom quintile, achieving the top quintile of dietary scores was associated with 1.9 to 3.0 years of life gained at 45 years in men and 1.5 to 2.3 years in women. The life gained was longest in DRRD for males and AMED for females. The significant associations remained when accounting for genetic susceptibility. Our findings underscore the advantages of healthy dietary patterns in prolonging life expectancy, regardless of longevity genes.

## Teaser

Healthy dietary patterns are linked to longer life expectancy and can be adapted to personal preferences and cultural traditions.

## INTRODUCTION

Following decades of rapid increase, the trajectory of life expectancy has decelerated in recent years (*1*). As highlighted by the 2017 Global Burden of Disease Study, an unhealthy dietary regimen stands out as a primary cause of death globally (*2*), underscoring the adoption of a healthy diet might constitute a potentially cost-effective strategy for mitigating premature death and augmenting life expectancy (*3*). Dietary patterns, which consist of a variety of food groups and account for their potentially synergistic or antagonistic effects, are an essential approach and are increasingly used to estimate the association between diet and mortality (*4*). Noteworthy, a priori dietary pattern scores measuring the adherence to a dietary pattern defined on the basis of previous scientific evidence, such as the Alternate Healthy Eating Index (AHEI), the Alternate Mediterranean Diet score (AMED), the healthful Plant-based Diet Index (hPDI), the Dietary Approaches to Stop Hypertension (DASH), and the Diabetes Risk Reduction Diet (DRRD), are associated with reduced risks of chronic diseases and mortality (*5*–*7*). Nonetheless, the full extent of the associations between these healthy dietary patterns and life expectancy remains incompletely understood; only a few studies have reported the association between healthy dietary patterns and life expectancy using real-world data (*8*, *9*). Given that life expectancy embodies an absolute metric, estimating the “life gain” attributed to a healthier dietary pattern compared to an unhealthy one is more interpretable for the public and policy-makers than relative metrics, such as hazard ratios (HRs) (*10*).

Evidence has shown the effect of genetic determinants on longevity, with increasing longevity-associated mutations being discovered as human genetic research progresses (*11*, *12*). In addition, the genetic architecture plays an important role in nutrient intake and metabolism (*13*, *14*). Recently, a growing body of studies has shown the interaction between genetic risk and environmental risk factors like diet (*15*, *16*). However, the interplay between dietary quality, genetic predisposition toward shortened life span, and their combined effects on life expectancy remains largely unknown. Therefore, this study aimed to assess the associations of healthy dietary patterns with mortality and life expectancy in the UK Biobank. In addition, we aimed to evaluate the interaction and joint association between dietary patterns and genetic susceptibility to short life span on all-cause mortality and life expectancy.

## RESULTS

### Participant characteristics

The final study sample comprised 103,649 participants [mean (SD) age, 58.3 (7.8) years; 56.4% female] with two or more dietary assessments and without prior cardiovascular diseases (CVDs) or cancer. Table 1 shows the characteristics of study participants by quintiles of dietary scores. Participants with higher dietary scores tended to be older, better educated, less deprived, less likely to smoke, more physically active, consumed less alcohol, and had a lower body mass index (BMI). The distribution of each dietary score was generally normal (fig. S1) and exhibited moderate to high intercorrelations, with Spearman’s correlation coefficients ranging from 0.44 to 0.88 (fig. S2). Correlations between food groups and the five dietary scores are shown in fig. S3.

**Table 1.** Baseline characteristics by quintiles of dietary scores. IQR, interquartile range; TDI, Townsend Deprivation Index.

Characteristics	Overall	AHEI		AMED		hPDI		DASH		DRRD	
	(*n* = 103,649)	Quintile 1 (*n* = 20,729)	Quintile 5 (*n* = 20,730)	Quintile 1 (*n* = 21,530)	Quintile 5 (*n* = 19,839)	Quintile 1 (*n* = 18,984)	Quintile 5 (*n* = 20,010)	Quintile 1 (*n* = 22,185)	Quintile 5 (*n* = 19,875)	Quintile 1 (*n* = 18,488)	Quintile 5 (*n* = 19,554)
Age, mean (SD), *y*	58.3 (7.8)	56.6 (8.0)	59.5 (7.5)	57.0 (7.9)	59.4 (7.6)	56.6 (8.2)	59.2 (7.4)	56.3 (8.0)	59.6 (7.4)	57.2 (7.9)	59.2 (7.6)
Male, *n* (%)	45,171 (43.6)	12,516 (60.4)	5,956 (28.7)	7,877 (36.6)	9,645 (48.6)	11,071 (58.3)	6,097 (30.5)	13,195 (59.5)	5,739 (28.9)	9,388 (50.8)	7,176 (36.7)
White, *n* (%)	99,910 (96.4)	20,084 (96.9)	19,832 (95.7)	20,742 (96.3)	19,112 (96.3)	18,268 (96.2)	19,239 (96.2)	21,429 (96.6)	19,063 (95.9)	17,858 (96.6)	18,656 (95.4)
TDI, median (IQR)	−2.3 (−3.8, 0)	−2.2 (−3.7, 0.3)	−2.3 (−3.7, 0.1)	−2.2 (−3.7, 0.3)	−2.3 (−3.8, 0)	−2.3 (−3.7, 0.1)	−2.3 (−3.7, 0.1)	−2.2 (−3.7, 0.2)	−2.3 (−3.7, 0.1)	−2.3 (−3.7, 0.1)	−2.3 (−3.7, 0.2)
**Deprivation quintiles, *n* (%)**											
1 (least deprived)	20,694 (20.0)	3,964 (19.1)	4,079 (19.7)	4,015 (18.7)	3,969 (20.0)	3,679 (19.4)	3,845 (19.2)	4,298 (19.4)	3,850 (19.4)	3,622 (19.6)	3,798 (19.4)
2–4	62,127 (59.9)	12,237 (59.0)	12,373 (59.7)	12,768 (59.3)	11,858 (59.8)	11,292 (59.5)	11,969 (59.8)	13,114 (59.1)	11,877 (59.8)	10,971 (59.3)	11,574 (59.2)
5 (most deprived)	20,704 (20.0)	4,492 (21.7)	4,260 (20.6)	4,720 (21.9)	3,995 (20.1)	3,975 (20.9)	4,179 (20.9)	4,734 (21.3)	4,127 (20.8)	3,866 (20.9)	4,164 (21.3)
Unknown	124 (0.1)	36 (0.2)	18 (0.1)	27 (0.1)	17 (0.1)	38 (0.2)	17 (0.1)	39 (0.2)	21 (0.1)	29 (0.2)	18 (0.1)
Education											
College or university	49,698 (48.0)	9,074 (43.8)	10,970 (52.9)	8,299 (38.6)	11,270 (56.8)	8,166 (43.0)	10,830 (54.1)	9,458 (42.6)	10,455 (52.6)	7,744 (41.9)	10,477 (53.6)
Vocational	9,770 (9.4)	1,981 (9.6)	1,888 (9.1)	2,099 (9.8)	1,722 (8.7)	1,855 (9.8)	1,813 (9.1)	2,169 (9.8)	1,813 (9.1)	1,805 (9.8)	1,696 (8.7)
Upper secondary	14,076 (13.6)	2,983 (14.4)	2,701 (13.0)	3,142 (14.6)	2,471 (12.5)	2,749 (14.5)	2,574 (12.9)	3,228 (14.6)	2,540 (12.8)	2,644 (14.3)	2,546 (13)
Lower secondary	23,800 (23.0)	5,428 (26.2)	4,062 (19.6)	6,312 (29.3)	3,501 (17.7)	5,019 (26.4)	3,796 (19.0)	5,934 (26.8)	3,955 (19.9)	4,982 (27.0)	3,822 (19.6)
Others	6,000 (5.8)	1,207 (5.8)	1,053 (5.1)	1,609 (7.5)	843 (4.3)	1,136 (6.0)	944 (4.7)	1,328 (6.0)	1,063 (5.4)	1,249 (6.8)	974 (5.0)
Unknown	305 (0.3)	56 (0.3)	56 (0.3)	69 (0.3)	32 (0.2)	59 (0.3)	53 (0.3)	68 (0.3)	49 (0.3)	64 (0.4)	39 (0.2)
Alcohol consumption, median (IQR), g/day	11.2 (0, 26.0)	23.1 (0, 42.9)	8.1 (0, 17.1)	15.4 (0, 35.2)	10.2 (4.5, 18.7)	12.2 (0, 29.1)	9.0 (0, 21.7)	14.5 (0.1, 32.5)	7.7 (0, 19.4)	13.2 (0.1, 30.9)	8.6 (0, 21.4)
Current smoker, *n* (%)	7,139 (6.9)	2,329 (11.2)	864 (4.2)	2,268 (10.5)	884 (4.5)	1,659 (8.7)	1,032 (5.2)	2,366 (10.7)	849 (4.3)	1,779 (9.6)	983 (5.0)
BMI, mean (SD), kg/m^2^	26.6 (4.5)	27.8 (4.8)	25.4 (4.1)	27.4 (4.9)	25.7 (4.1)	27.8 (5.0)	25.6 (4.1)	27.7 (4.8)	25.5 (4.2)	27.5 (4.9)	25.7 (4.2)
Energy intake, mean (SD), kcal/day	2,044.2 (467.7)	2,210.7 (484.0)	1,944.3 (434.5)	1,949.4 (469.4)	2,161.2 (466.1)	2,299.5 (469.0)	1,851.8 (423.5)	2,180.2 (480.2)	1,947.8 (433.7)	2,019.6 (464.0)	2,065.9 (466.3)
**Total physical activity, MET-min/week**											
0–599	16,078 (15.5)	4,042 (19.5)	2,333 (11.3)	4,277 (19.9)	2,167 (10.9)	3,734 (19.7)	2,369 (11.8)	4,546 (20.5)	2,187 (11.0)	3,714 (20.1)	2,231 (11.4)
600–1199	16,773 (16.2)	3,465 (16.7)	3,173 (15.3)	3,594 (16.7)	3,000 (15.1)	3,174 (16.7)	3,061 (15.3)	3,764 (17.0)	2,977 (15.0)	3,058 (16.5)	2,952 (15.1)
≥1200	56,313 (54.3)	10,423 (50.3)	12,405 (59.8)	10,249 (47.6)	12,306 (62.0)	9,472 (49.9)	11,934 (59.6)	10,848 (48.9)	12,048 (60.6)	8,986 (48.6)	11,806 (60.4)
Unknown	14,485 (14.0)	2,799 (13.5)	2,819 (13.6)	3,410 (15.8)	2,366 (11.9)	2,604 (13.7)	2,646 (13.2)	3,027 (13.6)	2,663 (13.4)	2,730 (14.8)	2,565 (13.1)
Dyslipidemia, *n* (%)	47,412 (45.7)	10,862 (52.4)	8,125 (39.2)	10,275 (47.7)	8,502 (42.9)	9,919 (52.3)	7,857 (39.3)	11,617 (52.4)	7,831 (39.4)	9,263 (50.1)	8,111 (41.5)
Hypertension, *n* (%)	51,000 (49.2)	10,972 (52.9)	9,471 (45.7)	10,578 (49.1)	9,657 (48.7)	9,811 (51.7)	9,147 (45.7)	11,372 (51.3)	9,314 (46.9)	9,529 (51.5)	9,185 (47.0)
Diabetes, *n* (%)	4,175 (4.0)	1,019 (4.9)	690 (3.3)	932 (4.3)	760 (3.8)	935 (4.9)	682 (3.4)	1,052 (4.7)	693 (3.5)	828 (4.5)	753 (3.9)
PRS, mean (SD)	17.6 (2.3)	17.5 (2.3)	17.5 (2.3)	17.5 (2.3)	17.6 (2.3)	17.6 (2.3)	17.5 (2.3)	17.6 (2.3)	17.5 (2.3)	17.6 (2.3)	17.5 (2.3)

### Associations of dietary patterns with all-cause and cause-specific mortality

**Fig. 1.**
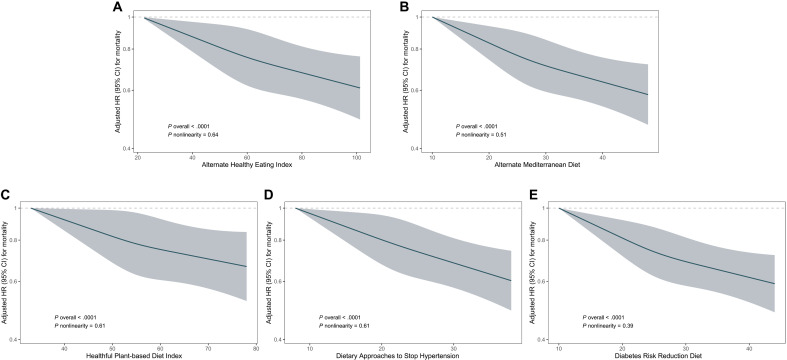
The restricted cubic splines for associations between dietary patterns and the risk of all-cause mortality. (**A**) AHEI. (**B**) AMED. (**C**) hPDI. (**D**) DASH. (**E**) DRRD. The *y* axis is plotted on a log scale. Adjusted for age (in years, continuous), sex (male/female), ethnicity (white/not-white), education (lower secondary, upper secondary, vocational, college or university, or others), TDI (in quintiles), assessment centers (22 categories), smoking (current, former, or never), physical activity (0 to 599, 600 to 1199, or ≥1200 MET-min/week, or unknown), BMI (<25.0, 25.0 to 29.9, or ≥30 kg/m^2^, or unknown), total energy intake (kilocalories, continuous), baseline dyslipidemia (yes/no), hypertension (yes/no), diabetes (yes/no), longevity polygenic risk score (PRS) (in tertiles), top 10 genetic primary components (continuous), and genotype measurement batches (continuous); alcohol consumption (0, 0.1 to 5, 5.1 to 10, 10.1 to 15, 15.1 to 20, 20.1 to 30, or >30 g/day) was adjusted for hPDI, DASH, and DRRD. MET, metabolic equivalent of task.

During a median follow-up of 10.6 years (1,094,467 person-years), 4314 total deaths were documented. After adjusting for potential confounders, all dietary scores were linearly associated with a reduced risk of all-cause mortality (Fig. 1). Comparing the highest quintile to the lowest, multivariable-adjusted HRs with 95% confidence intervals (CIs) were 0.80 (0.73, 0.89) for AHEI, 0.80 (0.72, 0.88) for AMED, 0.82 (0.74, 0.92) for hPDI, 0.81 (0.73, 0.90) for DASH, and 0.76 (0.69, 0.84) for DRRD (all *P* for trend < 0.0001; Table 2). When differentiating CVD, cancer, neurodegenerative disease, respiratory disease, and other-cause mortality from all deaths, higher dietary scores tended to be associated with lower risk of cause-specific mortalities (fig. S4), and a significant inverse association was observed in cancer mortality, respiratory disease mortality, and other-cause mortality in the final model (tables S1 to S5).

**Table 2.** HR (95% CI) of death from all causes according to quintiles of AHEI, AMED, hPDI, DASH, and DRRD. Model 1 adjusted for age (in years, continuous) and sex (male/female). Model 2 adjusted for covariates in model 1 plus ethnicity (white/not-white), education (lower secondary, upper secondary, vocational, college or university, or others), TDI (in quintiles), assessment centers (22 categories), smoking (current, former, or never), physical activity (0 to 599, 600 to 1199, or ≥1200 MET-min/week, or unknown), BMI (<25.0, 25.0 to 29.9, or ≥30 kg/m^2^, or unknown), total energy intake (kilocalories, continuous), baseline dyslipidemia (yes/no), hypertension (yes/no), diabetes (yes/no), longevity PRS (in tertiles), top 10 genetic primary components (continuous), and genotype measurement batches (continuous); alcohol consumption (0, 0.1 to 5, 5.1 to 10, 10.1 to 15, 15.1 to 20, 20.1 to 30, or >30 g/day) was adjusted for hPDI, DASH, and DRRD.

	Quintile of dietary score					*P* for trend	Per SD increment
	Quintile 1	Quintile 2	Quintile 3	Quintile 4	Quintile 5		
**AHEI**							
Median score	45 (41, 48)	54 (52, 56)	61 (59, 62)	67 (65, 69)	76 (73, 81)		
Cases/person-years	996/218,823	898/218,711	876/218,791	801/219,021	743/219,122		
Model 1	1.00 (reference)	0.83 (0.76, 0.91)	0.78 (0.72, 0.86)	0.71 (0.65, 0.78)	0.66 (0.60, 0.73)	<0.0001	0.86 (0.84, 0.89)
Model 2	1.00 (reference)	0.90 (0.83, 0.99)	0.88 (0.81, 0.97)	0.83 (0.75, 0.91)	0.80 (0.73, 0.89)	<0.0001	0.93 (0.90, 0.96)
**AMED**							
Median score	20 (18, 21)	24 (23, 25)	27 (26, 28)	30 (29, 31)	35 (34, 37)		
Cases/person-years	942/227,792	879/209,644	858/218,227	869/229,324	766/209,481		
Model 1	1.00 (reference)	0.90 (0.82, 0.98)	0.79 (0.72, 0.87)	0.73 (0.67, 0.80)	0.67 (0.61, 0.74)	<0.0001	0.86 (0.84, 0.89)
Model 2	1.00 (reference)	0.95 (0.87, 1.05)	0.88 (0.81, 0.97)	0.84 (0.76, 0.92)	0.80 (0.72, 0.88)	<0.0001	0.92 (0.89, 0.95)
**hPDI**							
Median score	47 (45, 48)	52 (51, 53)	55 (54, 56)	58 (57, 59)	63 (62, 65)		
Cases/person-years	864/200,289	1014/236,866	852/209,446	872/236,034	712/211,832		
Model 1	1.00 (reference)	0.91 (0.83, 0.998)	0.84 (0.77, 0.93)	0.77 (0.70, 0.85)	0.72 (0.65, 0.80)	<0.0001	0.90 (0.87, 0.93)
Model 2	1.00 (reference)	0.97 (0.88, 1.06)	0.92 (0.84, 1.02)	0.86 (0.78, 0.95)	0.82 (0.74, 0.92)	<0.0001	0.95 (0.91, 0.98)
**DASH**							
Median score	16 (15, 18)	20 (19, 21)	22 (22, 23)	25 (24, 26)	29 (27, 30)		
Cases/person-years	993/234,222	1049/241,870	673/179,063	875/229,151	724/210,162		
Model 1	1.00 (reference)	0.91 (0.83, 0.99)	0.77 (0.69, 0.85)	0.76 (0.70, 0.84)	0.69 (0.63, 0.76)	<0.0001	0.87 (0.84, 0.90)
Model 2	1.00 (reference)	0.99 (0.90, 1.08)	0.86 (0.78, 0.95)	0.87 (0.79, 0.96)	0.81 (0.73, 0.90)	<0.0001	0.92 (0.89, 0.95)
**DRRD**							
Median score	18 (17, 20)	23 (22, 24)	25 (25, 26)	28 (27, 29)	33 (32, 35)		
Cases/person-years	876/194,850	1117/278,220	628/154,510	990/259,930	703/206,957		
Model 1	1.00 (reference)	0.84 (0.76, 0.91)	0.83 (0.75, 0.92)	0.78 (0.71, 0.85)	0.68 (0.62, 0.75)	<0.0001	0.88 (0.86, 0.91)
Model 2	1.00 (reference)	0.89 (0.81, 0.97)	0.89 (0.80, 0.99)	0.85 (0.78, 0.93)	0.76 (0.69, 0.84)	<0.0001	0.92 (0.89, 0.95)

### Joint analysis of dietary patterns and longevity genes with all-cause mortality

**Fig. 2.**
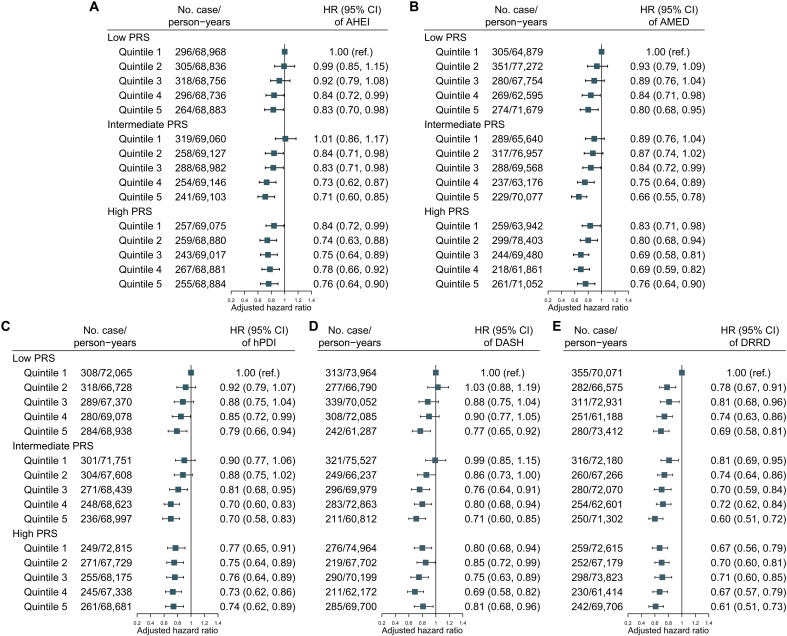
Joint association of dietary patterns and longevity genes with all-cause mortality. (**A**) AHEI. (**B**) AMED. (**C**) hPDI. (**D**) DASH. (**E**) DRRD. Adjusted for age (in years, continuous), sex (male/female), ethnicity (white/not-white), education (lower secondary, upper secondary, vocational, college or university, or others), TDI (in quintiles), assessment centers (22 categories), smoking (current, former, or never), physical activity (0 to 599, 600 to 1199, or ≥1200 MET-min/week, or unknown), BMI (<25.0, 25.0 to 29.9, or ≥30 kg/m^2^, or unknown), total energy intake (kilocalories, continuous), baseline dyslipidemia (yes/no), hypertension (yes/no), diabetes (yes/no), top 10 genetic primary components (continuous), and genotype measurement batches (continuous); alcohol consumption (0, 0.1 to 5, 5.1 to 10, 10.1 to 15, 15.1 to 20, 20.1 to 30, or >30 g/day) was adjusted for hPDI, DASH, and DRRD.

The baseline characteristics of participants included in the genetic analyses were generally similar to the main analysis sample (table S6). The distribution of longevity polygenic risk score (PRS) is shown in fig. S5, presenting a linear decrease in the risk of all-cause mortality with rising genetic predisposition to longevity (fig. S6). Compared to low PRS, participants with high PRS had a 15% lower risk of total death [HR, 0.85 (95% CI: 0.78, 0.91); table S7]. When examining the dietary scores and genetic predisposition jointly, the all-cause mortality tended to decrease with higher dietary scores and higher PRS (Fig. 2). Stratification by PRS categories revealed persistent inverse associations between dietary scores and all-cause mortality, with no significant multiplicative or additive interactions observed (all *P* for interaction > 0.05; table S8), except that the association between DRRD and all-cause mortality was stronger among participants with lower longevity PRS (*P* for interaction = 0.037).

### Dietary patterns, longevity genes, and their joint associations with life expectancy

**Fig. 3.**
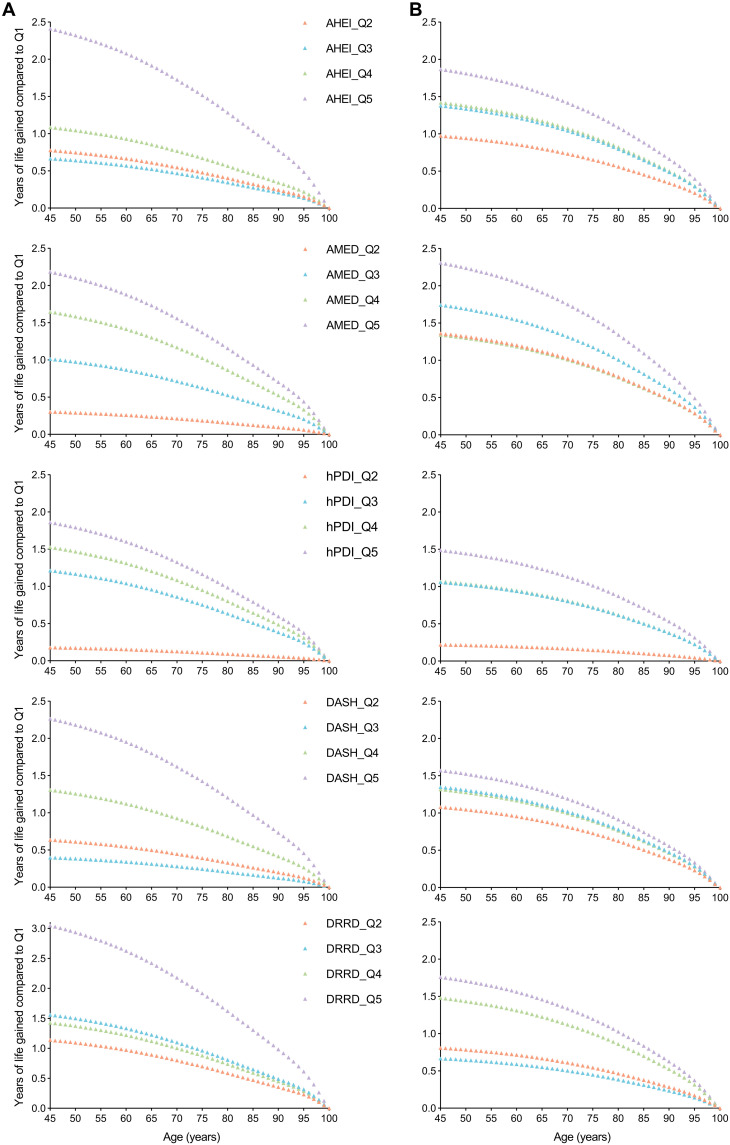
Gained years of life from 45 years of age by quintiles of dietary scores. (**A**) Gained years of life in men; (**B**) Gained years of life in women. The multivariable-adjusted HRs were used for life expectancy calculation. Q, quintile.

The estimated life expectancy at age 45 years (95% CI) was 34.0 (33.6, 34.8), 34.0 (33.2, 34.7), 34.0 (33.6, 34.9), 34.0 (33.6, 34.8), and 33.6 (33.1, 34.5) for men in the bottom quintile and 36.4 (35.6, 37.7), 36.2 (35.6, 37.1), 35.9 (35.3, 37.3), 36.3 (35.6, 37.7), and 36.7 (36.0, 38.0) for men in the top quintile of AHEI, AMED, hPDI, DASH, and DRRD, respectively. For women, the corresponding estimates were 37.3 (36.9, 37.9), 37.1 (36.8, 38.2), 37.7 (36.6, 38.6), 37.4 (36.0, 37.9), and 37.5 (36.4, 38.2) in the bottom quintile and 39.1 (38.3, 39.6), 39.4 (38.3, 40.1), 39.2 (38.4, 39.9), 38.9 (38.3, 39.6), and 39.2 (38.4, 40.1) in the top quintile of AHEI, AMED, hPDI, DASH, and DRRD, respectively (table S9). Compared to participants with the lowest dietary scores, individuals in the highest quintile of dietary scores gained years of life at age 45 ranging from 1.9 (95% CI: 0.6 to 3.4) to 3.0 (95% CI: 1.9 to 4.5) years in man and 1.5 (95% CI: 0.2 to 3.0) to 2.3 (95% CI: 0.5 to 3.0) years in women (Fig. 3). Among participants with high PRS, life expectancy at age 45 years was 1.4 (95% CI: 0.5 to 2.3) years longer for men and 1.7 (95% CI: 0.7 to 2.7) years longer for women than those with low PRS (table S10). Combining dietary scores and PRS, the gained years of life at 45 years for men with high PRS and the highest quintile of dietary score were 2.5 (95% CI: 0.5 to 4.6), 1.0 (95% CI: −1.0 to 3.1), 1.3 (95% CI: −1.1 to 3.5), 1.5 (95% CI: −0.6 to 3.8), and 3.2 (95% CI: 1.0 to 5.6), for AHEI, AMED, hPDI, DASH, and DRRD compared with the combination of low PRS and the lowest quintile of dietary score, respectively. For women with high PRS and the highest quintile of dietary score, the life expectancy was 2.4 (95% CI: 0.2 to 4.5), 4.2 (95% CI: 2.2 to 6.5), 4.0 (95% CI: 1.6 to 6.4), 2.4 (95% CI: 0.3 to 4.6), and 5.5 (95% CI: 3.2 to 7.8) years longer for AHEI, AMED, hPDI, DASH, and DRRD compared with the combination of low PRS and the lowest quintile of dietary score, respectively (fig. S7).

### Sensitivity analyses

Associations between dietary scores and all-cause mortality were not significantly modified by age, sex, obesity, smoking status, energy intake, and physical activity. However, the inverse association between dietary scores and all-cause mortality was stronger in more deprived participants, the association between DASH and all-cause mortality was more pronounced in older participants, and the association between DRRD and all-cause mortality was stronger among former or current smokers (table S11). The significant inverse association between dietary scores and all-cause mortality remained largely unchanged after further adjusting for smoking pack years, self-reported health status, and food groups not included in each dietary pattern, or when not adjusting for total energy intake (table S12). When restricting to never smokers, the association with all-cause mortality was slightly weaker for DRRD but slightly stronger for the other four dietary scores (table S13). The magnitude of associations between all dietary scores and all-cause mortality tended to be stronger when further excluding participants with diabetes at baseline (table S14), when excluding those who reported their diet as not typical at any of the five dietary recall occasions (table S15), and when restricting to participants with three or more dietary assessments (table S16), and the results remained largely unchanged after further excluding those with less than 2 years of follow-up (table S17). Removing alcohol from AHEI and AMED did not alter their associations with all-cause mortality significantly (table S18). For all food items across dietary scores, fiber intake and sugar-sweetened beverages (SSBs) showed the strongest inverse and positive association with all-cause mortality, respectively (fig. S3). For food substitution, poultry intake and SSB consumption had the lowest and highest risk of all-cause mortality when substituting for all other food groups, respectively (fig. S8). For cause-specific mortality, the association remained stable when accounting for the competing risks using the Fine-Gray model (tables S19 to S23). In addition, the life expectancy gained remained largely unchanged when we started the life table at ages 50, 55, and 60 years (figs. S9 to S11).

## DISCUSSION

In this longitudinal study of 103,649 participants, we found a significant association of greater adherence to various healthy dietary patterns with reduced all-cause mortality and prolonged life expectancy. After adjusting for multiple confounding factors, a healthier dietary pattern was associated with 18 to 24% lower risk of all-cause mortality, contributing to an additional life expectancy of 1.5 to 2.3 years for women and 1.9 to 3.0 years for men at the age of 45 years. Such associations remained robust regardless of carrying longevity genes.

### Comparison with other studies

Our results on associations between individual dietary scores and all-cause mortality were generally consistent with previous studies conducted in the Nurses’ Health Study (NHS), the Health Professionals Follow-up Study (HPFS), and other cohorts (*5*, *17*–*20*). In the NHS and HPFS, Shan *et al.* (*5*) reported that multivariable-adjusted HRs of all-cause mortality were 0.80 (95% CI: 0.77 to 0.82) for AHEI, 0.82 (95% CI: 0.79 to 0.84) for AMED, and 0.86 (95% CI: 0.83 to 0.89) for hPDI when comparing the highest quintile with the lowest, with a median follow-up of 36 years. The long follow-up and the use of cumulative mean dietary scores in NHS and HPFS contributed to their relatively narrower CIs. Similarly, in the Multiethnic Cohort, the highest quintile of AHEI, AMED, and DASH was associated with a 22, 24, and 19% lower risk of all-cause mortality, respectively (*17*). In addition, the magnitude and direction of the inverse association between DRRD and total mortality were also consistent with a recent report conducted in the US older population, which found a 24% lower risk of all-cause mortality in the top quintile compared to the bottom one (*20*). Among five dietary indices, DRRD showed the strongest association with all-cause mortality, which aligned with the findings of Wang *et al.* (*6*). Statistically, this result can be partly explained by the direct inclusion of dietary fiber intake and glycemic index in the DRRD scoring, as dietary fiber intake showed the strongest association with all-cause mortality and dietary glycemic index was also significantly associated with all deaths. Another possible explanation is that a diet particularly effective in improving insulin sensitivity may have greater potential to prevent chronic conditions and premature death, as insulin sensitivity plays a crucial role in the development and progression of chronic diseases (*21*).

Evidence regarding the associations between various dietary patterns and life expectancy remained limited. Using data from the NHS and the HPFS and the life table method, Li *et al.* (*22*) reported an approximately 4.7 years longer life expectancy at age 50 years for men and 3.8 years for women with the highest quintile of AHEI compared to the lowest. The association was stronger than our results, but the relatively longer life expectancy among males than females was consistent with our findings. However, in this study, AHEI was treated as a component of a healthy lifestyle, and other lifestyle factors were not adjusted for when modeling the association between AHEI and all-cause mortality. Consequently, when constructing a life table using a relative overestimated HR, the estimated life expectancy is likely to be overestimated. In another analysis conducted in the Whitehall II study, participants with the healthiest diet (the highest fifth of AHEI) lived 2.5 cardiometabolic disease-free years longer than those with the unhealthiest diet (the lowest fifth) at age 50 years (*23*). In the Whitehall II study, the interquartile range (IQR) of AHEI in the bottom quintile was 22 to 44, while the IQR of AHEI in the same quintile was 41 to 48 in our study, indicating a generally higher dietary quality for participants in the UK Biobank and shedding light on the slightly lower life expectancy observed in the UK Biobank.

Besides AHEI, significantly prolonged life expectancy of participants with the highest quintile than the lowest was observed for AMED, hPDI, DASH, and DRRD in our analysis, while no previous studies have investigated the association between these dietary scores and life expectancy. Although AMED and hPDI were based on tradition and personal preference and DASH and DRRD were established to reduce the risk of hypertension or diabetes, they were all associated with lower risk of major chronic diseases, such as CVD, stroke, cancer, type 2 diabetes, and others (*6*, *11*), which might explain the extended life expectancy for those with a healthier dietary pattern. On the one hand, our study provided quantitative data and filled the gap in the knowledge of the impact of multiple healthy dietary patterns on life span. On the other hand, further studies are needed to distinguish the gained years free of chronic diseases from the overall prolonged life expectancy.

Genetic factors are important contributors to all-cause mortality alongside environmental factors like diet (*24*), while few studies have investigated the interplay between diet and longevity genes on mortality and life expectancy. Previous studies on the Chinese elderly reported significant interactions between individual food group consumption and single genotype on mortality, indicating a significant modification of healthier diet intake on the impact of genetic risk on short life span (*25*, *26*). However, single food groups could not capture an individual’s diet quality and a locus mutation might only explain a small part of genetic risk (*27*). In our study, we calculated five dietary scores to holistically evaluate the diet quality and constructed a PRS using 19 single-nucleotide polymorphisms (SNPs) to estimate the genetic predisposition to longer life span and performed the interaction analysis to investigate the interplay between dietary scores and PRS. No significant multiplicative or additive interactions were observed in AHEI, AMED, hPDI, and DASH, and the associations between these four dietary scores and all-cause mortality were generally consistent across genetic strata, indicating that a healthy dietary pattern was beneficial to a longer life expectancy regardless of whether individuals carry longevity genes. For DRRD, the association was significantly stronger in individuals with low longevity PRS (indicating a shorter life span), as several SNPs are involved in insulin regulation, BMI, and lipid metabolism—factors closely linked to diabetes development (*12*). Our findings suggest that for those with a genetic predisposition to a shorter life span, DRRD may offer a potential strategy for extending life expectancy.

Several mechanisms may underlie the beneficial effects of healthy dietary patterns. First, all the dietary patterns emphasize the consumption of vegetables, fruits, and whole grains, which are rich in dietary fiber, flavonoids, and other antioxidants (*28*, *29*). These components may contribute to improved metabolic regulation, reduced inflammation, and maintenance of gut microbiota homeostasis (*30*, *31*). Consistently, we observed the strongest association between dietary fiber intake and reduced all-cause mortality, which may also partly explain the strongest association of all-cause mortality observed in DRRD. In addition, the antioxidant and metabolic regulatory potential of dietary patterns, particularly the DRRD, partly explain the stronger association between DRRD and all-cause mortality observed in former or current smokers compared to never smokers, as smoking is known to increase oxidative stress, inflammation, and metabolic dysregulation (*32*). Second, the encouraged intake of nuts and unsaturated fatty acids aids in the cardiometabolic processes (*33*). Third, these dietary patterns discourage the consumption of SSBs, which can promote hepatic de novo lipogenesis and insulin resistance and disrupt the gut microbiota, thereby impairing systemic metabolism (*34*, *35*). Consistently, we observed the strongest positive association between SSB and all-cause mortality in our analysis. Fourth, our results are consistent with previous reports showing that a higher dietary glycemic index was associated with increased all-cause mortality (*36*, *37*). This association may be attributed to the potential of high–glycemic index diets to induce blood glucose fluctuation and exacerbate insulin resistance, thereby contributing to the development of chronic diseases and premature death. Fifth, when distinguishing cause-specific mortality from all-cause mortality, we observed significant inverse associations in cancer, respiratory, and other-cause mortality, with the strongest inverse association observed for respiratory mortality. The stronger association between dietary scores and respiratory mortality may be explained by the dietary regulation of mucosal immunity, especially dietary fiber (*38*). In contrast, the mechanisms underlying the association with cancer mortality require further investigation, especially regarding whether improved insulin sensitivity contributes to this relationship, given the superior predictive performance of the DRRD for both all-cause and cause-specific mortality.

### Strengths and limitations of this study

Strengths of this study included the large sample size, relatively long follow-up, using a validated dietary recall method on at least two independent occasions to collect dietary data, and estimating the association between multiple dietary patterns and life expectancy. Several limitations should be acknowledged. First, the dietary information was collected on the basis of 24-hour recall, which might be subject to recall and reporting biases and lead to misclassification. However, this misclassification would likely bias the association toward null. Second, dietary data were collected only at baseline, and the long-term dietary characteristics and changes in diet quality over the follow-up duration were not evaluated. Third, the calculation of AMED, hPDI, DASH, and DRRD was based on the distribution of component intake in the study population, which might limit the generalization of the dietary pattern and life expectancy association. Fourth, as the association with all-cause mortality tended to be stronger among former/current smokers, the residual confounding by passive smoking likely overestimated the true association. Fifth, although our final model adjusted for a wide array of confounders and sensitivity analyses yielded robust results, residual confounding from factors such as health consciousness, health care accessibility, and other unmeasured variables cannot be fully excluded. Sixth, the effect sizes of alleles used in constructing PRS were derived from a population that overlaps with the UK Biobank. As a result, the effect size of PRS might be overestimated, and caution is needed when interpreting the genetic results. Seventh, although the PRS constructed from 19 SNPs cannot capture the complexity of longevity genetic architecture, our results provided valuable insights into the diet-gene interaction with longevity. Last, when conducting the genetic analysis, we only included participants of European descent, limiting the generalization of our results to other populations with a more diverse ethnic composition.

In conclusion, greater adherence to various healthy dietary patterns was consistently associated with a lower risk of all-cause mortality and longer life expectancy, regardless of longevity genes. Our results underscore the significance of adhering to healthy dietary patterns based on dietary recommendations for extending life expectancy, offering individuals the flexibility to adapt these dietary patterns according to their preferences and traditions.

## MATERIALS AND METHODS

The manuscript followed the Strengthening the Reporting of Observational Studies in Epidemiology guideline. The analysis plan was preregistered with the Open Science Foundation (https://osf.io/ev6sk/).

### Study design and population

The present study used data from the UK Biobank, a population-based prospective cohort study of more than half a million participants enrolled between 2006 and 2010 from 22 assessment centers across England, Scotland, and Wales. Detailed information on study design has been reported elsewhere (*39*). The UK Biobank study was approved by the North West Multi-centre Research Ethics Committee (REC reference: 21/NW/0157), and all participants provided informed consent at recruitment.

From April 2009 to June 2012, five separate dietary assessments were conducted using a web-based 24-hour dietary questionnaire (Oxford WebQ), validated against an interviewer-administered 24-hour recall (*40*) and biomarkers (*41*). We included participants who had completed two or more dietary assessments and excluded those with implausible energy intake (<800 or >4200 kcal/day in men and <600 or >3500 kcal/day in women) or those who were diagnosed with CVD or cancer at the last dietary questionnaire completion. Last, 103,649 participants were included in the main analysis. We further excluded those without genetic data or not of European descent for genetic analysis (*n* = 97,987), as the genome-wide association study referenced was predicated on Europeans (fig. S12).

### Dietary assessment

The consumption of each food was calculated by multiplying food portions by portion size (*Food Standards Agency food portion sizes, the third edition*). Nutrient intake for each food was derived by multiplying the quantity by nutrient content and then summing across all foods (*42*). The average intake of foods or nutrients from two or more dietary assessments was used in this analysis. We calculated AHEI, AMED, hPDI, DASH, and DRRD to evaluate the adherence to the alternate healthy eating pattern, the alternate Mediterranean diet pattern, the healthful plant-based pattern, the dietary pattern to stop hypertension, and the dietary pattern to reduce diabetes risk, respectively. The detailed components and criteria of each dietary score are shown in tables S24 to S28. Briefly, the AHEI-2010 contains 11 dietary components, each allocated with 0 to 10 points, with a range of 0 to 110, where higher scores indicate greater adherence to the healthy eating pattern (*7*, *43*). The AMED includes 10 food groups with one to five points assigned to each component according to sex-specific quintiles and ranges from 10 to 50, describing the Mediterranean diet pattern (*44*). The hPDI consists of 17 dietary components with 1 to 5 points assigned to each component by quintiles and ranges from 17 to 85 points, with a higher score indicating a healthier plant-based dietary pattern (*45*, *46*). The DASH score includes eight food groups with 1 to 5 points assigned to each item and ranges from 8 to 40 points, with a higher score indicating a lower risk of hypertension (*47*). The DRRD score assigns 1 to 5 points to each of the nine components by quintiles and ranges from 9 to 45 points, with a higher score indicating a lower risk of diabetes (*48*).

### Covariates

Potential confounders were selected according to a directed acyclic graph (fig. S13). Age was calculated as the duration from birth to the last dietary assessment (continuous). Sex (male/female), ethnicity (white/not-white), educational attainment (lower secondary, upper secondary, vocational, college or university, or others), and assessment centers (22 categories) were collected by a touch-screen questionnaire. Socioeconomic status was derived from the residential postcode and shown as the Townsend deprivation index (TDI; quintiles). Smoking status was self-reported and defined as never, former, and current. Physical activity was categorized according to metabolic equivalent of task minutes per week (MET-min/week, <600, 600 to 1199, or ≥1200 MET-min/week, or unknown). BMI was calculated with weight in kilograms divided by height in meters squared and categorized into <25.0, 25.0 to 29.9, or ≥30 kg/m^2^, or unknown. Total energy intake (kilocalories, continuous) and alcohol consumption (0, 0.1 to 10.0, 10.1 to 20.0, 20.1 to 35.0, or ≥35.1 g/day) were estimated by 24-hour dietary assessment. Baseline (i.e., the date when the most recent dietary recall was completed) dyslipidemia, hypertension, and diabetes were considered, and detailed definitions of these chronic diseases are shown in table S29.

### Outcome definition

Death information in the UK Biobank was provided by the National Health Service central register (England and Wales) and the National Records of Scotland (Scotland). The primary outcome of our analysis was all-cause mortality. The secondary outcome was cause-specific mortality ascertained by the International Classification of Diseases, 10th revision, including CVD mortality (I00-I99), cancer mortality (C00-C97), neurodegenerative disease mortality (G00-G99), respiratory disease mortality (J00-J99), and all other-cause mortality. Person-years at risk were calculated from the date of the most recent dietary assessment to the date of death, loss to follow-up, or 30 November 2022, whichever came first.

### PRS for longevity

A detailed description of the genotyping process, imputation, and quality control in the UK Biobank has been reported elsewhere (*49*). According to the genome-wide association study conducted among participants of European descent, 19 SNPs exhibited genome-wide significant association with longevity (*12*). The details of selected SNPs are shown in table S30. The PRS of longevity was calculated as follows: PRS = β_1_ × SNP_1_ + β_2_ × SNP_2_ + … + β*_n_* × SNP*_n_*, where SNP*_n_* denoted the effect allele number of each SNP and the β represented the years of life gained per effect allele. Together, a higher PRS for longevity indicated a higher risk of a long life span.

### Statistical analysis

The characteristics of participants were presented across dietary patterns quintiles, with continuous variables in mean (SD) or median (IQR), and categorical variables in counts (percentage). We evaluated the association of five dietary scores with each other using Spearman’s correlation coefficients. The dose-response relationships between dietary scores and all-cause and cause-specific mortality were flexibly modeled using restricted cubic splines with three knots (10th, 50th, and 90th) distributed across each of the dietary scores. Departure from linearity was examined by the Wald test. The Cox proportional hazard regression model with follow-up time as the time scale was used to estimate the HR and 95% CI of all-cause mortality and cause-specific mortality, using the lowest quintile of dietary pattern scores as the reference group. The proportional assumption was tested by the Schoenfeld residuals method and was not violated. We constructed two models to control the confounding effect: Model 1 was adjusted for age and sex, while model 2 was additionally adjusted for race, education, socioeconomic status, assessment centers, smoking status, physical activity, BMI, total energy intake, PRS, the first 10 principal components of ancestry, genotype measurement batches, baseline dyslipidemia, hypertension, and diabetes. For dietary scores excluding alcohol in their components (i.e., hPDI, DASH, and DRRD), we further adjusted for alcohol consumption in model 2. Test for trend was conducted by modeling the median of each dietary score as a continuous variable. We also calculated the association between the per SD increment in each dietary score and all-cause mortality.

Life expectancy among participants with varying dietary scores was calculated using the life table method, beginning at age 45 and extending to 100 years in single-year intervals. Cumulative survival probabilities from age 45 onward were calculated on the basis of the sex- and age-specific population mortality rates in the UK (*50*), the sex-specific HRs from the final model of all-cause mortality in exposure groups (quintiles 2 to 5 of the five dietary scores) compared to the reference group (quintile 1), and the sex- and age-specific prevalence of each exposure groups within categorized 10-year intervals. Years of life gained because of a healthier dietary pattern were estimated as the difference in life expectancy between each exposure group and the reference group at any given age. Detailed methodology regarding the estimation of life expectancy and years of life gained is shown in Supplementary Text.

We tested the effect modification of PRS on associations between dietary scores and all-cause mortality by including a multiplicative interaction term in the fully adjusted model, and the interaction was assessed through likelihood ratio tests. Additive interactions between dietary scores and PRS were conducted using SAS macro %GbyE (*51*). We then performed stratified analyses by PRS tertiles (low, intermediate, and high). Furthermore, we examined the joint association of dietary scores and genetic risk with all-cause mortality by defining a combined variable based on dietary score quintiles and PRS tertiles (15 groups), with the highest risk combination (the lowest dietary score and low PRS) serving as the reference group.

To explore which food group would explain the associations, we assessed the relationships between food groups and dietary scores using Spearman’s correlation. We then estimated the associations of food groups with all-cause mortality using Cox regression, treating food groups as continuous variables (per SD increment) and adjusting for covariates mentioned above.

We conducted stratified analyses by potential risk modifiers (age, sex, TDI, obesity, smoking, energy intake, and physical activity), with potential interactions assessed through likelihood ratio tests. Several sensitivity analyses were performed to examine the robustness of our results: (i) further adjusting for smoking pack years to account for potential residual confounding effects of smoking; (ii) restricting the analysis to never smokers to minimize the smoking-related confounding; (iii) further adjusting for baseline self-reported general health (self-rated overall health, weight change compared with 1 year ago, and number of medications taken) to limit the confounding effects of overall health status; (iv) not including total energy intake in the model to avoid the substitution of food groups not included in dietary scores; (v) further adjusting for food groups not included in each dietary pattern to control the possible confounding by other foods; (vi) using leave-one-out model to elaborate on the possible substitution involved (*52*); (vii) excluding participants who reported any of the dietary assessments as not typical to increase the diet representation; (viii) only including participants with three or more dietary assessments; (ix) reevaluating the associations of AHEI and AMED with all-cause mortality after removing alcohol from dietary scores, given the controversy over the health effect of moderate alcohol consumption (*53*); (x) excluding participants with less than 2 years of follow-up to account for the reverse causation caused by the dietary change before death; (xi) using the Fine-Gray model to account for competing risks between cause-specific mortalities; and (xii) recalculating the life expectancy by setting the beginning of the life table at ages 50, 55, and 60.

All statistical analyses were conducted using SAS (version 9.4) and R (version 4.3.1). A two-sided *P* value of less than 0.05 was considered statistically significant.

## Supplementary Materials

### This PDF file includes:

Supplementary Text Figs. S1 to S13 Tables S1 to S30 References
