# Supplementary material for: Healthy dietary patterns, longevity genes, and life expectancy: A prospective cohort study
Source: Sci Adv. 2026 Feb 13;12(7):eads7559. doi: 10.1126/sciadv.ads7559 (PMC12904179; doi:10.1126/sciadv.ads7559)
Supplement: Supplementary file 1 — Supplementary Text Figs. S1 to S13 Tables S1 to S30 References [file sciadv.ads7559_sm.pdf]

Science Advances
AAAS

Supplementary Materials for

Healthy dietary patterns, longevity genes, and life expectancy: A prospective cohort study

Yanling Lv *et al.*

Corresponding author: Liangkai Chen, clk@hust.edu.cn; Jing Song, jing.song@qmul.ac.uk

Sci. Adv. **12**, eads7559 (2026)
DOI: 10.1126/sciadv.ads7559

This PDF file includes:

Supplementary Text
Figs. S1 to S13
Tables S1 to S30
References

Supplementary Text

Life expectancy calculation

The life expectancy was calculated using the life table method. We built life tables starting at 45 years and ending at 100 years in single-year intervals using three parameters as follows(*54*): 1) sex- and age-specific population mortality in the UK (Office for National Statistics); 2) the sex-specific HR in the multivariable-adjusted model of all-cause mortality in exposure groups (quintiles 2 to 5 of five dietary scores) compared to the reference group (quintile 1); and 3) the sex- and age-specific (categorized into 10-year age group) prevalence of each exposure groups.

Based on the sex-specific HR, life tables were built for each quintile of five dietary patterns. Survival probability was set as 1 at the age of 45. The probability of surviving between age x and x+1 was calculated based on the probability of dying (mortality rate) between age x and x+1, with the assumption that the survival function declines linearly between ages x and x+1(*55*). The life expectancy at any given age was estimated by dividing the total person-years that would be lived beyond age x by the number of persons who survived to that age interval(*55*).

We used the algebraic formula to infer the sex- and age-specific mortality rates of the reference group (IR_0)(*56*):

$$IR_0 = \frac{IR_a}{p_{a0} + \sum_{j=1}^{4} p_{aj} \times HR_{aj}}$$

Where IR_a is the population mortality rate at the age group a, p_{aj} is the age-specific prevalence of exposure group j, and HR_{aj} is the HR for the exposure group j compared to the reference group (j=0). The age-specific mortality rate in each non-reference group was calculated by multiplying the age-specific mortality rate for the reference group IR_0 by HR_{aj}.

Finally, the estimated years gain due to a healthier dietary pattern was calculated as the difference in life expectancy between the reference group and each non-reference group at any given age. The CI of life expectancy was estimated using Monte Carlo simulation (parametric bootstrapping) with 10,00 runs.

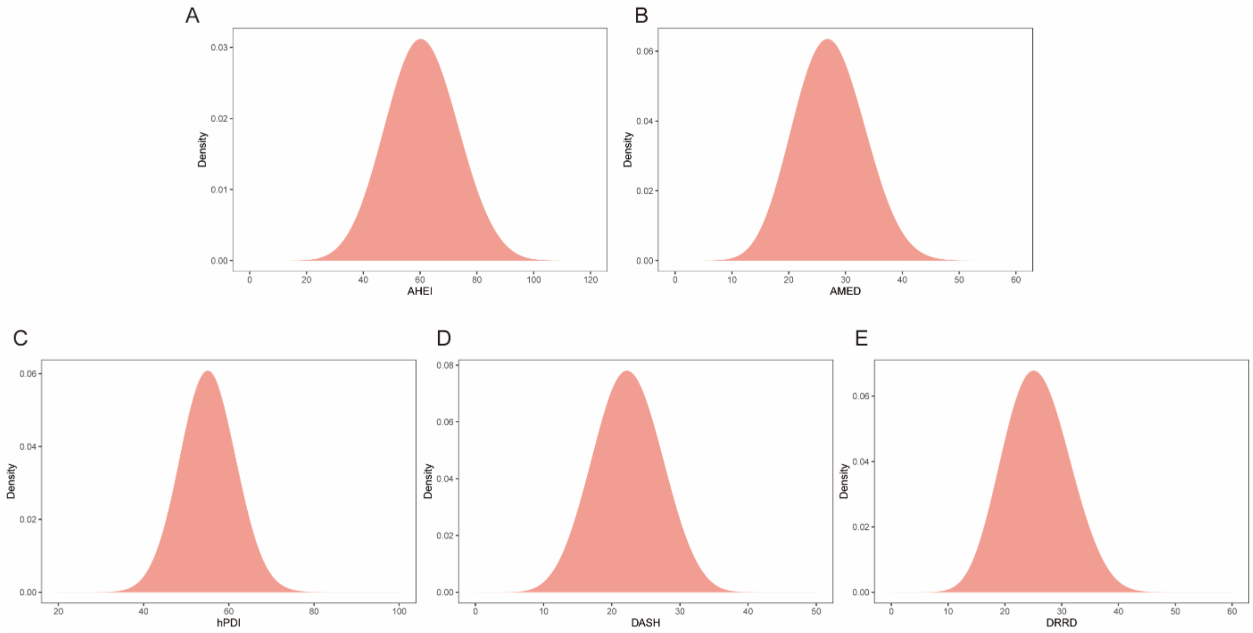

Fig. S1. Distribution of dietary scores
Abbreviations: AHEI, alternate healthy eating index; AMED; alternate Mediterranean diet; DASH, dietary approaches to stop hypertension; DRRD, diabetes risk reduction diet; hPDI, healthful plant-based diet.

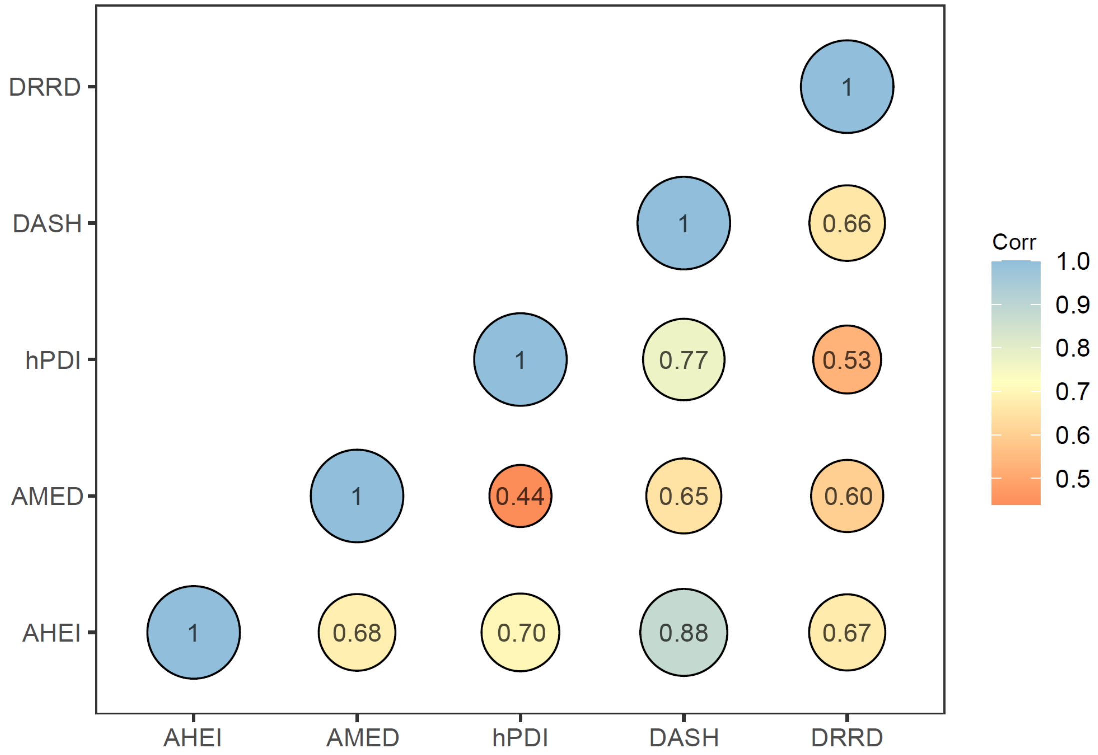

Fig. S2. The correlation between five dietary scores

Abbreviations: AHEI, alternate healthy eating index; AMED; alternate Mediterranean diet; DASH, dietary approaches to stop hypertension; DRRD, diabetes risk reduction diet; hPDI, healthful plant-based diet.

Food item	Correlation: AHEI	AMED	hPDI	DASH	DRRD	HR (95% CI) of all-cause mortality
Fruit	0.48	0.47	0.43	0.55	0.57	**0.95 (0.92, 0.98)**
Vegetables	0.44	0.45	0.38	0.49	0.32	**0.95 (0.92, 0.98)**
Nuts	0.31	0.36	0.25	0.26	0.39	**0.97 (0.93, 0.997)**
Legumes	0.34	0.37	0.31	0.34	0.17	0.99 (0.96, 1.02)
Whole grains	0.30	0.41	0.33	0.42	0.22	0.99 (0.96, 1.02)
Sodium	-0.36	0.06	-0.38	-0.38	-0.07	1.01 (0.97, 1.05)
Red and processed meat	-0.51	-0.36	-0.31	-0.51	-0.43	**1.04 (1.01, 1.07)**
Sugary drinks	-0.31	-0.10	-0.33	-0.32	-0.24	**1.07 (1.03, 1.10)**
Fruit juices	-0.18	0.09	-0.18	-0.14	-0.06	0.99 (0.96, 1.02)
Low fat dairy	0.04	0.12	-0.01	0.22	0.06	0.998 (0.97, 1.03)
Refined grains	-0.02	0.04	-0.21	-0.01	0.05	0.96 (0.93, 0.99)
Potatoes	-0.10	-0.01	-0.28	-0.12	-0.10	**1.04 (1.01, 1.07)**
Sweets and desserts	-0.11	-0.10	-0.33	-0.16	-0.08	**1.05 (1.02, 1.08)**
Animal fat	-0.14	-0.01	-0.32	-0.16	-0.09	1.02 (0.99, 1.05)
High fat dairy	-0.07	0.11	-0.17	-0.05	-0.14	1.03 (0.995, 1.06)
Eggs	-0.06	0.02	-0.17	-0.08	-0.01	0.97 (0.94, 1.001)
Fish and other seafood	0.10	0.35	-0.04	0.10	0.16	0.99 (0.96, 1.02)
Fibre	0.44	0.59	0.31	0.48	0.56	**0.90 (0.87, 0.94)**
Coffee	-0.05	0	0.03	-0.03	0.22	1.01 (0.98, 1.04)
Tea	0.14	0.08	0.23	0.13	-0.03	**0.96 (0.93, 0.99)**
Poultry	0	-0.04	-0.07	-0.02	0.02	**0.97 (0.94, 0.998)**
Glycemic Index	-0.08	-0.05	-0.02	-0.06	-0.33	**1.03 (1.003, 1.07)**
Ratio of MUFA to SFA	0.20	0.31	0.16	0.13	0.45	**0.95 (0.92, 0.98)**
Ratio of PUFA to SFA	0.37	0.40	0.28	0.29	0.58	**0.95 (0.92, 0.98)**
Energy proportion of trans fat	-0.17	-0.20	-0.16	-0.18	-0.52	1.02 (0.99, 1.05)
Long-chain (ω-3) fatty acids (EPA+DHA)	0.09	0.36	-0.13	0.03	0.22	**0.96 (0.93, 0.997)**
Energy proportion of ω-6 fatty acids	0.29	0.29	0.09	0.12	0.34	**0.94 (0.91, 0.97)**

Color scale: 0.5, 0, -0.5

Fig. S3. Correlations between food items and dietary scores and the associations of food items with all-cause mortality

The HR (95% CI) are shown as per SD increment of food items.

Adjusted for age (in years, continuous), sex (male/female), education (lower secondary, upper secondary, vocational, college or university, or others), TDI (in quintiles), assessment centers (22 categories), smoking (current, former, or never), physical activity (0-599, 600-1199, ≥1200 MET-mins/week, or unknown), BMI (<25.0, 25.0–29.9, ≥30 kg/m^2, or unknown), total energy intake (KCAL, continuous), baseline dyslipidemia (yes/no), hypertension (yes/no), diabetes (yes/no), longevity-PRS (in tertiles), top 10 principle genetic components (continuous), and genotype measurement batches (continuous).

Abbreviations: AHEI, alternate healthy eating index; AMED; alternate Mediterranean diet; BMI, body mass index; CI, confidence interval; DASH, dietary approaches to stop hypertension; DRRD, diabetes risk reduction diet; hPDI, healthful plant-based diet; HR, hazard ratio; MET, metabolic equivalent of task; PRS, polygenic risk score; SD, standard deviation; TDI, Townsend Deprivation Index.

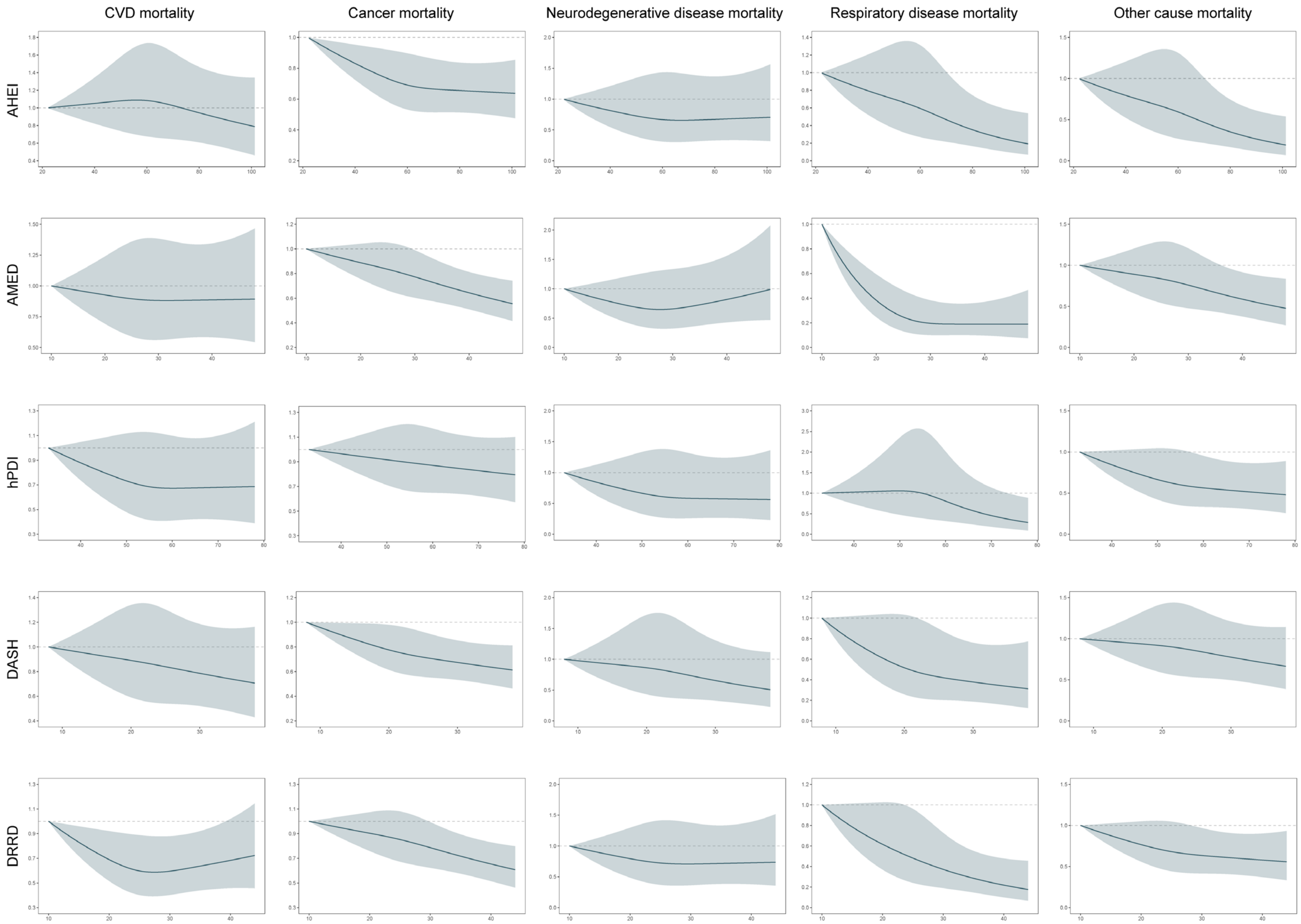

Fig. S4. The restricted cubic splines for the associations between dietary scores and cause-specific mortalities*

*Figures are shown as the multivariable-adjusted HR (95% CI).

Adjusted for age (in years, continuous), sex (male/female), education (lower secondary, upper secondary, vocational, college or university, or others), TDI (in quintiles), assessment centers (22 categories), smoking (current, former, or never), physical activity (0-599, 600-1199, ≥1200 MET-mins/week, or unknown), BMI (<25.0, 25.0–29.9, ≥30 kg/m^2, or unknown), total energy intake (KCAL, continuous), baseline dyslipidemia (yes/no), hypertension (yes/no), diabetes (yes/no), longevity-PRS (in tertiles), top 10 principle genetic components (continuous), and genotype measurement batches (continuous).

Abbreviations: AHEI, alternate healthy eating index; AMED; alternate Mediterranean diet; BMI, body mass index; CI, confidence interval; CVD, cardiovascular disease; DASH, dietary approaches to stop hypertension; DRRD, diabetes risk reduction diet; hPDI, healthful plant-based diet; HR, hazard ratio; MET, metabolic equivalent of task; PRS, polygenic risk score; TDI, Townsend Deprivation Index.

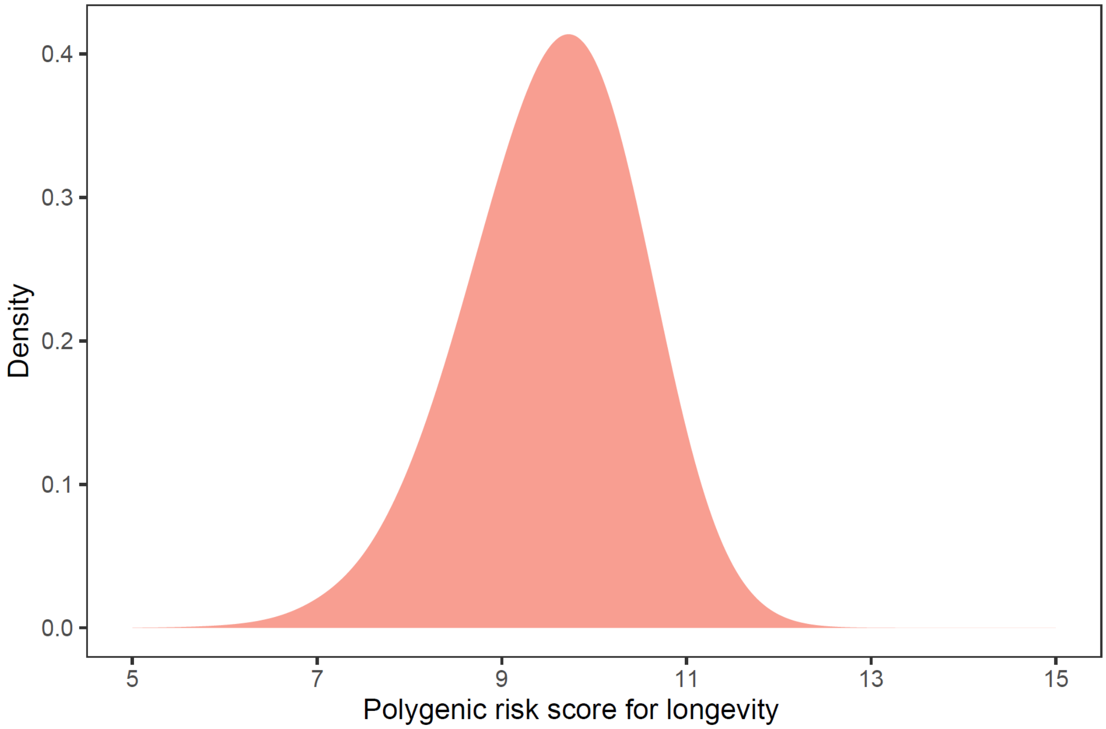

Fig. S5. The distribution of polygenic risk score for longevity

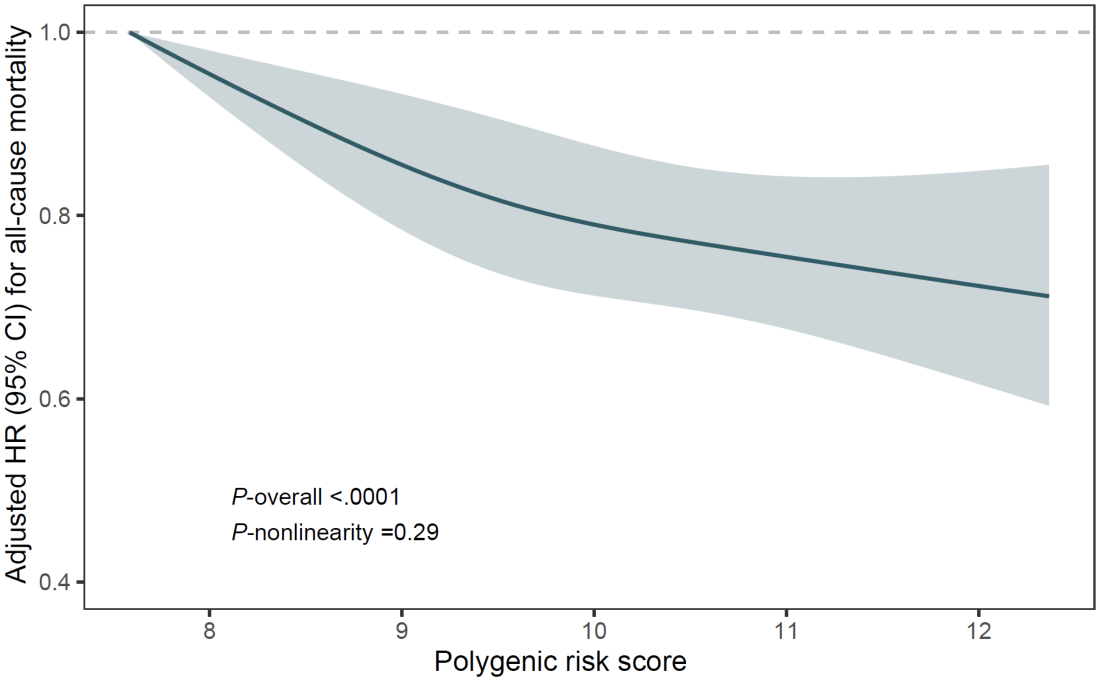

Fig. S6. The restricted cubic spline for the association between PRS of longevity and the risk of all-cause mortality*

Adjusted for age (in years, continuous), sex (male/female), education (lower secondary, upper secondary, vocational, college or university, or others), TDI (in quintiles), assessment centers (22 categories), smoking (current, former, or never), physical activity (0-599, 600-1199, ≥1200 MET-mins/week, or unknown), BMI (<25.0, 25.0–29.9, ≥30 kg/m^2, or unknown), total energy intake (KCAL, continuous), AHEI (in quintiles), baseline dyslipidemia (yes/no), hypertension (yes/no), diabetes (yes/no), top 10 principle genetic components (continuous), and genotype measurement batches (continuous).

Abbreviations: AHEI, alternate healthy eating index; BMI, body mass index; CI, confidence interval; MET, metabolic equivalent of task; PRS, polygenic risk score; TDI, Townsend Deprivation Index.

*: PRS was calculated using the effect size of each SNP, which was the years of life gained. Therefore, a higher PRS indicated a higher risk of a longer lifespan.

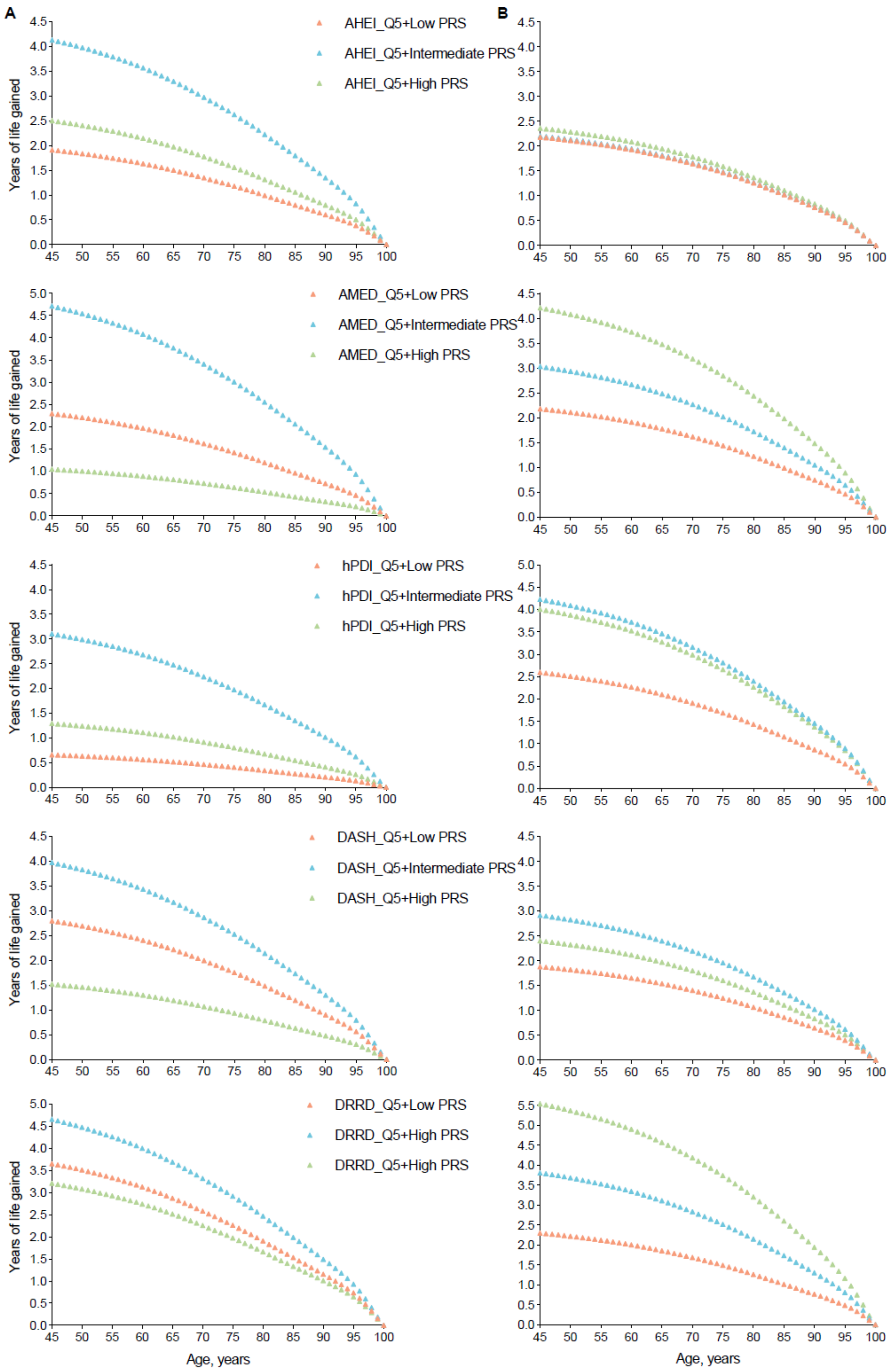

Fig. S7. Gained life expectancy from age 45 years onward among men (A) and women (B) by joint groups of dietary scores quintiles and longevity PRS tertiles

The results are shown as life gained compared to quintile 1 of dietary scores and low PRS. Abbreviations: AHEI, alternate healthy eating index; AMED, alternate Mediterranean diet; DASH, dietary approaches to stop hypertension; DRRD, diabetes risk reduction diet; hPDI, healthful plant-based diet; Q, quintile; PRS, polygenic risk score.

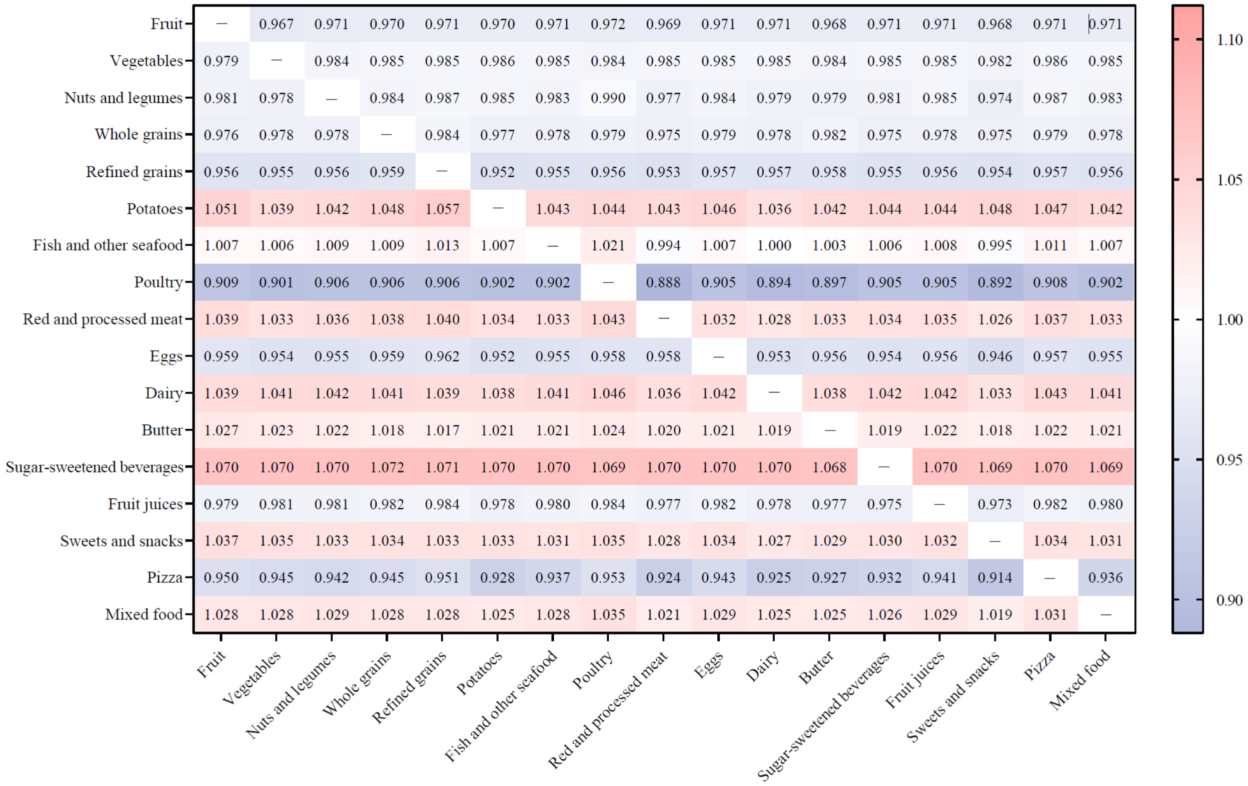

Fig. S8. Heatmap of substitution analysis of all food groups*

Adjusted for age (in years, continuous), sex (male/female), education (lower secondary, upper secondary, vocational, college or university, or others), TDI (in quintiles), assessment centers (22 categories), smoking (current, former, or never), physical activity (0-599, 600-1199, ≥1200 MET-mins/week, or unknown), BMI (<25.0, 25.0–29.9, ≥30 kg/m^2, or unknown), total energy intake (KCAL, continuous), baseline dyslipidemia (yes/no), hypertension (yes/no), diabetes (yes/no), top 10 principle genetic components (continuous), and genotype measurement batches (continuous).

*The value in each cell is the hazard ratio for all-cause mortality, derived from the model that substituted the food group on y-axis for the one on the x-axis.

Abbreviations: BMI, body mass index; CI, confidence interval; MET, metabolic equivalent of task; PRS, polygenic risk score; TDI, Townsend Deprivation Index.

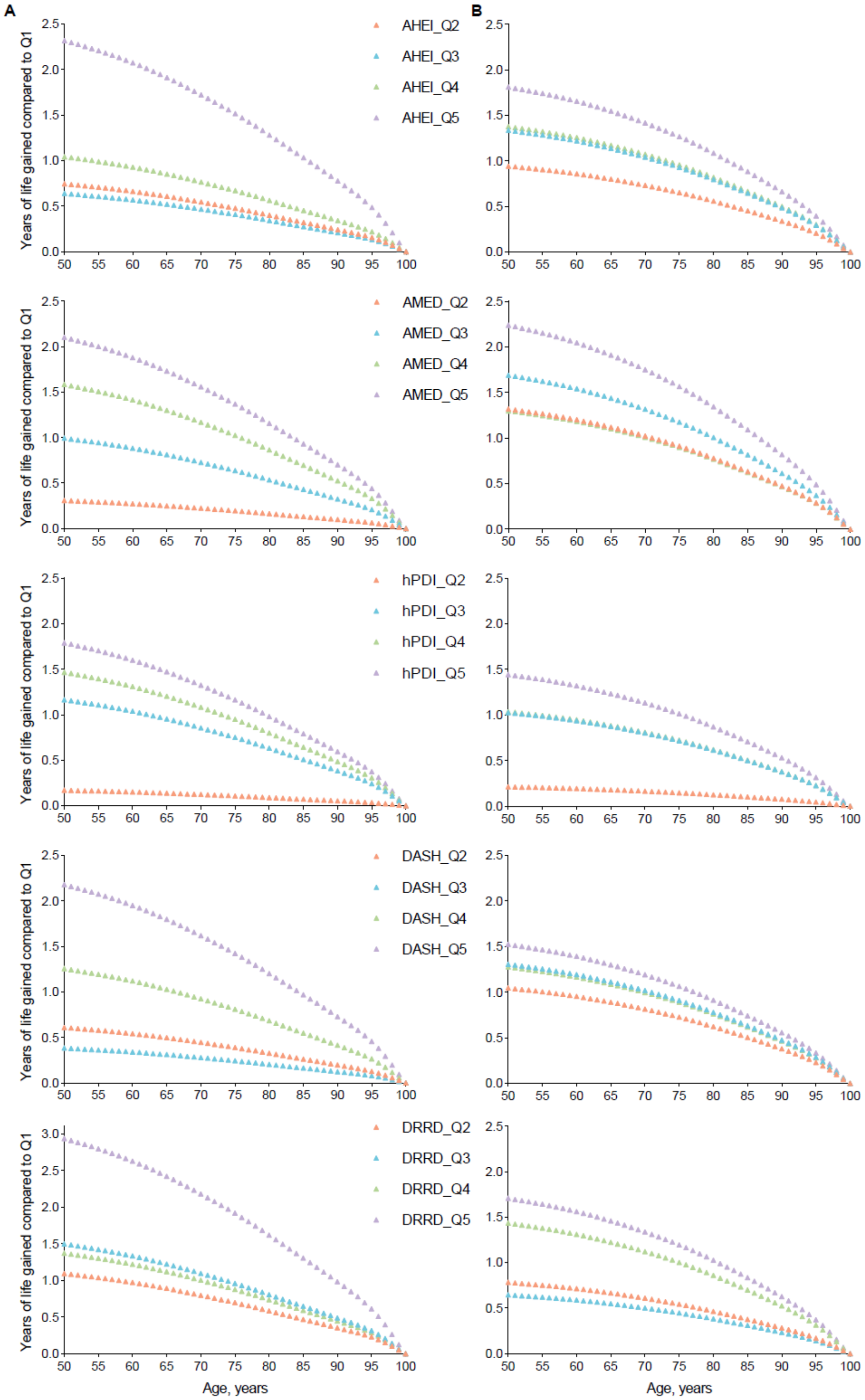

Fig. S9. Gained life expectancy from age 50 years onward among men (A) and women (B) by dietary scores quintiles

Abbreviations: AHEI, alternate healthy eating index; AMED, alternate Mediterranean diet; DASH, dietary approaches to stop hypertension; DRRD, diabetes risk reduction diet; hPDI, healthful plant-based diet; Q, quintile.

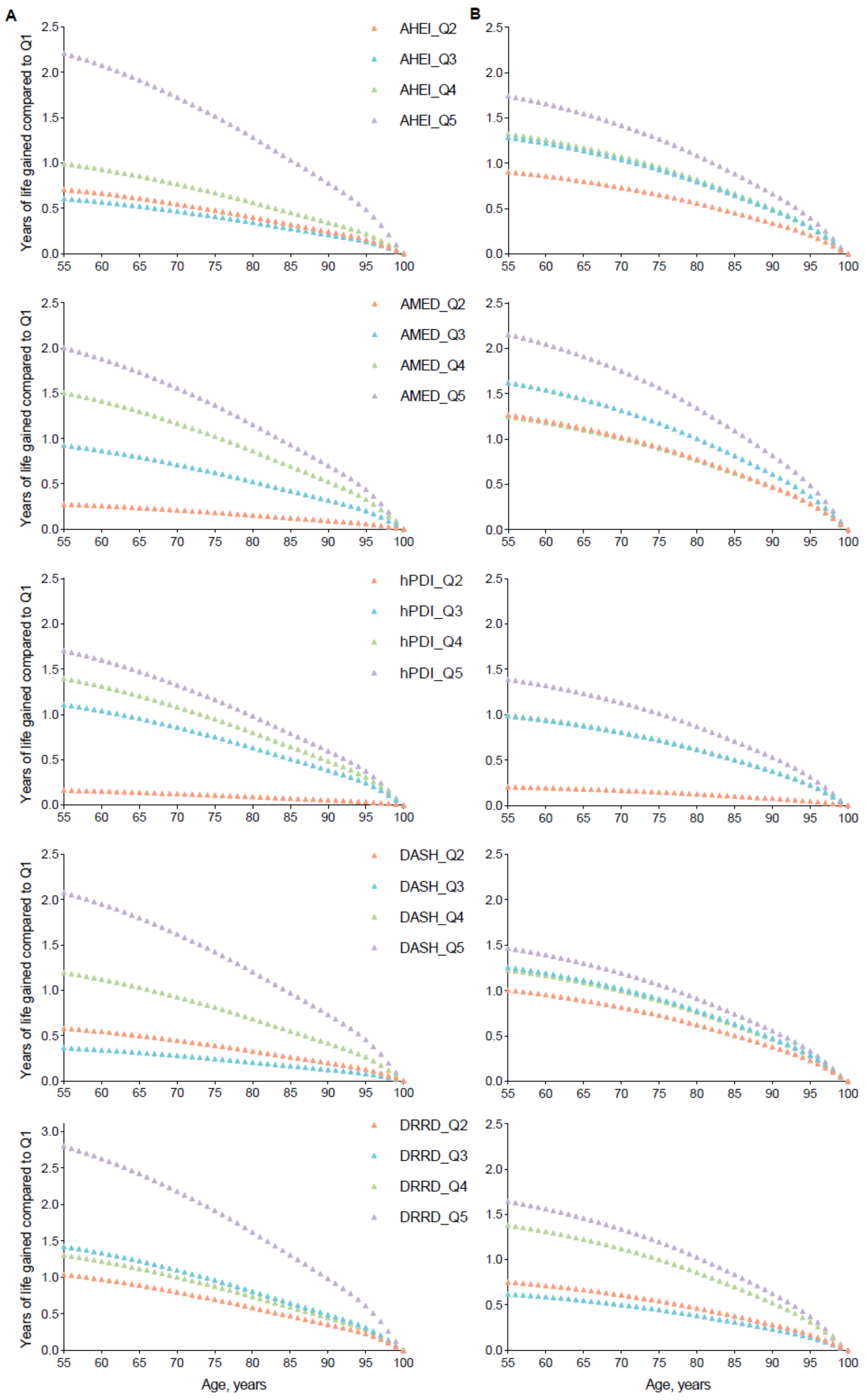

Fig. S10. Gained life expectancy from age 55 years onward among men (A) and women (B) by dietary scores quintiles

Abbreviations: AHEI, alternate healthy eating index; AMED, alternate Mediterranean diet; DASH, dietary approaches to stop hypertension; DRRD, diabetes risk reduction diet; hPDI, healthful plant-based diet; Q, quintile.

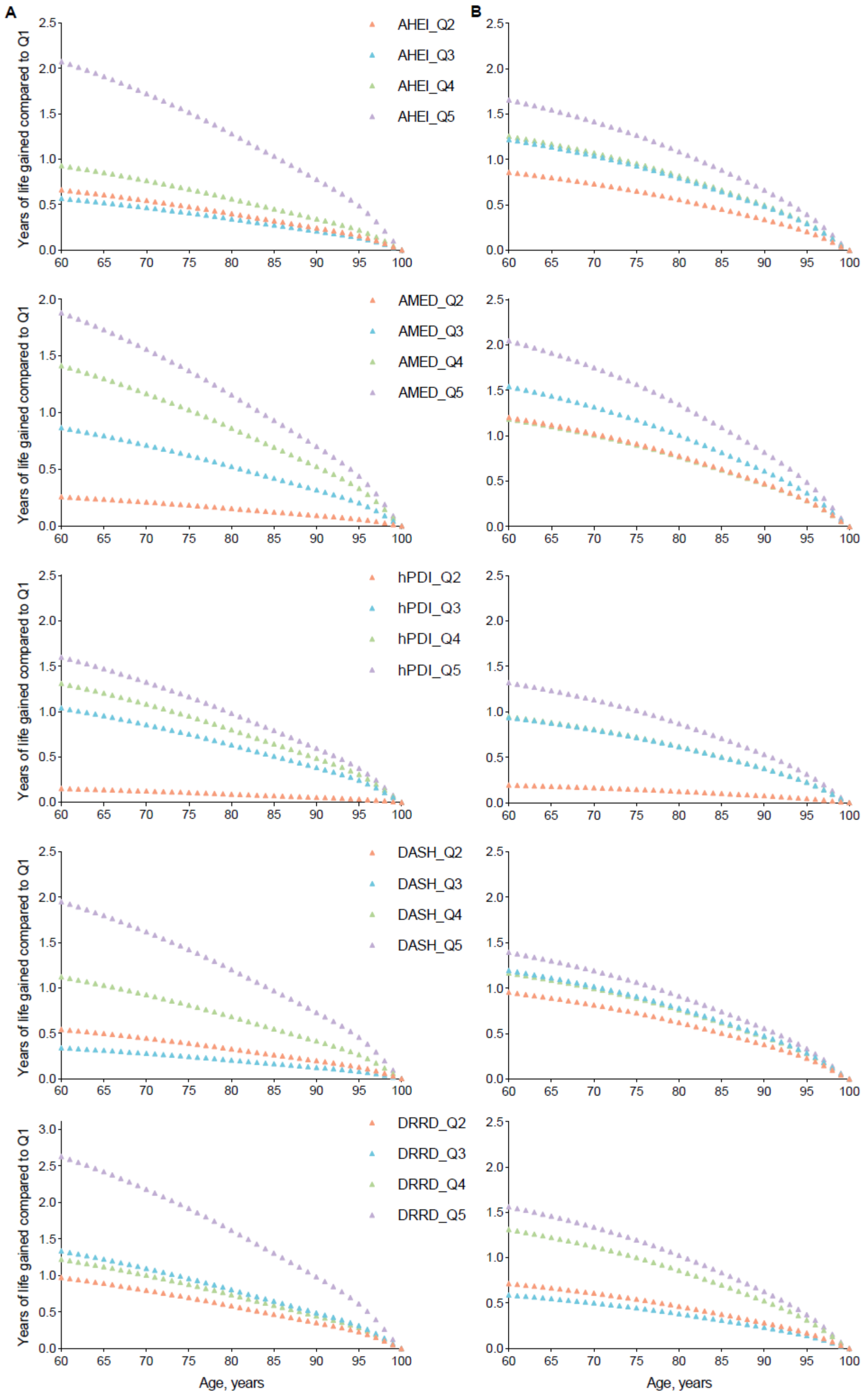

Fig. S11. Gained life expectancy from age 60 years onward among men (A) and women (B) by dietary scores quintiles

Abbreviations: AHEI, alternate healthy eating index; AMED, alternate Mediterranean diet; DASH, dietary approaches to stop hypertension; DRRD, diabetes risk reduction diet; hPDI, healthful plant-based diet; Q, quintile.

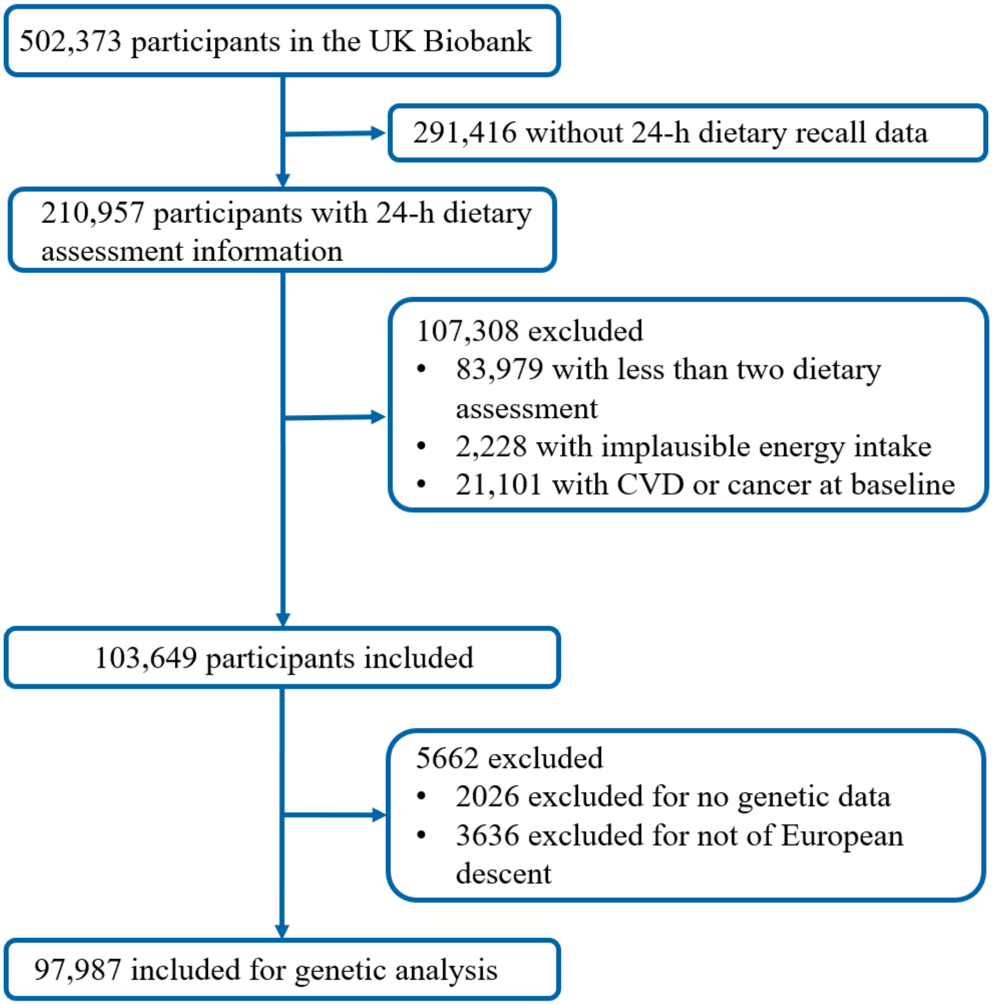

Fig. S12. Flow chart of participants included in the present UK Biobank study

Abbreviations: CVD, cardiovascular diseases

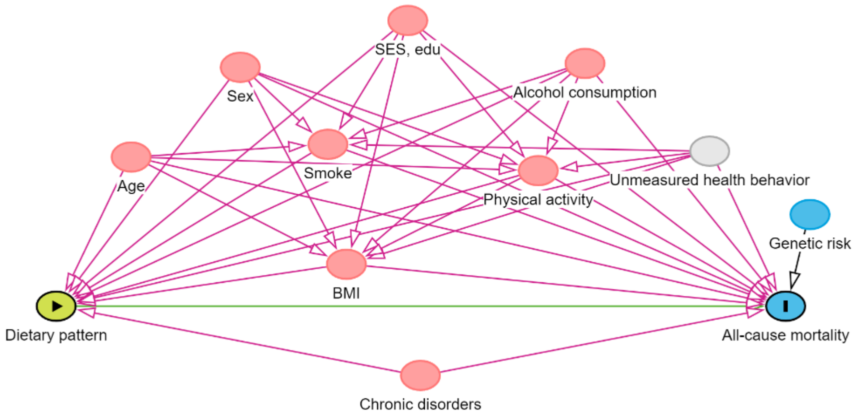

Fig. S13. Priori defined directed acyclic graph

Abbreviations: BMI, body mass index; SES; socioeconomic status

Table S1. Hazard ratio (95% CI) of cause-specific mortality associated with the alternate healthy eating index

	Quintile of AHEI					*P* for trend	per SD increment
	Quintile 1	Quintile 2	Quintile 3	Quintile 4	Quintile 5		
Median score	45 (41, 48)	54 (52, 56)	61 (59, 62)	67 (65, 69)	76 (73, 81)		
No. of participants	20,729	20,730	20,730	20,730	20,730		
Person-years	218,823	218,711	218,791	219,021	219,122		
CVD mortality							
Cases	172	156	160	151	126		
Model 1	1.00 (ref.)	0.86 (0.69, 1.06)	0.86 (0.69, 1.07)	0.82 (0.66, 1.03)	0.71 (0.56, 0.90)	0.0062	0.89 (0.83, 0.96)
Model 2	1.00 (ref.)	0.95 (0.76, 1.18)	0.995 (0.80, 1.24)	0.99 (0.79, 1.24)	0.90 (0.70, 1.15)	0.53	0.97 (0.90, 1.05)
Cancer mortality							
Cases	544	480	459	443	426		
Model 1	1.00 (ref.)	0.81 (0.72, 0.92)	0.74 (0.65, 0.84)	0.71 (0.62, 0.80)	0.67 (0.59, 0.77)	<.0001	0.86 (0.83, 0.90)
Model 2	1.00 (ref.)	0.88 (0.78, 0.997)	0.84 (0.74, 0.96)	0.83 (0.73, 0.95)	0.82 (0.72, 0.94)	0.0032	0.93 (0.89, 0.97)
Neurodegenerative mortality							
Cases	60	68	63	58	63		
Model 1	1.00 (ref.)	1.02 (0.72, 1.44)	0.90 (0.63, 1.28)	0.82 (0.57, 1.18)	0.88 (0.62, 1.27)	0.29	0.96 (0.85, 1.08)
Model 2	1.00 (ref.)	1.00 (0.70, 1.42)	0.90 (0.63, 1.29)	0.79 (0.54, 1.15)	0.86 (0.59, 1.25)	0.24	0.95 (0.84, 1.07)
Respiratory mortality							
Cases	65	49	54	40	26		
Model 1	1.00 (ref.)	0.68 (0.47, 0.99)	0.72 (0.50, 1.03)	0.53 (0.36, 0.79)	0.35 (0.22, 0.56)	<.0001	0.72 (0.63, 0.83)
Model 2	1.00 (ref.)	0.77 (0.53, 1.13)	0.84 (0.58, 1.22)	0.65 (0.43, 0.97)	0.45 (0.28, 0.73)	0.001	0.79 (0.69, 0.92)
Other mortality							
Cases	155	145	140	109	102		
Model 1	1.00 (ref.)	0.88 (0.70, 1.10)	0.83 (0.66, 1.04)	0.65 (0.50, 0.83)	0.62 (0.48, 0.79)	<.0001	0.85 (0.78, 0.92)
Model 2	1.00 (ref.)	0.95 (0.75, 1.19)	0.93 (0.73, 1.17)	0.75 (0.58, 0.97)	0.74 (0.57, 0.97)	0.0077	0.91 (0.84, 0.99)

Model 1 adjusted for age (in years, continuous) and sex (male/female);
Model 2 adjusted for covariates in model 1 plus ethnicity (white/not-white), education (lower secondary, upper secondary, vocational, college or university, or others), TDI (in quintiles), assessment centers (22 categories), smoking (current, former, or never), physical

activity (0-599, 600-1199, ≥1200 MET-mins/week, or unknown), BMI (<25.0, 25.0–29.9, ≥30 kg/m^2, or unknown), total energy intake (KCAL, continuous), baseline dyslipidemia (yes/no), hypertension (yes/no), diabetes (yes/no), longevity-PRS (in tertiles), top 10 genetic primary components (continuous), and genotype measurement batches (continuous).
Abbreviations: AHEI, alternate healthy eating index; BMI, body mass index; CI, confidence interval; CVD, cardiovascular disease; MET, metabolic equivalent of task; PRS, polygenic risk score; SD, standard deviation; TDI, Townsend Deprivation Index.

Table S2. Hazard ratio (95% CI) of cause-specific mortality associated with the alternate Mediterranean diet

	Quintile of AMED					*P* for trend	per SD increment
	Quintile 1	Quintile 2	Quintile 3	Quintile 4	Quintile 5		
Median score	20 (18, 21)	24 (23, 25)	27 (26, 28)	30 (29, 31)	35 (34, 37)		
No. of participants	21,530	19,865	20,674	21,741	19,839		
Person-years	227,792	209,644	218,227	229,324	209,481		
CVD mortality							
Cases	150	146	149	176	144		
Model 1	1.00 (ref.)	0.91 (0.72, 1.14)	0.83 (0.66, 1.04)	0.88 (0.71, 1.10)	0.74 (0.59, 0.94)	0.016	0.91 (0.85, 0.98)
Model 2	1.00 (ref.)	0.97 (0.77, 1.22)	0.94 (0.74, 1.18)	1.03 (0.83, 1.29)	0.92 (0.73, 1.17)	0.67	0.98 (0.91, 1.06)
Cancer mortality							
Cases	518	486	479	455	414		
Model 1	1.00 (ref.)	0.92 (0.81, 1.04)	0.83 (0.73, 0.94)	0.72 (0.64, 0.82)	0.69 (0.60, 0.78)	<.0001	0.87 (0.83, 0.90)
Model 2	1.00 (ref.)	0.97 (0.86, 1.10)	0.91 (0.81, 1.04)	0.81 (0.72, 0.92)	0.80 (0.70, 0.92)	<.0001	0.92 (0.88, 0.96)
Neurodegenerative mortality							
Cases	54	53	68	71	66		
Model 1	1.00 (ref.)	0.93 (0.64, 1.36)	1.08 (0.75, 1.54)	1.02 (0.71, 1.45)	0.98 (0.68, 1.41)	0.96	0.995 (0.89, 1.12)
Model 2	1.00 (ref.)	0.93 (0.64, 1.37)	1.09 (0.76, 1.56)	1.02 (0.71, 1.46)	0.99 (0.68, 1.44)	0.92	0.998 (0.89, 1.12)
Respiratory mortality							
Cases	70	50	41	38	35		
Model 1	1.00 (ref.)	0.65 (0.45, 0.93)	0.47 (0.32, 0.69)	0.39 (0.26, 0.58)	0.37 (0.24, 0.55)	<.0001	0.66 (0.58, 0.76)
Model 2	1.00 (ref.)	0.70 (0.49, 1.01)	0.54 (0.37, 0.80)	0.47 (0.31, 0.70)	0.47 (0.31, 0.71)	<.0001	0.73 (0.64, 0.84)
Other mortality							
Cases	150	144	121	129	107		
Model 1	1.00 (ref.)	0.92 (0.73, 1.15)	0.69 (0.55, 0.88)	0.67 (0.53, 0.85)	0.58 (0.45, 0.74)	<.0001	0.82 (0.76, 0.89)
Model 2	1.00 (ref.)	1.00 (0.80, 1.26)	0.81 (0.63, 1.03)	0.81 (0.64, 1.03)	0.75 (0.58, 0.97)	0.008	0.90 (0.83, 0.98)

Model 1 adjusted for age (in years, continuous) and sex (male/female);
Model 2 adjusted for covariates in model 1 plus ethnicity (white/not-white), education (lower secondary, upper secondary, vocational, college or university, or others), TDI (in quintiles), assessment centers (22 categories), smoking (current, former, or never), physical

activity (0-599, 600-1199, ≥1200 MET-mins/week, or unknown), BMI (<25.0, 25.0–29.9, ≥30 kg/m^2, or unknown), total energy intake (KCAL, continuous), baseline dyslipidemia (yes/no), hypertension (yes/no), diabetes (yes/no), longevity-PRS (in tertiles), top 10 genetic primary components (continuous), and genotype measurement batches (continuous).
Abbreviations: AMED; alternate Mediterranean diet; BMI, body mass index; CI, confidence interval; CVD, cardiovascular disease; MET, metabolic equivalent of task; PRS, polygenic risk score; SD, standard deviation; TDI, Townsend Deprivation Index.

Table S3. Hazard ratio (95% CI) of cause-specific mortality associated with the healthful plant-based diet

	Quintile of hPDI					*P* for trend	per SD increment
	Quintile 1	Quintile 2	Quintile 3	Quintile 4	Quintile 5		
Median score	47 (45, 48)	52 (51, 53)	55 (54, 56)	58 (57, 59)	63 (62, 65)		
No. of participants	18,984	22,484	19,840	22,331	20,010		
Person-years	200,289	236,866	209,446	236,034	211,832		
CVD mortality							
Cases	157	195	126	157	130		
Model 1	1.00 (ref.)	0.98 (0.79, 1.20)	0.71 (0.56, 0.90)	0.81 (0.65, 1.01)	0.78 (0.62, 0.99)	0.01	0.89 (0.83, 0.96)
Model 2	1.00 (ref.)	1.05 (0.84, 1.29)	0.79 (0.62, 1.001)	0.92 (0.73, 1.16)	0.92 (0.72, 1.18)	0.29	0.95 (0.87, 1.02)
Cancer mortality							
Cases	454	530	478	490	400		
Model 1	1.00 (ref.)	0.90 (0.80, 1.03)	0.89 (0.79, 1.02)	0.81 (0.71, 0.92)	0.75 (0.65, 0.86)	<.0001	0.92 (0.88, 0.96)
Model 2	1.00 (ref.)	0.96 (0.85, 1.09)	0.98 (0.86, 1.12)	0.91 (0.80, 1.04)	0.87 (0.75, 1.01)	0.043	0.97 (0.93, 1.01)
Neurodegenerative mortality							
Cases	65	69	69	53	56		
Model 1	1.00 (ref.)	0.80 (0.57, 1.12)	0.87 (0.62, 1.23)	0.60 (0.41, 0.86)	0.72 (0.50, 1.03)	0.026	0.94 (0.84, 1.06)
Model 2	1.00 (ref.)	0.79 (0.56, 1.11)	0.85 (0.60, 1.20)	0.57 (0.39, 0.83)	0.67 (0.45, 0.98)	0.012	0.92 (0.82, 1.05)
Respiratory mortality							
Cases	51	60	44	50	29		
Model 1	1.00 (ref.)	0.90 (0.62, 1.30)	0.73 (0.49, 1.09)	0.75 (0.51, 1.11)	0.51 (0.32, 0.81)	0.0028	0.81 (0.71, 0.93)
Model 2	1.00 (ref.)	0.97 (0.67, 1.42)	0.82 (0.54, 1.25)	0.85 (0.56, 1.27)	0.61 (0.37, 0.98)	0.039	0.86 (0.75, 0.995)
Other mortality							
Cases	137	160	135	122	97		
Model 1	1.00 (ref.)	0.92 (0.73, 1.16)	0.87 (0.68, 1.10)	0.71 (0.55, 0.90)	0.65 (0.50, 0.84)	0.0002	0.87 (0.80, 0.94)
Model 2	1.00 (ref.)	0.98 (0.78, 1.23)	0.94 (0.74, 1.21)	0.77 (0.60, 0.999)	0.72 (0.55, 0.95)	0.0054	0.90 (0.83, 0.98)

Model 1 adjusted for age (in years, continuous) and sex (male/female);
Model 2 adjusted for covariates in model 1 plus ethnicity (white/not-white), education (lower secondary, upper secondary, vocational, college or university, or others), TDI (in quintiles), assessment centers (22 categories), smoking (current, former, or never), physical

activity (0-599, 600-1199, ≥1200 MET-mins/week, or unknown), BMI (<25.0, 25.0–29.9, ≥30 kg/m^2, or unknown), alcohol consumption (0, 0.1–5, 5.1–10, 10.1–15, 15.1–20, 20.1–30, >30 g/day), total energy intake (KCAL, continuous), baseline dyslipidemia (yes/no), hypertension (yes/no), diabetes (yes/no), longevity-PRS (in tertiles), top 10 genetic primary components (continuous), and genotype measurement batches (continuous).
Abbreviations: BMI, body mass index; CI, confidence interval; CVD, cardiovascular disease; hPDI, healthful plant-based diet; MET, metabolic equivalent of task; PRS, polygenic risk score; SD, standard deviation; TDI, Townsend Deprivation Index.

Table S4. Hazard ratio (95% CI) of cause-specific mortality associated with dietary approaches to stop hypertension

	Quintile of DASH					*P* for trend	per SD increment
	Quintile 1	Quintile 2	Quintile 3	Quintile 4	Quintile 5		
Median score	16 (15, 18)	20 (19, 21)	22 (22, 23)	25 (24, 26)	29 (27, 30)		
No. of participants	22,185	22,939	16,949	21,701	19,875		
Person-years	234,222	241,870	179,063	229,151	210,162		
CVD mortality							
Cases	182	161	129	174	119		
Model 1	1.00 (ref.)	0.77 (0.62, 0.95)	0.83 (0.66, 1.04)	0.87 (0.70, 1.07)	0.68 (0.53, 0.86)	0.0084	0.89 (0.82, 0.96)
Model 2	1.00 (ref.)	0.84 (0.68, 1.05)	0.94 (0.75, 1.18)	1.01 (0.81, 1.25)	0.81 (0.63, 1.03)	0.31	0.95 (0.88, 1.02)
Cancer mortality							
Cases	536	577	361	468	410		
Model 1	1.00 (ref.)	0.92 (0.82, 1.03)	0.75 (0.66, 0.86)	0.74 (0.65, 0.84)	0.71 (0.62, 0.80)	<.0001	0.87 (0.83, 0.91)
Model 2	1.00 (ref.)	1.002 (0.89, 1.13)	0.84 (0.74, 0.97)	0.85 (0.75, 0.97)	0.83 (0.73, 0.96)	0.001	0.93 (0.89, 0.97)
Neurodegenerative mortality							
Cases	59	81	52	65	55		
Model 1	1.00 (ref.)	1.13 (0.81, 1.59)	0.95 (0.65, 1.38)	0.90 (0.63, 1.29)	0.83 (0.57, 1.21)	0.16	0.92 (0.82, 1.04)
Model 2	1.00 (ref.)	1.12 (0.80, 1.57)	0.93 (0.64, 1.36)	0.86 (0.60, 1.24)	0.77 (0.53, 1.14)	0.073	0.90 (0.80, 1.02)
Respiratory mortality							
Cases	69	49	40	39	37		
Model 1	1.00 (ref.)	0.59 (0.41, 0.85)	0.63 (0.43, 0.94)	0.47 (0.32, 0.70)	0.50 (0.33, 0.76)	0.0003	0.75 (0.66, 0.86)
Model 2	1.00 (ref.)	0.67 (0.46, 0.97)	0.76 (0.51, 1.13)	0.57 (0.38, 0.86)	0.64 (0.42, 0.97)	0.016	0.83 (0.72, 0.95)
Other mortality							
Cases	147	181	91	129	103		
Model 1	1.00 (ref.)	1.08 (0.87, 1.34)	0.72 (0.56, 0.94)	0.79 (0.62, 1.01)	0.70 (0.54, 0.91)	0.0007	0.88 (0.81, 0.95)
Model 2	1.00 (ref.)	1.21 (0.97, 1.51)	0.83 (0.63, 1.08)	0.93 (0.73, 1.19)	0.84 (0.65, 1.10)	0.065	0.94 (0.87, 1.02)

Model 1 adjusted for age (in years, continuous) and sex (male/female);
Model 2 adjusted for covariates in model 1 plus ethnicity (white/not-white), education (lower secondary, upper secondary, vocational, college or university, or others), TDI (in quintiles), assessment centers (22 categories), smoking (current, former, or never), physical

activity (0-599, 600-1199, ≥1200 MET-mins/week, or unknown), BMI (<25.0, 25.0–29.9, ≥30 kg/m^2, or unknown), alcohol consumption (0, 0.1–5, 5.1–10, 10.1–15, 15.1–20, 20.1–30, >30 g/day), total energy intake (KCAL, continuous), baseline dyslipidemia (yes/no), hypertension (yes/no), diabetes (yes/no), longevity-PRS (in tertiles), top 10 genetic primary components (continuous), and genotype measurement batches (continuous).

Abbreviations: BMI, body mass index; CI, confidence interval; CVD, cardiovascular disease; DASH, dietary approaches to stop hypertension; MET, metabolic equivalent of task; PRS, polygenic risk score; SD, standard deviation; TDI, Townsend Deprivation Index.

Table S5. Hazard ratio (95% CI) of cause-specific mortality associated with diabetes risk reduction diet

	Quintile of DRRD					*P* for trend	per SD increment
	Quintile 1	Quintile 2	Quintile 3	Quintile 4	Quintile 5		
Median score	18 (17, 20)	23 (22, 24)	25 (25, 26)	28 (27, 29)	33 (32, 35)		
No. of participants	18,488	26,362	14,635	24,610	19,554		
Person-years	194,850	278,220	154,510	259,930	206,957		
CVD mortality							
Cases	161	184	119	166	135		
Model 1	1.00 (ref.)	0.75 (0.61, 0.93)	0.87 (0.68, 1.10)	0.72 (0.58, 0.90)	0.74 (0.59, 0.93)	0.011	0.90 (0.84, 0.97)
Model 2	1.00 (ref.)	0.80 (0.65, 0.99)	0.94 (0.74, 1.19)	0.80 (0.64, 1.00)	0.84 (0.66, 1.06)	0.16	0.94 (0.87, 1.01)
Cancer mortality							
Cases	453	623	331	557	388		
Model 1	1.00 (ref.)	0.90 (0.80, 1.02)	0.85 (0.74, 0.98)	0.84 (0.74, 0.95)	0.72 (0.63, 0.82)	<.0001	0.89 (0.86, 0.93)
Model 2	1.00 (ref.)	0.96 (0.85, 1.08)	0.91 (0.78, 1.04)	0.91 (0.81, 1.04)	0.80 (0.70, 0.92)	0.0017	0.93 (0.89, 0.97)
Neurodegenerative mortality							
Cases	60	74	53	63	62		
Model 1	1.00 (ref.)	0.79 (0.56, 1.11)	0.997 (0.69, 1.44)	0.70 (0.49, 0.996)	0.84 (0.59, 1.21)	0.27	0.97 (0.87, 1.09)
Model 2	1.00 (ref.)	0.77 (0.55, 1.09)	0.96 (0.66, 1.39)	0.67 (0.47, 0.96)	0.79 (0.55, 1.13)	0.14	0.95 (0.85, 1.07)
Respiratory mortality							
Cases	64	54	34	57	25		
Model 1	1.00 (ref.)	0.54 (0.38, 0.78)	0.60 (0.40, 0.91)	0.60 (0.42, 0.86)	0.33 (0.21, 0.52)	<.0001	0.72 (0.63, 0.82)
Model 2	1.00 (ref.)	0.59 (0.41, 0.85)	0.67 (0.44, 1.02)	0.70 (0.48, 1.004)	0.38 (0.24, 0.62)	0.0004	0.76 (0.66, 0.87)
Other mortality							
Cases	138	182	91	147	93		
Model 1	1.00 (ref.)	0.87 (0.70, 1.09)	0.78 (0.60, 1.01)	0.75 (0.59, 0.94)	0.59 (0.45, 0.77)	<.0001	0.86 (0.79, 0.93)
Model 2	1.00 (ref.)	0.95 (0.76, 1.18)	0.86 (0.66, 1.12)	0.86 (0.68, 1.08)	0.68 (0.52, 0.89)	0.0036	0.91 (0.84, 0.98)

Model 1 adjusted for age (in years, continuous) and sex (male/female);
Model 2 adjusted for covariates in model 1 plus ethnicity (white/not-white), education (lower secondary, upper secondary, vocational, college or university, or others), TDI (in quintiles), assessment centers (22 categories), smoking (current, former, or never), physical

activity (0-599, 600-1199, ≥1200 MET-mins/week, or unknown), BMI (<25.0, 25.0–29.9, ≥30 kg/m^2, or unknown), alcohol consumption (0, 0.1–5, 5.1–10, 10.1–15, 15.1–20, 20.1–30, >30 g/day), total energy intake (KCAL, continuous), baseline dyslipidemia (yes/no), hypertension (yes/no), diabetes (yes/no), longevity-PRS (in tertiles), top 10 genetic primary components (continuous), and genotype measurement batches (continuous).

Abbreviations: BMI, body mass index; CI, confidence interval; CVD, cardiovascular disease; DRRD, diabetes risk reduction diet; MET, metabolic equivalent of task; PRS, polygenic risk score; SD, standard deviation; TDI, Townsend Deprivation Index.

Table S6. Baseline characteristics of participants included in the main analysis and the genetic analysis

Characteristics	Main analysis (n=103,649)	Genetic analysis (n=97,987)
Age, mean (SD), y	58.3 (7.8)	58.5 (7.8)
Male, n (%)	45,171 (43.6)	42,846 (43.7)
White, n (%)	99,910 (96.4)	97,987 (100)
Townsend Deprivation Index, median (IQR)	-2.3 (-3.8, 0)	-2.4 (-3.8, -0.1)
Deprivation fifth, n (%)		
1 (least deprived)	20,694 (20.0)	19,962 (20.4)
2-4	62,127 (59.9)	59,200 (60.4)
5 (most deprived)	20,704 (20.0)	18,713 (19.1)
Unknown	124 (0.1)	112 (0.1)
Education		
College or university	49,698 (48.0)	46,663 (47.6)
Vocational	9770 (9.4)	9241 (9.4)
Upper secondary	14,076 (13.6)	13,447 (13.7)
Lower secondary	23,800 (23.0)	22,658 (23.1)
Others	6000 (5.8)	5750 (5.9)
Unknown	305 (0.3)	228 (0.2)
Alcohol consumption, median (IQR), g/day	11.2 (0, 26.0)	11.5 (0, 26.4)
Current smoker, n (%)	7139 (6.9)	6693 (6.8)
Body mass index, mean (SD), kg/m^2	26.6 (4.5)	26.6 (4.5)
Energy intake, mean (SD), kcal/d	2044.2 (467.7)	2048.0 (465.3)
Total physical activity, MET-mins/week		
0-599	16,078 (15.5)	15,130 (15.4)
600-1199	16,773 (16.2)	15,845 (16.2)
≥1200	56,313 (54.3)	53,322 (54.4)
Unknown	14,485 (14.0)	13,690 (14.0)
Dyslipidemia, n (%)	47,412 (45.7)	45,014 (45.9)
Hypertension, n (%)	51,000 (49.2)	48,306 (49.3)
Diabetes, n (%)	4175 (4.0)	3769 (3.9)
PRS, mean (SD)	17.6 (2.3)	17.5 (2.3)

Abbreviations: IQR, interquartile range; MET, metabolic equivalent of task; PRS, polygenic risk score; SD, standard deviation.

Table S7. Hazard ratio (95% CI) of death from all causes according to longevity PRS categories

	Cases/ person-years	Model 1	Model 2
**Low PRS**	1479/344,178	1.00 (reference)	1.00 (reference)
**Intermediate PRS**	1360/345,419	0.91 (0.84, 0.98)	0.90 (0.84, 0.97)
**High PRS**	1281/344,738	0.85 (0.79, 0.92)	0.85 (0.78, 0.91)
**Per SD increment**		0.93 (0.90, 0.96)	0.93 (0.90, 0.96)

Model 1 adjusted for age (in years, continuous) and sex (male/female);
Model 2 adjusted for covariates in model 1 plus education (lower secondary, upper secondary, vocational, college or university, or others), TDI (in quintiles), assessment centers (22 categories), smoking (current, former, or never), physical activity (0-599, 600-1199, ≥1200 MET-mins/week, or unknown), BMI (<25.0, 25.0–29.9, ≥30 kg/m^2, or unknown), AHEI (in quintiles), total energy intake (KCAL, continuous), baseline dyslipidemia (yes/no), hypertension (yes/no), diabetes (yes/no), top 10 genetic primary components (continuous), and genotype measurement batches (continuous).

Abbreviations: AHEI, alternate healthy eating index; BMI, body mass index; CI, confidence interval; MET, metabolic equivalent of task; PRS, polygenic risk score; SD, standard deviation; TDI, Townsend Deprivation Index.

Table S8. Stratified analyses of associations between dietary scores and all-cause mortality by PRS*

		AHEI	AMED	hPDI	DASH	DRRD
Low PRS	Quintile 1	1.00 (reference)	1.00 (reference)	1.00 (reference)	1.00 (reference)	1.00 (reference)
	Quintile 2	0.98 (0.84, 1.14)	0.93 (0.79, 1.09)	0.91 (0.77, 1.06)	1.01 (0.87, 1.18)	0.77 (0.66, 0.89)
	Quintile 3	0.91 (0.77, 1.06)	0.88 (0.75, 1.03)	0.86 (0.73, 1.02)	0.87 (0.73, 1.03)	0.80 (0.67, 0.95)
	Quintile 4	0.83 (0.70, 0.98)	0.83 (0.71, 0.97)	0.82 (0.70, 0.97)	0.88 (0.75, 1.03)	0.72 (0.62, 0.84)
	Quintile 5	0.81 (0.68, 0.97)	0.80 (0.67, 0.94)	0.76 (0.63, 0.91)	0.76 (0.64, 0.91)	0.66 (0.56, 0.79)
Intermediate PRS	Quintile 1	1.00 (reference)	1.00 (reference)	1.00 (reference)	1.00 (reference)	1.00 (reference)
	Quintile 2	0.83 (0.70, 0.97)	0.96 (0.82, 1.13)	0.97 (0.83, 1.14)	0.85 (0.73, 0.99)	0.91 (0.77, 1.06)
	Quintile 3	0.82 (0.69, 0.96)	0.94 (0.80, 1.11)	0.90 (0.76, 1.07)	0.76 (0.64, 0.90)	0.86 (0.71, 1.03)
	Quintile 4	0.72 (0.61, 0.86)	0.83 (0.70, 0.98)	0.78 (0.65, 0.93)	0.79 (0.67, 0.93)	0.88 (0.75, 1.04)
	Quintile 5	0.70 (0.58, 0.83)	0.72 (0.60, 0.86)	0.77 (0.64, 0.93)	0.70 (0.59, 0.84)	0.73 (0.61, 0.88)
High PRS	Quintile 1	1.00 (reference)	1.00 (reference)	1.00 (reference)	1.00 (reference)	1.00 (reference)
	Quintile 2	0.90 (0.76, 1.06)	0.98 (0.83, 1.16)	0.998 (0.84, 1.19)	1.08 (0.92, 1.27)	1.07 (0.90, 1.26)
	Quintile 3	0.91 (0.76, 1.08)	0.84 (0.70, 1.002)	1.01 (0.85, 1.21)	0.96 (0.80, 1.16)	1.09 (0.90, 1.32)
	Quintile 4	0.96 (0.80, 1.14)	0.86 (0.72, 1.03)	0.98 (0.81, 1.17)	0.90 (0.75, 1.08)	1.04 (0.87, 1.24)
	Quintile 5	0.94 (0.78, 1.13)	0.95 (0.80, 1.14)	1.01 (0.83, 1.23)	1.06 (0.89, 1.28)	0.96 (0.79, 1.16)
P for multiplicative interaction		0.21	0.46	0.12	0.12	0.037
P for additive interaction		0.34	0.37	0.52	0.11	0.043

Data are shown as HR (95% CI).
Adjusted for age (in years, continuous), sex (male/female), education (lower secondary, upper secondary, vocational, college or university, or others), TDI (in quintiles), assessment centers (22 categories), smoking (current, former, or never), physical activity (0-599, 600-1199, ≥1200 MET-mins/week, or unknown), BMI (<25.0, 25.0–29.9, ≥30 kg/m^2, or unknown), total energy intake (KCAL, continuous), baseline dyslipidemia (yes/no), hypertension (yes/no), diabetes (yes/no), top 10 principle genetic components (continuous), and genotype measurement batches (continuous); alcohol consumption (0, 0.1–5, 5.1–10, 10.1–15, 15.1–20, 20.1–30, >30 g/day) was additionally adjusted for hPDI, DASH, and DRRD.
*A higher PRS indicated a longer life expectancy.
Abbreviations: AHEI, alternate healthy eating index; AMED; alternate Mediterranean diet; BMI, body mass index; CI, confidence interval; DASH, dietary approaches to stop hypertension; DRRD, diabetes risk reduction diet; hPDI, healthful plant-based diet; HR, hazard ratio; MET, metabolic equivalent of task; PRS, polygenic risk score; TDI, Townsend Deprivation Index.

Table S9. Life expectancy at age 45 years (95% CI) according to quintiles of dietary scores

		Men		Women	
		LE at 45 years old (95% CI)	Years of life gained (95% CI)	LE at 45 years old (95% CI)	Years of life gained (95% CI)
**AHEI**					
	Quintile 1	34.0 (33.6, 34.8)	0 (reference)	37.3 (36.9, 37.9)	0 (reference)
	Quintile 2	34.8 (34.2, 35.6)	0.8 (-0.3, 1.8)	38.2 (37.6, 39.3)	1.0 (0.1, 2.8)
	Quintile 3	34.7 (34.4, 35.9)	0.7 (-0.1, 2.1)	38.7 (37.7, 39.3)	1.4 (0.2, 3.0)
	Quintile 4	35.1 (34.8, 36.4)	1.1 (0.3, 2.5)	38.7 (38.3, 39.9)	1.4 (0.9, 3.6)
	Quintile 5	36.4 (35.6, 37.7)	2.4 (1.1, 3.8)	39.1 (38.3, 39.6)	1.9 (0.6, 3.3)
**AMED**					
	Quintile 1	34.0 (33.2, 34.7)	0 (reference)	37.1 (36.8, 38.2)	0 (reference)
	Quintile 2	34.3 (33.4, 34.9)	0.3 (-1.1, 1.2)	38.4 (37.5, 39.0)	1.4 (-0.4, 2.0)
	Quintile 3	35.0 (34.2, 35.7)	1.0 (-0.2, 2.2)	38.8 (37.9, 39.6)	1.7 (0.1, 2.5)
	Quintile 4	35.6 (34.9, 36.4)	1.6 (0.4, 2.9)	38.4 (38.3, 39.9)	1.3 (0.4, 2.9)
	Quintile 5	36.2 (35.6, 37.1)	2.2 (1.0, 3.7)	39.4 (38.3, 40.1)	2.3 (0.5, 3.0)
**hPDI**					
	Quintile 1	34.0 (33.6, 34.9)	0 (reference)	37.7 (36.6, 38.6)	0 (reference)
	Quintile 2	34.2 (34.1, 35.4)	0.2 (-0.6, 1.5)	37.9 (37.0, 38.5)	0.2 (-1.2, 1.5)
	Quintile 3	35.3 (34.2, 35.7)	1.2 (-0.4, 1.8)	38.7 (37.7, 39.3)	1.1 (-0.4, 2.2)
	Quintile 4	35.6 (34.9, 36.5)	1.5 (0.2, 2.7)	38.7 (38.2, 39.7)	1.1 (0.01, 2.7)
	Quintile 5	35.9 (35.3, 37.3)	1.9 (0.6, 3.4)	39.2 (38.4, 39.9)	1.5 (0.2, 3.0)
**DASH**					
	Quintile 1	34.0 (33.6, 34.8)	0 (reference)	37.4 (36.0, 37.9)	0 (reference)
	Quintile 2	34.7 (34.2, 35.6)	0.6 (-0.3, 1.8)	38.4 (37.6, 39.3)	1.1 (0.1, 2.8)
	Quintile 3	34.4 (34.4, 35.9)	0.4 (-0.1, 2.1)	38.7 (37.7, 39.3)	1.3 (0.2, 3.0)
	Quintile 4	35.3 (34.8, 36.4)	1.3 (0.3, 2.5)	38.7 (38.3, 39.9)	1.3 (0.9, 3.6)
	Quintile 5	36.3 (35.6, 37.7)	2.3 (1.1, 3.8)	38.9 (38.3, 39.6)	1.6 (0.6, 3.3)
**DRRD**					
	Quintile 1	33.6 (33.1, 34.5)	0 (reference)	37.5 (36.4, 38.2)	0 (reference)
	Quintile 2	34.8 (34.3, 35.5)	1.1 (-0.0, 2.1)	38.3 (37.7, 39.1)	0.8 (-0.1, 2.4)
	Quintile 3	35.2 (34.5, 36.3)	1.6 (0.3, 2.8)	38.1 (36.9, 38.8)	0.7 (-0.9, 1.9)
	Quintile 4	35.1 (34.5, 35.8)	1.4 (0.1, 2.3)	38.9 (38.4, 39.8)	1.5 (0.4, 3.1)
	Quintile 5	36.7 (36.0, 38.0)	3.0 (1.9, 4.5)	39.2 (38.4, 40.1)	1.8 (0.6, 3.3)

The multivariable-adjusted HRs were used for LE calculation, and the 95% CIs were estimated using bootstrap with 1000 runs.

Abbreviations: AHEI, alternate healthy eating index; AMED; alternate Mediterranean diet; CI, confidence interval; DASH, dietary approaches to stop hypertension; DRRD, diabetes risk reduction diet; hPDI, healthful plant-based diet; HR, hazard ratio; LE, life expectancy.

Table S10. Life expectancy at age 45 years (95% CI) according to PRS categories*

PRS category	Men		Women	
	LE at 45 years old	Years of life gained	LE at 45 years old	Years of life gained
**Low PRS**	34.2 (33.8, 34.8)	0 (reference)	37.6 (37.1, 38.2)	0 (reference)
**Intermediate PRS**	35.2 (34.6, 35.7)	1.0 (-0.1, 1.8)	38.6 (38.0, 39.2)	1.1 (-0.1, 1.9)
**High PRS**	35.7 (35.2, 36.2)	1.4 (0.5, 2.3)	39.2 (38.7, 40.0)	1.7 (0.7, 2.7)

The multivariable-adjusted HRs were used for LE calculation, and the 95% CIs were estimated using bootstrap with 1000 runs.

*A higher PRS indicated a longer life expectancy.

Abbreviations: CI, confidence interval; LE, life expectancy; PRS, polygenic risk score.

Table S11. Stratified analyses of associations between per SD increment of dietary scores and all-cause mortality by major confounders

	AHEI	AMED	hPDI	DASH	DRRD
**Age, years**					
<60	1.004 (0.94, 1.07)	0.96 (0.91, 1.03)	1.03 (0.96, 1.10)	1.02 (0.96, 1.09)	0.98 (0.92, 1.04)
≥60	0.94 (0.90, 0.97)	0.93 (0.90, 0.97)	0.94 (0.91, 0.98)	0.93 (0.89, 0.96)	0.92 (0.89, 0.95)
P for interaction	0.14	0.71	0.08	0.02	0.15
**Sex**					
Male	0.93 (0.89, 0.97)	0.92 (0.88, 0.96)	0.94 (0.90, 0.98)	0.92 (0.88, 0.96)	0.91 (0.87, 0.95)
Female	0.92 (0.88, 0.97)	0.92 (0.88, 0.97)	0.95 (0.90, 1.001)	0.93 (0.88, 0.98)	0.93 (0.89, 0.97)
P for interaction	0.51	0.76	0.89	0.86	0.42
**Townsend Deprivation Index**					
Below median	0.96 (0.92, 1.01)	0.95 (0.91, 0.995)	1.004 (0.96, 1.05)	0.95 (0.91, 0.999)	0.95 (0.91, 0.995)
Above median	0.90 (0.86, 0.94)	0.89 (0.85, 0.93)	0.89 (0.85, 0.93)	0.90 (0.86, 0.94)	0.89 (0.85, 0.93)
P for interaction	0.02	0.01	0.002	0.05	0.02
**Obesity**					
Yes (BMI ≥30 kg/m^2)	0.97 (0.91, 1.04)	0.96 (0.90, 1.03)	0.97 (0.90, 1.03)	0.95 (0.89, 1.01)	0.95 (0.90, 1.02)
No (BMI <30 kg/m^2)	0.92 (0.89, 0.95)	0.91 (0.88, 0.94)	0.94 (0.90, 0.98)	0.92 (0.89, 0.96)	0.91 (0.88, 0.94)
P for interaction	0.25	0.15	0.75	0.66	0.22
**Smoking**					
Never	0.93 (0.88, 0.97)	0.92 (0.88, 0.97)	0.94 (0.90, 0.99)	0.91 (0.87, 0.95)	0.94 (0.89, 0.98)
Former or current	0.90 (0.87, 0.94)	0.89 (0.85, 0.93)	0.93 (0.89, 0.97)	0.91 (0.87, 0.95)	0.89 (0.85, 0.93)
P for interaction	0.26	0.07	0.67	0.69	0.04
**Energy intake**					
Below median	0.91 (0.86, 0.95)	0.90 (0.86, 0.94)	0.94 (0.89, 0.99)	0.92 (0.88, 0.97)	0.89 (0.85, 0.93)
Above median	0.95 (0.91, 0.99)	0.94 (0.90, 0.98)	0.95 (0.91, 0.99)	0.93 (0.89, 0.97)	0.95 (0.91, 0.99)
P for interaction	0.20	0.28	0.86	0.72	0.049

**Physical activity**					
Below median	0.93 (0.88, 0.99)	0.93 (0.88, 0.98)	0.95 (0.90, 1.01)	0.94 (0.89, 0.996)	0.94 (0.89, 0.99)
Above median	0.93 (0.89, 0.98)	0.93 (0.89, 0.97)	0.95 (0.91, 0.99)	0.91 (0.87, 0.96)	0.91 (0.88, 0.96)
P for interaction	0.999	0.82	0.83	0.34	0.41

Data are shown as HR (95% CI).

Adjusted for age (in years, continuous), sex (male/female), ethnicity (white/not-white), education (lower secondary, upper secondary, vocational, college or university, or others), TDI (in quintiles), assessment centers (22 categories), smoking (current, former, or never), physical activity (0-599, 600-1199, ≥1200 MET-mins/week, or unknown), BMI (<25.0, 25.0–29.9, ≥30 kg/m^2, or unknown), total energy intake (KCAL, continuous), baseline dyslipidemia (yes/no), hypertension (yes/no), diabetes (yes/no), longevity-PRS (in tertiles), top 10 principle genetic components (continuous), and genotype measurement batches (continuous); alcohol consumption (0, 0.1–5, 5.1–10, 10.1–15, 15.1–20, 20.1–30, >30 g/day) was additionally adjusted for hPDI, DASH, and DRRD.

Abbreviations: AHEI, alternate healthy eating index; AMED; alternate Mediterranean diet; BMI, body mass index; CI, confidence interval; DASH, dietary approaches to stop hypertension; DRRD, diabetes risk reduction diet; hPDI, healthful plant-based diet; HR, hazard ratio; MET, metabolic equivalent of task; PRS, polygenic risk score; SD, standard deviation; TDI, Townsend Deprivation Index.

Table S12. Hazard ratios (95% CI) of all-cause mortality by dietary scores quintiles when further adjusting for general health status, pack years of smoking, food groups not included in each dietary score, or when not adjusting for total energy intake

	Quintile of dietary score					*P* for trend	per SD increment
	Quintile 1	Quintile 2	Quintile 3	Quintile 4	Quintile 5		
**AHEI**							
Median score	45 (41, 48)	54 (52, 56)	61 (59, 62)	67 (65, 69)	76 (73, 81)		
Cases/person years	996/218,823	898/218,711	876/218,791	801/219,021	743/219,122		
Further adjusted for general health status	1.00 (ref.)	0.95 (0.87, 1.04)	0.90 (0.82, 0.99)	0.83 (0.75, 0.91)	0.83 (0.75, 0.92)	<.0001	0.93 (0.90, 0.97)
Further adjusted for pack years of smoking	1.00 (ref.)	0.91 (0.83, 0.99)	0.89 (0.81, 0.98)	0.84 (0.76, 0.92)	0.81 (0.73, 0.90)	<.0001	0.93 (0.90, 0.96)
Further adjusted for food groups not included in the dietary score*	1.00 (ref.)	0.90 (0.83, 0.99)	0.88 (0.80, 0.97)	0.83 (0.75, 0.91)	0.80 (0.73, 0.89)	<.0001	0.93 (0.90, 0.96)
Final model without total energy intake	1.00 (ref.)	0.90 (0.82, 0.99)	0.88 (0.80, 0.97)	0.83 (0.75, 0.91)	0.80 (0.72, 0.88)	<.0001	0.93 (0.90, 0.96)
**AMED**							
Median score	20 (18, 21)	24 (23, 25)	27 (26, 28)	30 (29, 31)	35 (34, 37)		
Cases/person years	942/227,792	879/209,644	858/218,227	869/229,324	766/209,481		
Further adjusted for general health status	1.00 (ref.)	0.97 (0.88, 1.06)	0.90 (0.82, 0.99)	0.85 (0.77, 0.93)	0.81 (0.74, 0.90)	<.0001	0.93 (0.90, 0.96)
Further adjusted for pack years of smoking	1.00 (ref.)	0.96 (0.87, 1.05)	0.89 (0.81, 0.98)	0.84 (0.77, 0.93)	0.81 (0.73, 0.90)	<.0001	0.92 (0.90, 0.95)
Further adjusted for food groups not included in the dietary score*	1.00 (ref.)	0.96 (0.87, 1.05)	0.89 (0.81, 0.98)	0.84 (0.76, 0.93)	0.81 (0.73, 0.89)	<.0001	0.92 (0.89, 0.95)
Final model without total energy intake	1.00 (ref.)	0.96 (0.87, 1.05)	0.89 (0.81, 0.98)	0.84 (0.77, 0.93)	0.81 (0.73, 0.89)	<.0001	0.92 (0.90, 0.95)
**hPDI**							

Median score	47 (45, 48)	52 (51, 53)	55 (54, 56)	58 (57, 59)	63 (62, 65)		
Cases/person years	864/200,289	1,014/236,866	852/209,446	872/236,034	712/211,832		
Further adjusted for general health status	1.00 (ref.)	0.98 (0.89, 1.07)	0.94 (0.85, 1.03)	0.87 (0.79, 0.96)	0.84 (0.75, 0.93)	0.0001	0.95 (0.92, 0.98)
Further adjusted for pack years of smoking	1.00 (ref.)	0.97 (0.88, 1.06)	0.93 (0.84, 1.02)	0.86 (0.78, 0.95)	0.83 (0.74, 0.92)	<.0001	0.95 (0.92, 0.98)
Further adjusted for food groups not included in the dietary score*	1.00 (ref.)	0.97 (0.88, 1.06)	0.92 (0.84, 1.02)	0.86 (0.78, 0.95)	0.82 (0.74, 0.91)	<.0001	0.94 (0.91, 0.98)
Final model without total energy intake	1.00 (ref.)	0.96 (0.88, 1.05)	0.92 (0.83, 1.01)	0.85 (0.77, 0.94)	0.81 (0.73, 0.90)	<.0001	0.94 (0.91, 0.97)
**DASH**							
Median score	16 (15, 18)	20 (19, 21)	22 (22, 23)	25 (24, 26)	29 (27, 30)		
Cases/person years	993/234,222	1,049/241,870	673/179,063	875/229,151	724/210,162		
Further adjusted for general health status	1.00 (ref.)	0.99 (0.91, 1.08)	0.86 (0.78, 0.96)	0.88 (0.80, 0.97)	0.82 (0.74, 0.91)	<.0001	0.93 (0.90, 0.96)
Further adjusted for pack years of smoking	1.00 (ref.)	0.99 (0.91, 1.08)	0.86 (0.78, 0.95)	0.88 (0.80, 0.97)	0.82 (0.74, 0.90)	<.0001	0.93 (0.90, 0.96)
Further adjusted for food groups not included in the dietary score*	1.00 (ref.)	0.99 (0.90, 1.08)	0.86 (0.77, 0.95)	0.87 (0.79, 0.96)	0.81 (0.73, 0.90)	<.0001	0.92 (0.89, 0.95)
Final model without total energy intake	1.00 (ref.)	0.98 (0.90, 1.07)	0.85 (0.77, 0.94)	0.87 (0.79, 0.95)	0.81 (0.73, 0.89)	<.0001	0.92 (0.89, 0.95)
**DRRD**							
Median score	18 (17, 20)	23 (22, 24)	25 (25, 26)	28 (27, 29)	33 (32, 35)		
Cases/person years	876/194,850	1,117/278,220	628/154,510	990/259,930	703/206,957		
Further adjusted for general health status	1.00 (ref.)	0.90 (0.82, 0.98)	0.90 (0.81, 0.999)	0.86 (0.79, 0.95)	0.77 (0.70, 0.85)	<.0001	0.92 (0.89, 0.95)
Further adjusted for pack years of smoking	1.00 (ref.)	0.89 (0.82, 0.98)	0.90 (0.81, 0.99)	0.86 (0.78, 0.94)	0.76 (0.69, 0.85)	<.0001	0.92 (0.89, 0.95)

Further adjusted for food groups not included in the dietary score*	1.00 (ref.)	0.90 (0.82, 0.98)	0.89 (0.81, 0.99)	0.86 (0.78, 0.94)	0.76 (0.68, 0.84)	<.0001	0.92 (0.89, 0.95)
Final model without total energy intake	1.00 (ref.)	0.89 (0.82, 0.98)	0.90 (0.81, 0.99)	0.86 (0.78, 0.94)	0.77 (0.69, 0.85)	<.0001	0.92 (0.89, 0.95)

Other covariates included in the model were: age (in years, continuous), sex (male/female), ethnicity (white/not-white), education (lower secondary, upper secondary, vocational, college or university, or others), TDI (in quintiles), assessment centers (22 categories), smoking (current, former, or never), physical activity (0-599, 600-1199, ≥1200 MET-mins/week, or unknown), BMI (<25.0, 25.0–29.9, ≥30 kg/m^2, or unknown), total energy intake (KCAL, continuous), baseline dyslipidemia (yes/no), hypertension (yes/no), diabetes (yes/no), longevity-PRS (in tertiles), top 10 genetic primary components (continuous), and genotype measurement batches (continuous); alcohol consumption (0, 0.1–5, 5.1–10, 10.1–15, 15.1–20, 20.1–30, >30 g/day) was adjusted for hPDI, DASH, and DRRD.

*In AHEI, further adjusted for food groups including refined grains, potatoes, poultry, eggs, dairy, butter, fruit juice, sweets and snacks, pizza, and mixed foods; in AMED, further adjusted for food groups including refined grains, potatoes, poultry, eggs, butter, sugar-sweetened beverages, fruit juice, sweets and snacks, pizza, and mixed food; in hPDI, further adjusted for food groups including mixed food; in DASH, further adjusted food groups including refined grains, potatoes, fish and other seafood, poultry, eggs, butter, high-fat dairy, sweets and snacks, pizza, and mixed food; in DDRD, further adjusted for food groups including refined grains, potatoes, poultry, eggs, dairy, butter, sweets and snacks, pizza, and mixed food.

Abbreviations: AHEI, alternate healthy eating index; AMED, alternate Mediterranean diet; BMI, body mass index; CI, confidence interval; DASH, dietary approaches to stop hypertension; DRRD, diabetes risk reduction diet; hPDI, healthful plant-based diet; MET, metabolic equivalent of task; PRS, polygenic risk score; SD, standard deviation; TDI, Townsend Deprivation Index.

Table S13. Hazard ratios (95% CI) of all-cause mortality by dietary scores quintiles in never smokers (n=60,629)

	Quintile of dietary score					*P* for trend	per SD increment
	Quintile 1	Quintile 2	Quintile 3	Quintile 4	Quintile 5		
**AHEI**							
Median score	46 (42, 49)	55 (52, 56)	61 (60, 63)	67 (66, 69)	76 (73, 81)		
No. of participants	12,125	12,126	12,126	12,126	12,126		
Cases/person years	406/128,671	392/128,575	406/128,453	377/128,400	361/128,479		
Model 1	1.00 (ref.)	0.86 (0.75, 0.99)	0.85 (0.74, 0.97)	0.77 (0.67, 0.89)	0.71 (0.62, 0.83)	<.0001	0.89 (0.85, 0.93)
Model 2	1.00 (ref.)	0.91 (0.79, 1.05)	0.92 (0.80, 1.06)	0.84 (0.73, 0.98)	0.80 (0.69, 0.93)	0.0013	0.93 (0.88, 0.97)
**AMED**							
Median score	20 (18, 21)	24 (23, 25)	27 (26, 28)	30 (29, 31)	35 (33, 37)		
No. of participants	12,148	11,536	12,188	12,729	12,028		
Cases/person years	391/129,136	376/122,333	412/129073	379/134,753	384/127,282		
Model 1	1.00 (ref.)	0.90 (0.78, 1.03)	0.88 (0.77, 1.01)	0.74 (0.64, 0.85)	0.73 (0.64, 0.85)	<.0001	0.90 (0.86, 0.94)
Model 2	1.00 (ref.)	0.93 (0.81, 1.07)	0.93 (0.81, 1.07)	0.78 (0.68, 0.90)	0.79 (0.68, 0.92)	0.0009	0.92 (0.88, 0.96)
**hPDI**							
Median score	47 (45, 48)	52 (51, 53)	55 (54, 56)	58 (57, 59)	63 (62, 66)		
No. of participants	12,148	11,536	12,188	12,729	12,028		
Cases/person years	381/118,876	436/139,326	388/122,697	404/139,212	333/122,467		
Model 1	1.00 (ref.)	0.88 (0.77, 1.01)	0.84 (0.73, 0.97)	0.77 (0.67, 0.89)	0.72 (0.62, 0.84)	<.0001	0.91 (0.86, 0.95)
Model 2	1.00 (ref.)	0.93 (0.81, 1.07)	0.90 (0.78, 1.05)	0.85 (0.73, 0.98)	0.80 (0.69, 0.94)	0.0017	0.94 (0.90, 0.99)
**DASH**							
Median score	16 (15, 18)	20 (19, 21)	22 (22, 23)	25 (24, 26)	29 (27, 30)		
No. of participants	12,900	13,319	9,899	12,758	11,753		
Cases/person years	408/136,888	474/141,051	305/104,942	413/135,178	342/124,519		
Model 1	1.00 (ref.)	0.96 (0.84, 1.09)	0.80 (0.69, 0.93)	0.81 (0.70, 0.93)	0.71 (0.61, 0.82)	<.0001	0.88 (0.84, 0.93)
Model 2	1.00 (ref.)	1.01 (0.88, 1.15)	0.85 (0.73, 0.99)	0.87 (0.76, 1.01)	0.77 (0.66, 0.90)	0.0001	0.91 (0.87, 0.96)
**DRRD**							
Median score	18 (17, 20)	22 (21, 23)	25 (24, 26)	28 (27, 29)	33 (32, 35)		

No. of participants	10,963	11,357	12,837	14,235	11,237		
Cases/person years	353/116,160	357/120,455	435/135,931	462/150,833	335/119,199		
Model 1	1.00 (ref.)	0.90 (0.77, 1.04)	0.93 (0.81, 1.07)	0.89 (0.77, 1.02)	0.78 (0.67, 0.91)	0.0006	0.92 (0.88, 0.96)
Model 2	1.00 (ref.)	0.92 (0.79, 1.07)	0.96 (0.84, 1.11)	0.92 (0.79, 1.05)	0.82 (0.70, 0.96)	0.0055	0.93 (0.89, 0.98)

Model 1 adjusted for age (in years, continuous) and sex (male/female);
Model 2 adjusted for covariates in model 1 plus ethnicity (white/not-white), education (lower secondary, upper secondary, vocational, college or university, or others), TDI (in quintiles), assessment centers (22 categories), physical activity (0-599, 600-1199, ≥1200 MET-mins/week, or unknown), BMI (<25.0, 25.0–29.9, ≥30 kg/m^2, or unknown), total energy intake (KCAL, continuous), baseline dyslipidemia (yes/no), hypertension (yes/no), diabetes (yes/no), longevity-PRS (in tertiles), top 10 genetic primary components (continuous), and genotype measurement batches (continuous); alcohol consumption (0, 0.1–5, 5.1–10, 10.1–15, 15.1–20, 20.1–30, >30 g/day) was adjusted for hPDI, DASH, and DRRD.
Abbreviations: AHEI, alternate healthy eating index; AMED; alternate Mediterranean diet; BMI, body mass index; CI, confidence interval; DASH, dietary approaches to stop hypertension; DRRD, diabetes risk reduction diet; hPDI, healthful plant-based diet; MET, metabolic equivalent of task; PRS, polygenic risk score; SD, standard deviation; TDI, Townsend Deprivation Index.

Table S14. Hazard ratios (95% CI) of all-cause mortality by dietary scores quintiles among participants free of diabetes (n=99,474)

	Quintile of dietary score					*P* for trend	per SD increment
	Quintile 1	Quintile 2	Quintile 3	Quintile 4	Quintile 5		
**AHEI**							
Median score	45 (41, 48)	54 (52, 56)	61 (59, 62)	67 (65, 69)	76 (73, 81)		
No. of participants	19,894	19,895	19,895	19,895	19,895		
Cases/person years	885/210,263	820/210,021	773/210,260	713/210,446	691/210,351		
Model 1	1.00 (ref.)	0.85 (0.77, 0.94)	0.77 (0.70, 0.85)	0.70 (0.64, 0.78)	0.68 (0.62, 0.76)	<.0001	0.87 (0.84, 0.90)
Model 2	1.00 (ref.)	0.89 (0.81, 0.98)	0.82 (0.75, 0.91)	0.76 (0.69, 0.84)	0.76 (0.68, 0.84)	<.0001	0.90 (0.87, 0.94)
**AMED**							
Median score	20 (18, 21)	24 (23, 25)	27 (26, 28)	30 (29, 31)	35 (34, 37)		
No. of participants	20,757	19,032	19,895	20,883	18,907		
Cases/person years	851/219,797	776/201,117	794/210,124	774/220,520	687/199,783		
Model 1	1.00 (ref.)	0.89 (0.81, 0.98)	0.82 (0.74, 0.90)	0.73 (0.66, 0.81)	0.68 (0.62, 0.76)	<.0001	0.87 (0.84, 0.90)
Model 2	1.00 (ref.)	0.92 (0.84, 1.02)	0.87 (0.79, 0.96)	0.79 (0.71, 0.87)	0.75 (0.68, 0.83)	<.0001	0.90 (0.87, 0.93)
**hPDI**							
Median score	47 (45, 48)	52 (51, 53)	55 (54, 56)	58 (57, 59)	63 (62, 65)		
No. of participants	18,518	21,822	19,094	21,279	18,761		
Cases/person years	789/195,670	907/230,096	773/201,748	781/225,068	632/198,759		
Model 1	1.00 (ref.)	0.89 (0.81, 0.98)	0.84 (0.76, 0.93)	0.77 (0.69, 0.85)	0.72 (0.65, 0.80)	<.0001	0.90 (0.87, 0.93)
Model 2	1.00 (ref.)	0.92 (0.84, 1.01)	0.89 (0.80, 0.98)	0.82 (0.74, 0.91)	0.78 (0.70, 0.87)	<.0001	0.93 (0.90, 0.97)
**DASH**							
Median score	16 (15, 18)	20 (19, 21)	22 (22, 23)	25 (24, 26)	29 (27, 30)		
No. of participants	21,190	22,036	16,260	20,823	19,165		
Cases/person years	889/223,942	949/232,506	595/172,018	777/220,113	672/202,763		
Model 1	1.00 (ref.)	0.91 (0.83, 0.99)	0.75 (0.67, 0.83)	0.74 (0.68, 0.82)	0.70 (0.63, 0.78)	<.0001	0.87 (0.85, 0.90)
Model 2	1.00 (ref.)	0.95 (0.87, 1.05)	0.79 (0.71, 0.88)	0.80 (0.73, 0.89)	0.77 (0.69, 0.85)	<.0001	0.90 (0.87, 0.94)
**DRRD**							

Median score	18 (17, 20)	23 (22, 24)	25 (25, 26)	28 (27, 29)	33 (32, 35)		
No. of participants	17,691	25,273	14,026	23,685	18,799		
Cases/person years	782/186,632	997/267,015	568/148,208	898/250,374	637/199,113		
Model 1	1.00 (ref.)	0.83 (0.76, 0.91)	0.84 (0.75, 0.94)	0.78 (0.71, 0.86)	0.68 (0.61, 0.76)	<.0001	0.89 (0.86, 0.92)
Model 2	1.00 (ref.)	0.86 (0.79, 0.95)	0.88 (0.79, 0.98)	0.82 (0.75, 0.91)	0.73 (0.66, 0.81)	<.0001	0.91 (0.88, 0.94)

Model 1 adjusted for age (in years, continuous) and sex (male/female);
Model 2 adjusted for covariates in model 1 plus ethnicity (white/not-white), education (lower secondary, upper secondary, vocational, college or university, or others), TDI (in quintiles), assessment centers (22 categories), smoking (current, former, or never), physical activity (0-599, 600-1199, ≥1200 MET-mins/week, or unknown), BMI (<25.0, 25.0–29.9, ≥30 kg/m^2, or unknown), total energy intake (KCAL, continuous), hypertension (yes/no), longevity-PRS (in tertiles), top 10 genetic primary components (continuous), and genotype measurement batches (continuous); alcohol consumption (0, 0.1–5, 5.1–10, 10.1–15, 15.1–20, 20.1–30, >30 g/day) was adjusted for hPDI, DASH, and DRRD.
Abbreviations: AHEI, alternate healthy eating index; AMED; alternate Mediterranean diet; BMI, body mass index; CI, confidence interval; DASH, dietary approaches to stop hypertension; DRRD, diabetes risk reduction diet; hPDI, healthful plant-based diet; MET, metabolic equivalent of task; PRS, polygenic risk score; SD, standard deviation; TDI, Townsend Deprivation Index.

Table S15. Hazard ratios (95% CI) of all-cause mortality by dietary scores quintiles after excluding participants reporting any of the five dietary assessments as not typical (n=59,478)

	Quintile of dietary score					*P* for trend	per SD increment
	Quintile 1	Quintile 2	Quintile 3	Quintile 4	Quintile 5		
**AHEI**							
Median score	47 (43, 50)	55 (54, 57)	62 (60, 63)	68 (66, 70)	77 (74, 82)		
No. of participants	11,895	11,896	11,896	11,896	11,895		
Cases/person years	648/125,663	576/125,603	545/125,569	502/125,629	440/125,939		
Model 1	1.00 (ref.)	0.83 (0.74, 0.93)	0.77 (0.68, 0.86)	0.71 (0.63, 0.80)	0.64 (0.56, 0.72)	<.0001	0.85 (0.82, 0.89)
Model 2	1.00 (ref.)	0.88 (0.79, 0.99)	0.84 (0.75, 0.94)	0.79 (0.70, 0.89)	0.73 (0.64, 0.83)	<.0001	0.90 (0.86, 0.93)
**AMED**							
Median score	20 (18, 21)	24 (23, 25)	27 (26, 28)	30 (29, 31)	35 (34, 37)		
No. of participants	11,894	10,804	12,010	12,876	11,894		
Cases/person years	591/125,890	527/114,061	536/126940	545/135,888	512/125,625		
Model 1	1.00 (ref.)	0.90 (0.80, 1.02)	0.79 (0.70, 0.89)	0.73 (0.65, 0.82)	0.70 (0.62, 0.79)	<.0001	0.87 (0.84, 0.91)
Model 2	1.00 (ref.)	0.93 (0.83, 1.05)	0.84 (0.75, 0.95)	0.78 (0.69, 0.88)	0.78 (0.69, 0.89)	<.0001	0.90 (0.87, 0.94)
**hPDI**							
Median score	48 (45, 49)	52 (51, 53)	55 (54, 56)	58 (57, 59)	63 (62, 66)		
No. of participants	12,647	9,784	11,403	13,282	12,362		
Cases/person years	665/133,420	458/103,266	549/120,319	585/140,407	454/130,991		
Model 1	1.00 (ref.)	0.84 (0.75, 0.95)	0.86 (0.77, 0.96)	0.79 (0.71, 0.89)	0.69 (0.62, 0.78)	<.0001	0.89 (0.86, 0.93)
Model 2	1.00 (ref.)	0.87 (0.77, 0.98)	0.93 (0.83, 1.04)	0.86 (0.77, 0.97)	0.77 (0.68, 0.88)	0.0001	0.93 (0.89, 0.97)
**DASH**							
Median score	16 (15, 18)	20 (19, 21)	22 (22, 23)	25 (24, 26)	29 (27, 30)		
No. of participants	12,236	13,031	9,782	12,596	11,833		
Cases/person years	629/129,372	651/137,432	444/103,240	535/133,187	452/125,172		
Model 1	1.00 (ref.)	0.89 (0.79, 0.99)	0.79 (0.70, 0.89)	0.73 (0.65, 0.83)	0.68 (0.60, 0.76)	<.0001	0.86 (0.83, 0.90)
Model 2	1.00 (ref.)	0.94 (0.84, 1.05)	0.85 (0.75, 0.96)	0.80 (0.71, 0.90)	0.76 (0.67, 0.86)	<.0001	0.90 (0.87, 0.94)
**DRRD**							

Median score	18 (17, 20)	23 (22, 24)	25 (25, 26)	28 (27, 29)	33 (32, 35)		
No. of participants	10,783	14,972	8,289	13,994	11,440		
Cases/person years	585/113,561	703/158,162	395/87,520	593/148,043	435/121,118		
Model 1	1.00 (ref.)	0.81 (0.73, 0.91)	0.82 (0.72, 0.94)	0.73 (0.65, 0.82)	0.66 (0.58, 0.75)	<.0001	0.87 (0.84, 0.91)
Model 2	1.00 (ref.)	0.85 (0.76, 0.95)	0.86 (0.75, 0.98)	0.78 (0.69, 0.88)	0.72 (0.63, 0.82)	<.0001	0.90 (0.86, 0.93)

Model 1 adjusted for age (in years, continuous) and sex (male/female);
Model 2 adjusted for covariates in model 1 plus ethnicity (white/not-white), education (lower secondary, upper secondary, vocational, college or university, or others), TDI (in quintiles), assessment centers (22 categories), smoking (current, former, or never), physical activity (0-599, 600-1199, ≥1200 MET-mins/week, or unknown), BMI (<25.0, 25.0–29.9, ≥30 kg/m^2, or unknown), total energy intake (KCAL, continuous), baseline dyslipidemia (yes/no), hypertension (yes/no), diabetes (yes/no), longevity-PRS (in tertiles), top 10 genetic primary components (continuous), and genotype measurement batches (continuous); alcohol consumption (0, 0.1–5, 5.1–10, 10.1–15, 15.1–20, 20.1–30, >30 g/day) was adjusted for hPDI, DASH, and DRRD.
Abbreviations: AHEI, alternate healthy eating index; AMED; alternate Mediterranean diet; BMI, body mass index; CI, confidence interval; DASH, dietary approaches to stop hypertension; DRRD, diabetes risk reduction diet; hPDI, healthful plant-based diet; MET, metabolic equivalent of task; PRS, polygenic risk score; SD, standard deviation; TDI, Townsend Deprivation Index.

Table S16. Hazard ratios (95% CI) of all-cause mortality by dietary scores quintiles among participants with three or more dietary assessments (n=63,472)

	Quintile of dietary score					*P* for trend	per SD increment
	Quintile 1	Quintile 2	Quintile 3	Quintile 4	Quintile 5		
**AHEI**							
Median score	46 (42, 49)	55 (53, 56)	61 (59, 63)	67 (66, 69)	76 (73, 81)		
No. of participants	12,694	12,695	12,694	12,695	12,694		
Cases/person years	592/132,561	557/132,478	516/132,692	462/132,912	444/133,000		
Model 1	1.00 (ref.)	0.89 (0.79, 0.995)	0.79 (0.70, 0.89)	0.72 (0.63, 0.81)	0.69 (0.61, 0.78)	<.0001	0.86 (0.83, 0.90)
Model 2	1.00 (ref.)	0.94 (0.84, 1.06)	0.87 (0.77, 0.98)	0.80 (0.71, 0.91)	0.81 (0.71, 0.92)	<.0001	0.91 (0.87, 0.95)
**AMED**							
Median score	20 (18, 21)	24 (23, 25)	27 (26, 28)	30 (29, 31)	35 (34, 37)		
No. of participants	12,697	11,662	12,582	13,837	12,694		
Cases/person years	591/132,544	486/121,841	502/131,523	516/144,795	476/132,940		
Model 1	1.00 (ref.)	0.82 (0.73, 0.92)	0.75 (0.67, 0.84)	0.67 (0.60, 0.76)	0.65 (0.57, 0.73)	<.0001	0.85 (0.82, 0.88)
Model 2	1.00 (ref.)	0.87 (0.77, 0.98)	0.82 (0.73, 0.93)	0.75 (0.66, 0.84)	0.74 (0.66, 0.84)	<.0001	0.89 (0.86, 0.93)
**hPDI**							
Median score	47 (45, 48)	52 (51, 53)	55 (54, 56)	58 (57, 59)	63 (62, 66)		
No. of participants	11,790	13,846	12,131	13,516	12,189		
Cases/person years	546/123,076	589/144,611	515/126,746	506/141,477	415/127,732		
Model 1	1.00 (ref.)	0.83 (0.74, 0.93)	0.82 (0.73, 0.93)	0.73 (0.65, 0.83)	0.68 (0.60, 0.78)	<.0001	0.88 (0.85, 0.92)
Model 2	1.00 (ref.)	0.88 (0.78, 0.995)	0.91 (0.80, 1.03)	0.83 (0.73, 0.94)	0.80 (0.70, 0.92)	0.0012	0.94 (0.90, 0.98)
**DASH**							
Median score	16 (15, 18)	20 (19, 21)	22 (22, 23)	25 (24, 26)	29 (27, 30)		
No. of participants	13,501	13,684	10,232	13,248	12,807		
Cases/person years	602/140,998	566/143,094	428/106,861	537/138,513	438/134,177		
Model 1	1.00 (ref.)	0.83 (0.74, 0.93)	0.82 (0.72, 0.93)	0.78 (0.69, 0.88)	0.66 (0.59, 0.75)	<.0001	0.86 (0.83, 0.90)
Model 2	1.00 (ref.)	0.88 (0.78, 0.99)	0.89 (0.79, 1.01)	0.86 (0.76, 0.97)	0.76 (0.66, 0.86)	<.0001	0.91 (0.87, 0.95)
**DRRD**							

Median score	18 (17, 20)	23 (22, 24)	25 (25, 26)	28 (27, 29)	33 (32, 35)		
No. of participants	11,205	15,910	8,898	14,951	12,508		
Cases/person years	516/116,976	669/166,196	358/93,112	584/156,338	444/131,022		
Model 1	1.00 (ref.)	0.85 (0.76, 0.96)	0.81 (0.71, 0.93)	0.78 (0.69, 0.88)	0.70 (0.62, 0.80)	<.0001	0.88 (0.85, 0.92)
Model 2	1.00 (ref.)	0.90 (0.80, 1.01)	0.86 (0.75, 0.99)	0.84 (0.75, 0.95)	0.77 (0.68, 0.88)	<.0001	0.91 (0.88, 0.95)

Model 1 adjusted for age (in years, continuous) and sex (male/female);
Model 2 adjusted for covariates in model 1 plus ethnicity (white/not-white), education (lower secondary, upper secondary, vocational, college or university, or others), TDI (in quintiles), assessment centers (22 categories), smoking (current, former, or never), physical activity (0-599, 600-1199, ≥1200 MET-mins/week, or unknown), BMI (<25.0, 25.0–29.9, ≥30 kg/m^2, or unknown), total energy intake (KCAL, continuous), baseline dyslipidemia (yes/no), hypertension (yes/no), diabetes (yes/no), longevity-PRS (in tertiles), top 10 genetic primary components (continuous), and genotype measurement batches (continuous); alcohol consumption (0, 0.1–5, 5.1–10, 10.1–15, 15.1–20, 20.1–30, >30 g/day) was adjusted for hPDI, DASH, and DRRD.
Abbreviations: AHEI, alternate healthy eating index; AMED; alternate Mediterranean diet; BMI, body mass index; CI, confidence interval; DASH, dietary approaches to stop hypertension; DRRD, diabetes risk reduction diet; hPDI, healthful plant-based diet; MET, metabolic equivalent of task; PRS, polygenic risk score; SD, standard deviation; TDI, Townsend Deprivation Index.

Table S17. Hazard ratios (95% CI) of all-cause mortality by dietary scores quintiles after excluding participants with less than two years of follow-up (n=103,205)

	Quintile of dietary score					*P* for trend	Per SD increment
	Quintile 1	Quintile 2	Quintile 3	Quintile 4	Quintile 5		
**AHEI**							
Median score	45 (41, 48)	54 (52, 56)	61 (59, 62)	67 (65, 69)	76 (73, 81)		
Cases/person-years	917/218,796	822/218,733	812/218,665	739/218,789	677/219,007		
Model 1	1.00 (ref.)	0.83 (0.75, 0.91)	0.79 (0.72, 0.87)	0.71 (0.65, 0.79)	0.66 (0.59, 0.73)	<.0001	0.86 (0.83, 0.89)
Model 2	1.00 (ref.)	0.90 (0.82, 0.99)	0.89 (0.81, 0.98)	0.83 (0.75, 0.92)	0.80 (0.72, 0.89)	<.0001	0.93 (0.89, 0.96)
**AMED**							
Median score	20 (18, 21)	24 (23, 25)	27 (26, 28)	30 (29, 31)	35 (34, 37)		
Cases/person-years	884/227,710	793/209,533	791/218,134	792/229,210	707/209,403		
Model 1	1.00 (ref.)	0.86 (0.78, 0.95)	0.78 (0.71, 0.86)	0.71 (0.65, 0.78)	0.66 (0.60, 0.73)	<.0001	0.86 (0.83, 0.89)
Model 2	1.00 (ref.)	0.92 (0.83, 1.01)	0.87 (0.79, 0.95)	0.81 (0.73, 0.89)	0.78 (0.70, 0.86)	<.0001	0.92 (0.89, 0.95)
**hPDI**							
Median score	47 (45, 48)	52 (51, 53)	55 (54, 56)	58 (57, 59)	63 (62, 65)		
Cases/person-years	793/200,189	930/236,768	797/209,368	801/235,924	646/211,740		
Model 1	1.00 (ref.)	0.91 (0.83, 1.001)	0.86 (0.78, 0.95)	0.77 (0.70, 0.85)	0.71 (0.64, 0.79)	<.0001	0.90 (0.87, 0.93)
Model 2	1.00 (ref.)	0.97 (0.88, 1.07)	0.95 (0.85, 1.05)	0.87 (0.78, 0.96)	0.82 (0.74, 0.92)	<.0001	0.95 (0.91, 0.98)
**DASH**							
Median score	16 (15, 18)	20 (19, 21)	22 (22, 23)	25 (24, 26)	29 (27, 30)		
Cases/person-years	913/234,098	963/241,737	615/178,992	811/229,071	665/210,092		
Model 1	1.00 (ref.)	0.90 (0.83, 0.99)	0.76 (0.69, 0.84)	0.77 (0.70, 0.85)	0.69 (0.62, 0.77)	<.0001	0.87 (0.84, 0.90)
Model 2	1.00 (ref.)	0.99 (0.90, 1.08)	0.85 (0.77, 0.95)	0.88 (0.80, 0.97)	0.81 (0.73, 0.90)	<.0001	0.92 (0.89, 0.96)
**DRRD**							
Median score	18 (17, 20)	23 (22, 24)	25 (25, 26)	28 (27, 29)	33 (32, 35)		
Cases/person-years	805/194,829	1021/278,103	580/154,451	920/259,825	641/206,782		
Model 1	1.00 (ref.)	0.84 (0.76, 0.91)	0.83 (0.75, 0.92)	0.78 (0.71, 0.85)	0.68 (0.62, 0.75)	<.0001	0.89 (0.86, 0.91)
Model 2	1.00 (ref.)	0.88 (0.81, 0.97)	0.89 (0.80, 0.99)	0.86 (0.78, 0.95)	0.75 (0.67, 0.84)	<.0001	0.92 (0.89, 0.95)

Model 1 adjusted for age (in years, continuous) and sex (male/female);
Model 2 adjusted for covariates in model 1 plus ethnicity (white/not-white), education (lower secondary, upper secondary, vocational, college or university, or others), TDI (in quintiles), assessment centers (22 categories), smoking (current, former, or never), physical activity (0-599, 600-1199, ≥1200 MET-mins/week, or unknown), BMI (<25.0, 25.0–29.9, ≥30 kg/m^2, or unknown), total energy intake (KCAL, continuous), baseline dyslipidemia (yes/no), hypertension (yes/no), diabetes (yes/no), longevity-PRS (in tertiles), top 10 genetic primary components (continuous), and genotype measurement batches (continuous); alcohol consumption (0, 0.1–5, 5.1–10, 10.1–15, 15.1–20, 20.1–30, >30 g/day) was adjusted for hPDI, DASH, and DRRD.
Abbreviations: AHEI, alternate healthy eating index; AMED; alternate Mediterranean diet; BMI, body mass index; CI, confidence interval; DASH, dietary approaches to stop hypertension; DRRD, diabetes risk reduction diet; hPDI, healthful plant-based diet; MET, metabolic equivalent of task; PRS, polygenic risk score; SD, standard deviation; TDI, Townsend Deprivation Index.

Table S18. Associations of AHEI and AMED with all-cause mortality after removing alcohol from score component

	Cases/ person-years	Model 1	Model 2
AHEI			
Quintile 1	956/218,691	1.00 (reference)	1.00 (reference)
Quintile 2	938/218,580	0.91 (0.83, 0.99)	0.98 (0.89, 1.07)
Quintile 3	863/218,798	0.82 (0.74, 0.90)	0.90 (0.82, 0.99)
Quintile 4	818/219,068	0.77 (0.70, 0.85)	0.87 (0.79, 0.96)
Quintile 5	739/219,331	0.71 (0.64, 0.78)	0.82 (0.74, 0.90)
P-trend		<.0001	<.0001
Per SD increment		0.88 (0.85, 0.91)	0.93 (0.90, 0.96)
AMED			
Quintile 1	811/194,286	1.00 (reference)	1.00 (reference)
Quintile 2	860/209,870	0.86 (0.78, 0.95)	0.92 (0.84, 1.01)
Quintile 3	931/238,562	0.78 (0.71, 0.86)	0.86 (0.78, 0.94)
Quintile 4	810/209,260	0.75 (0.68, 0.83)	0.84 (0.76, 0.93)
Quintile 5	902/242,489	0.70 (0.63, 0.77)	0.80 (0.72, 0.88)
P-trend		<.0001	<.0001
Per SD increment		0.88 (0.85, 0.91)	0.92 (0.89, 0.95)

Data are shown as HR (95% CI).
Adjusted for age (in years, continuous), sex (male/female), ethnicity (white/not-white), education (lower secondary, upper secondary, vocational, college or university, or others), TDI (in quintiles), assessment centers (22 categories), smoking (current, former, or never), physical activity (0-599, 600-1199, ≥1200 MET-mins/week, or unknown), BMI (<25.0, 25.0–29.9, ≥30 kg/m^2, or unknown), total energy intake (KCAL, continuous), alcohol consumption (0, 0.1–5, 5.1–10, 10.1–15, 15.1–20, 20.1–30, >30 g/day), baseline dyslipidemia (yes/no), hypertension (yes/no), diabetes (yes/no), longevity-PRS (in tertiles), top 10 principle genetic components (continuous), and genotype measurement batches (continuous).

Abbreviations: AHEI, alternate healthy eating index; AMED; alternate Mediterranean diet; BMI, body mass index; CI, confidence interval; HR, hazard ratio; MET, metabolic equivalent of task; PRS, polygenic risk score; SD, standard deviation; TDI, Townsend Deprivation Index

Table S19. Hazard ratio (95% CI) of cause-specific mortality associated with the alternate healthy eating index using a Fine-Gray model

	Quintile of AHEI					*P* for trend	per SD increment
	Quintile 1	Quintile 2	Quintile 3	Quintile 4	Quintile 5		
Median score	45 (41, 48)	54 (52, 56)	61 (59, 62)	67 (65, 69)	76 (73, 81)		
No. of participants	20,729	20,730	20,730	20,730	20,730		
Person-years	218,823	218,711	218,791	219,021	219,122		
CVD mortality							
Cases	172	156	160	151	126		
Model 1	1.00 (ref.)	0.86 (0.69, 1.07)	0.87 (0.70, 1.08)	0.83 (0.67, 1.04)	0.72 (0.57, 0.91)	0.0084	0.90 (0.83, 0.96)
Model 2	1.00 (ref.)	0.95 (0.77, 1.19)	1.001 (0.80, 1.25)	0.997 (0.79, 1.25)	0.91 (0.71, 1.16)	0.58	0.97 (0.90, 1.05)
Cancer mortality							
Cases	544	480	459	443	426		
Model 1	1.00 (ref.)	0.81 (0.72, 0.92)	0.74 (0.66, 0.84)	0.71 (0.63, 0.81)	0.68 (0.59, 0.77)	<.0001	0.87 (0.83, 0.90)
Model 2	1.00 (ref.)	0.88 (0.78, 0.997)	0.84 (0.74, 0.96)	0.83 (0.73, 0.95)	0.83 (0.72, 0.95)	0.0043	0.93 (0.89, 0.98)
Neurodegenerative mortality							
Cases	60	68	63	58	63		
Model 1	1.00 (ref.)	1.03 (0.72, 1.45)	0.91 (0.63, 1.30)	0.83 (0.57, 1.20)	0.90 (0.62, 1.30)	0.35	0.96 (0.85, 1.09)
Model 2	1.00 (ref.)	1.002 (0.70, 1.43)	0.90 (0.62, 1.30)	0.80 (0.54, 1.17)	0.87 (0.59, 1.29)	0.29	0.95 (0.83, 1.09)
Respiratory mortality							
Cases	65	49	54	40	26		
Model 1	1.00 (ref.)	0.68 (0.47, 0.996)	0.72 (0.50, 1.05)	0.54 (0.36, 0.81)	0.36 (0.23, 0.56)	<.0001	0.72 (0.63, 0.83)
Model 2	1.00 (ref.)	0.78 (0.53, 1.13)	0.85 (0.59, 1.22)	0.65 (0.43, 0.98)	0.46 (0.28, 0.73)	0.0007	0.80 (0.70, 0.91)
Other mortality							
Cases	155	145	140	109	102		
Model 1	1.00 (ref.)	0.88 (0.71, 1.11)	0.83 (0.66, 1.05)	0.65 (0.51, 0.84)	0.62 (0.48, 0.81)	<.0001	0.85 (0.78, 0.92)
Model 2	1.00 (ref.)	0.95 (0.76, 1.20)	0.93 (0.74, 1.18)	0.76 (0.58, 0.98)	0.75 (0.57, 0.98)	0.011	0.91 (0.84, 0.99)

Model 1 adjusted for age (in years, continuous) and sex (male/female);

Model 2 adjusted for covariates in model 1 plus ethnicity (white/not-white), education (lower secondary, upper secondary, vocational, college or university, or others), TDI (in quintiles), assessment centers (22 categories), smoking (current, former, or never), physical activity (0-599, 600-1199, ≥1200 MET-mins/week, or unknown), BMI (<25.0, 25.0–29.9, ≥30 kg/m^2, or unknown), total energy intake (KCAL, continuous), baseline dyslipidemia (yes/no), hypertension (yes/no), diabetes (yes/no), longevity-PRS (in tertiles), top 10 genetic primary components (continuous), and genotype measurement batches (continuous).
Abbreviations: AHEI, alternate healthy eating index; BMI, body mass index; CI, confidence interval; MET, metabolic equivalent of task; PRS, polygenic risk score; SD, standard deviation; TDI, Townsend Deprivation Index.

Table S20. Hazard ratio (95% CI) of cause-specific mortality associated with the alternate Mediterranean diet using a Fine-Gray model

	Quintile of AMED					*P* for trend	per SD increment
	Quintile 1	Quintile 2	Quintile 3	Quintile 4	Quintile 5		
Median score	20 (18, 21)	24 (23, 25)	27 (26, 28)	30 (29, 31)	35 (34, 37)		
No. of participants	21,530	19,865	20,674	21,741	19,839		
Person-years	227,792	209,644	218,227	229,324	209,481		
CVD mortality							
Cases	150	146	149	176	144		
Model 1	1.00 (ref.)	0.91 (0.72, 1.14)	0.83 (0.66, 1.04)	0.89 (0.72, 1.11)	0.75 (0.60, 0.95)	0.024	0.92 (0.85, 0.99)
Model 2	1.00 (ref.)	0.98 (0.78, 1.23)	0.94 (0.75, 1.19)	1.04 (0.83, 1.30)	0.93 (0.73, 1.19)	0.76	0.99 (0.91, 1.07)
Cancer mortality							
Cases	518	486	479	455	414		
Model 1	1.00 (ref.)	0.92 (0.81, 1.04)	0.83 (0.74, 0.94)	0.73 (0.64, 0.82)	0.69 (0.61, 0.79)	<.0001	0.87 (0.83, 0.91)
Model 2	1.00 (ref.)	0.98 (0.86, 1.10)	0.92 (0.81, 1.04)	0.82 (0.72, 0.93)	0.80 (0.70, 0.92)	<.0001	0.92 (0.88, 0.96)
Neurodegenerative mortality							
Cases	54	53	68	71	66		
Model 1	1.00 (ref.)	0.93 (0.64, 1.36)	1.08 (0.76, 1.55)	1.03 (0.73, 1.46)	0.997 (0.70, 1.42)	0.87	1.002 (0.89, 1.13)
Model 2	1.00 (ref.)	0.94 (0.64, 1.36)	1.09 (0.76, 1.56)	1.02 (0.72, 1.46)	1.001 (0.69, 1.45)	0.87	1.002 (0.89, 1.13)
Respiratory mortality							
Cases	70	50	41	38	35		
Model 1	1.00 (ref.)	0.65 (0.45, 0.93)	0.47 (0.32, 0.69)	0.39 (0.27, 0.58)	0.37 (0.25, 0.56)	<.0001	0.67 (0.57, 0.77)
Model 2	1.00 (ref.)	0.71 (0.49, 1.02)	0.55 (0.37, 0.80)	0.47 (0.32, 0.71)	0.47 (0.31, 0.71)	<.0001	0.74 (0.64, 0.85)
Other mortality							
Cases	150	144	121	129	107		
Model 1	1.00 (ref.)	0.92 (0.73, 1.16)	0.70 (0.55, 0.89)	0.68 (0.54, 0.86)	0.59 (0.46, 0.75)	<.0001	0.82 (0.76, 0.89)
Model 2	1.00 (ref.)	1.01 (0.80, 1.27)	0.81 (0.64, 1.04)	0.82 (0.64, 1.04)	0.76 (0.58, 0.98)	0.011	0.90 (0.83, 0.98)

Model 1 adjusted for age (in years, continuous) and sex (male/female);

Model 2 adjusted for covariates in model 1 plus ethnicity (white/not-white), education (lower secondary, upper secondary, vocational, college or university, or others), TDI (in quintiles), assessment centers (22 categories), smoking (current, former, or never), physical activity (0-599, 600-1199, ≥1200 MET-mins/week, or unknown), BMI (<25.0, 25.0–29.9, ≥30 kg/m^2, or unknown), total energy intake (KCAL, continuous), baseline dyslipidemia (yes/no), hypertension (yes/no), diabetes (yes/no), longevity-PRS (in tertiles), top 10 genetic primary components (continuous), and genotype measurement batches (continuous).
Abbreviations: AMED; alternate Mediterranean diet; BMI, body mass index; CI, confidence interval; MET, metabolic equivalent of task; PRS, polygenic risk score; SD, standard deviation; TDI, Townsend Deprivation Index.

Table S21. Hazard ratio (95% CI) of cause-specific mortality associated with a healthful plant-based diet using a Fine-Gray model

	Quintile of hPDI					*P* for trend	per SD increment
	Quintile 1	Quintile 2	Quintile 3	Quintile 4	Quintile 5		
Median score	47 (45, 48)	52 (51, 53)	55 (54, 56)	58 (57, 59)	63 (62, 65)		
No. of participants	18,984	22,484	19,840	22,331	20,010		
Person-years	200,289	236,866	209,446	236,034	211,832		
CVD mortality							
Cases	157	195	126	157	130		
Model 1	1.00 (ref.)	0.98 (0.79, 1.21)	0.71 (0.56, 0.90)	0.81 (0.65, 1.02)	0.79 (0.63, 1.001)	0.014	0.89 (0.83, 0.97)
Model 2	1.00 (ref.)	1.04 (0.84, 1.29)	0.79 (0.62, 1.001)	0.92 (0.73, 1.17)	0.92 (0.72, 1.19)	0.32	0.95 (0.87, 1.03)
Cancer mortality							
Cases	454	530	478	490	400		
Model 1	1.00 (ref.)	0.91 (0.80, 1.03)	0.90 (0.79, 1.02)	0.82 (0.72, 0.93)	0.75 (0.66, 0.86)	<.0001	0.92 (0.88, 0.96)
Model 2	1.00 (ref.)	0.96 (0.85, 1.09)	0.98 (0.86, 1.12)	0.91 (0.80, 1.04)	0.87 (0.76, 1.01)	0.047	0.97 (0.93, 1.01)
Neurodegenerative mortality							
Cases	65	69	69	53	56		
Model 1	1.00 (ref.)	0.80 (0.57, 1.13)	0.88 (0.62, 1.24)	0.60 (0.41, 0.87)	0.73 (0.50, 1.05)	0.041	0.94 (0.83, 1.07)
Model 2	1.00 (ref.)	0.79 (0.57, 1.11)	0.86 (0.61, 1.22)	0.57 (0.39, 0.83)	0.67 (0.46, 0.99)	0.017	0.93 (0.81, 1.06)
Respiratory mortality							
Cases	51	60	44	50	29		
Model 1	1.00 (ref.)	0.90 (0.62, 1.31)	0.73 (0.49, 1.11)	0.76 (0.51, 1.13)	0.52 (0.33, 0.82)	0.0033	0.81 (0.71, 0.93)
Model 2	1.00 (ref.)	0.97 (0.66, 1.43)	0.83 (0.55, 1.25)	0.86 (0.56, 1.30)	0.61 (0.37, 0.998)	0.043	0.86 (0.75, 0.99)
Other mortality							
Cases	137	160	135	122	97		
Model 1	1.00 (ref.)	0.92 (0.73, 1.16)	0.87 (0.69, 1.11)	0.71 (0.56, 0.91)	0.65 (0.50, 0.85)	0.0002	0.87 (0.80, 0.95)
Model 2	1.00 (ref.)	0.98 (0.77, 1.23)	0.95 (0.74, 1.21)	0.78 (0.60, 1.001)	0.72 (0.55, 0.96)	0.0061	0.90 (0.82, 0.99)

Model 1 adjusted for age (in years, continuous) and sex (male/female);

Model 2 adjusted for covariates in model 1 plus ethnicity (white/not-white), education (lower secondary, upper secondary, vocational, college or university, or others), TDI (in quintiles), assessment centers (22 categories), smoking (current, former, or never), physical activity (0-599, 600-1199, ≥1200 MET-mins/week, or unknown), BMI (<25.0, 25.0–29.9, ≥30 kg/m^2, or unknown), total energy intake (KCAL, continuous), alcohol consumption (0, 0.1–5, 5.1–10, 10.1–15, 15.1–20, 20.1–30, >30 g/day), baseline dyslipidemia (yes/no), hypertension (yes/no), diabetes (yes/no), longevity-PRS (in tertiles), top 10 genetic primary components (continuous), and genotype measurement batches (continuous).
Abbreviations: BMI, body mass index; CI, confidence interval; hPDI, healthful plant-based diet; MET, metabolic equivalent of task; PRS, polygenic risk score; SD, standard deviation; TDI, Townsend Deprivation Index.

Table S22. Hazard ratio (95% CI) of cause-specific mortality associated with the dietary approaches to stop hypertension using a Fine-Gray model

	Quintile of DASH					*P* for trend	per SD increment
	Quintile 1	Quintile 2	Quintile 3	Quintile 4	Quintile 5		
Median score	16 (15, 18)	20 (19, 21)	22 (22, 23)	25 (24, 26)	29 (27, 30)		
No. of participants	22,185	22,939	16,949	21,701	19,875		
Person-years	234,222	241,870	179,063	229,151	210,162		
CVD mortality							
Cases	182	161	129	174	119		
Model 1	1.00 (ref.)	0.77 (0.63, 0.96)	0.84 (0.67, 1.05)	0.88 (0.71, 1.08)	0.68 (0.54, 0.86)	0.011	0.89 (0.83, 0.96)
Model 2	1.00 (ref.)	0.85 (0.68, 1.05)	0.95 (0.75, 1.19)	1.02 (0.82, 1.26)	0.81 (0.64, 1.04)	0.35	0.95 (0.88, 1.03)
Cancer mortality							
Cases	536	577	361	468	410		
Model 1	1.00 (ref.)	0.92 (0.82, 1.04)	0.76 (0.66, 0.86)	0.75 (0.66, 0.85)	0.71 (0.62, 0.81)	<.0001	0.87 (0.83, 0.91)
Model 2	1.00 (ref.)	1.002 (0.89, 1.13)	0.84 (0.74, 0.97)	0.85 (0.75, 0.97)	0.84 (0.73, 0.96)	0.0012	0.93 (0.89, 0.97)
Neurodegenerative mortality							
Cases	59	81	52	65	55		
Model 1	1.00 (ref.)	1.14 (0.81, 1.60)	0.96 (0.65, 1.40)	0.91 (0.63, 1.31)	0.84 (0.57, 1.24)	0.19	0.93 (0.82, 1.05)
Model 2	1.00 (ref.)	1.12 (0.80, 1.57)	0.93 (0.63, 1.37)	0.86 (0.60, 1.25)	0.78 (0.52, 1.16)	0.09	0.90 (0.79, 1.02)
Respiratory mortality							
Cases	69	49	40	39	37		
Model 1	1.00 (ref.)	0.59 (0.41, 0.85)	0.64 (0.43, 0.95)	0.48 (0.32, 0.71)	0.51 (0.34, 0.77)	0.0009	0.76 (0.65, 0.88)
Model 2	1.00 (ref.)	0.67 (0.46, 0.97)	0.76 (0.51, 1.13)	0.57 (0.38, 0.86)	0.64 (0.42, 0.99)	0.026	0.83 (0.72, 0.96)
Other mortality							
Cases	147	181	91	129	103		
Model 1	1.00 (ref.)	1.08 (0.87, 1.35)	0.73 (0.56, 0.95)	0.80 (0.63, 1.02)	0.71 (0.55, 0.92)	0.0011	0.88 (0.81, 0.96)
Model 2	1.00 (ref.)	1.21 (0.97, 1.51)	0.83 (0.64, 1.08)	0.93 (0.73, 1.20)	0.85 (0.65, 1.12)	0.082	0.94 (0.87, 1.03)

Model 1 adjusted for age (in years, continuous) and sex (male/female);

Model 2 adjusted for covariates in model 1 plus ethnicity (white/not-white), education (lower secondary, upper secondary, vocational, college or university, or others), TDI (in quintiles), assessment centers (22 categories), smoking (current, former, or never), physical activity (0-599, 600-1199, ≥1200 MET-mins/week, or unknown), BMI (<25.0, 25.0–29.9, ≥30 kg/m^2, or unknown), total energy intake (KCAL, continuous), alcohol consumption (0, 0.1–5, 5.1–10, 10.1–15, 15.1–20, 20.1–30, >30 g/day), baseline dyslipidemia (yes/no), hypertension (yes/no), diabetes (yes/no), longevity-PRS (in tertiles), top 10 genetic primary components (continuous), and genotype measurement batches (continuous).
Abbreviations: BMI, body mass index; CI, confidence interval; DASH, dietary approaches to stop hypertension; MET, metabolic equivalent of task; PRS, polygenic risk score; SD, standard deviation; TDI, Townsend Deprivation Index.

Table S23. Hazard ratio (95% CI) of cause-specific mortality associated with the diabetes risk reduction diet using a Fine-Gray model

	Quintile of DRRD					*P* for trend	per SD increment
	Quintile 1	Quintile 2	Quintile 3	Quintile 4	Quintile 5		
Median score	18 (17, 20)	23 (22, 24)	25 (25, 26)	28 (27, 29)	33 (32, 35)		
No. of participants	18,488	26,362	14,635	24,610	19,554		
Person-years	194,850	278,220	154,510	259,930	206,957		
CVD mortality							
Cases	161	184	119	166	135		
Model 1	1.00 (ref.)	0.76 (0.61, 0.93)	0.87 (0.69, 1.11)	0.73 (0.59, 0.91)	0.75 (0.60, 0.94)	0.017	0.90 (0.84, 0.97)
Model 2	1.00 (ref.)	0.81 (0.65, 0.998)	0.95 (0.75, 1.20)	0.81 (0.65, 1.01)	0.85 (0.67, 1.08)	0.19	0.94 (0.87, 1.02)
Cancer mortality							
Cases	453	623	331	557	388		
Model 1	1.00 (ref.)	0.91 (0.80, 1.02)	0.85 (0.74, 0.98)	0.84 (0.75, 0.96)	0.72 (0.63, 0.83)	<.0001	0.90 (0.86, 0.93)
Model 2	1.00 (ref.)	0.96 (0.85, 1.09)	0.91 (0.79, 1.05)	0.92 (0.81, 1.04)	0.81 (0.70, 0.93)	0.0021	0.93 (0.89, 0.97)
Neurodegenerative mortality							
Cases	60	74	53	63	62		
Model 1	1.00 (ref.)	0.80 (0.57, 1.12)	1.01 (0.69, 1.46)	0.71 (0.49, 1.01)	0.86 (0.60, 1.23)	0.34	0.98 (0.87, 1.10)
Model 2	1.00 (ref.)	0.78 (0.55, 1.10)	0.97 (0.67, 1.41)	0.67 (0.47, 0.97)	0.80 (0.55, 1.16)	0.18	0.96 (0.85, 1.08)
Respiratory mortality							
Cases	64	54	34	57	25		
Model 1	1.00 (ref.)	0.54 (0.38, 0.78)	0.61 (0.40, 0.92)	0.61 (0.42, 0.87)	0.33 (0.21, 0.53)	<.0001	0.72 (0.63, 0.83)
Model 2	1.00 (ref.)	0.60 (0.42, 0.87)	0.68 (0.45, 1.03)	0.71 (0.50, 1.01)	0.39 (0.25, 0.62)	0.0003	0.77 (0.67, 0.88)
Other mortality							
Cases	138	182	91	147	93		
Model 1	1.00 (ref.)	0.88 (0.70, 1.09)	0.78 (0.60, 1.02)	0.75 (0.59, 0.95)	0.60 (0.46, 0.78)	<.0001	0.86 (0.80, 0.94)
Model 2	1.00 (ref.)	0.95 (0.76, 1.19)	0.86 (0.66, 1.12)	0.86 (0.68, 1.09)	0.69 (0.52, 0.90)	0.0045	0.91 (0.84, 0.99)

Model 1 adjusted for age (in years, continuous) and sex (male/female);

Model 2 adjusted for covariates in model 1 plus ethnicity (white/not-white), education (lower secondary, upper secondary, vocational, college or university, or others), TDI (in quintiles), assessment centers (22 categories), smoking (current, former, or never), physical activity (0-599, 600-1199, ≥1200 MET-mins/week, or unknown), BMI (<25.0, 25.0–29.9, ≥30 kg/m^2, or unknown), total energy intake (KCAL, continuous), alcohol consumption (0, 0.1–5, 5.1–10, 10.1–15, 15.1–20, 20.1–30, >30 g/day), baseline dyslipidemia (yes/no), hypertension (yes/no), diabetes (yes/no), longevity-PRS (in tertiles), top 10 genetic primary components (continuous), and genotype measurement batches (continuous).

Abbreviations: BMI, body mass index; CI, confidence interval; DRRD, diabetes risk reduction diet; MET, metabolic equivalent of task; PRS, polygenic risk score; SD, standard deviation; TDI, Townsend Deprivation Index.

Table S24. The components and scoring criteria of AHEI

Component	Criteria for minimum score (0 point)	Criteria for maximum score (10 points)
Vegetables (excluding potatoes), servings/d	0	≥5
Fruits, servings/d	0	≥4
Whole grains, servings/d	0	Women ≥5<br>Men ≥6
Nuts and legumes, servings/d	0	≥1
Long-chain (n-3) fats, mg/d	0	≥250
Polyunsaturated fatty acid (without EPA and DHA), % of energy	≤2	≥10
Sugar-sweetened beverages and fruit juice, servings/d	≥1	0
Red/processed meat, servings/d	≥1.5	0
Trans fatty acids, % of energy	≥4	≤0.5
Sodium, mg/day	Highest decile	Lowest decile
Alcohol, drinks/day	Women ≥2.5<br>Men≥3.5	Women 0-1.5<br>Men 0-2.0

Abbreviations: AHEI, Alternate healthy eating index; MUFA, monounsaturated fatty acid; SFA, saturated fatty acid.

Table S25. The components and scoring criteria of AMED*

Component	Criteria for minimum score (1 point)	Criteria for maximum score (5 points)
Vegetables (excluding potatoes), servings/day	Lowest quintile	Highest quintile
Fruits, servings/day	Lowest quintile	Highest quintile
Whole grains, servings/day	Lowest quintile	Highest quintile
Nuts, servings/day	Lowest quintile	Highest quintile
Legumes, servings/day	Lowest quintile	Highest quintile
MUFA:SFA ratio	Lowest quintile	Highest quintile
Dairy products, servings/d	Lowest quintile	Highest quintile
Fish, servings/d	Lowest quintile	Highest quintile
Red/processed meat, servings/d	Highest quintile	Lowest quintile
Alcohol, grams/day	Women ≥35<br>Men≥50	Women 5-15<br>Men 10-30

* All used sex-specific quintiles

Abbreviations: AMED, Alternate Mediterranean diet; MUFA, monounsaturated fatty acid; SFA, saturated fatty acid.

Table S26. The components and scoring criteria of hPDI

Food groups	Food items	Criteria for minimum score (1 point)	Criteria for maximum score (5 points)
**Healthy plant-based food groups, servings/d**			
Whole grains	Porridge, muesli, plain cereal, bran cereal, whole-wheat cereal, oatcakes, wholemeal bread (flour type: wholemeal), whole meal pasta, brown rice, couscous, other cooked grains (such as bulgur).	Lowest quintile	Highest quintile
Fruits	Stewed fruit, prune, dried fruit, mixed fruit, apple, banana, berries, cherries, grapefruit, grapes, mango, melon, orange, orange-like small fruits, peach/nectarine, pear, pineapple, plum, other fruits, olives	Lowest quintile	Highest quintile
Vegetables	Mixed vegetables, vegetable pieces, coleslaw, side salad, avocado, beetroot, broccoli, butternut squash, cabbage/kale, carrots, cauliflower, celery, courgette, cucumber, garlic, leeks, lettuce, mushrooms, onion, parsnip, sweet peppers, spinach, sprouts, sweetcorn, fresh tomatoes, cooked or tinned tomatoes, turnip/swede, watercress, other vegetables	Lowest quintile	Highest quintile
Nuts	Salted peanuts, unsalted peanuts, salted nuts, unsalted nuts, seeds	Lowest quintile	Highest quintile
Legumes	Vegetarian sausages/burgers, tofu, Quorn, other vegetarian alternative, baked beans, pulse, broad beans, green beans, peas	Lowest quintile	Highest quintile
Tea and coffee	Instant coffee, filtered coffee, cappuccino, latte, espresso, other coffee drinks, standard tea, rooibos tea, green tea, herbal tea, other tea, low-calorie hot chocolate, hot chocolate	Lowest quintile	Highest quintile
**Unhealthy plant-based food groups, servings/d**			
Refined grains	Oat crunch, sweetened cereal, other cereal, white bread (flour types: white, mix, and others), naan bread, garlic bread, crispbread, other bread, white pasta, white rice, snack pot, couscous, pancake, scotch pancake, croissant, scone, savoury biscuits, cheesy biscuits, sweet potato, other savoury snack	Highest quintile	Lowest quintile
Potatoes	Fried potatoes, boiled/baked potatoes, mashed potatoes, crisps (e.g., potato chips)	Highest quintile	Lowest quintile
Sugary drinks	Low-calorie or diet drinks (e.g. fizzy, squash), carbonated (fizzy) drinks, squash or cordial	Highest quintile	Lowest quintile
Fruit juices	Orange juice, grapefruit juice, other fruit/vegetable juice, fruit smoothie	Highest quintile	Lowest quintile

Sweets and desserts	Double crust pie, single crust pie/flan, crumble topping, Yorkshire pudding, Danish pastry, fruitcake, cake, doughnuts, sponge pudding, other dessert, chocolate bar, white chocolate, milk chocolate, dark chocolate, chocolate-covered raisin, chocolate sweet, diet sweets, chocolate-covered biscuits, chocolate biscuits, sweet biscuits, other sweets	Highest quintile	Lowest quintile
**Animal-based food groups, servings/d**			
Animal fat	Butter on bread/crackers (spreadable, low fat, normal fat, or unknown type), dairy spread on bread/crackers (very low fat, low fat, normal fat, unknown type)	Highest quintile	Lowest quintile
Dairy	Milk, dairy smoothie, flavored milk, yogurt, ice cream, cheesecake, milk-based pudding, other milk-based pudding, low-fat hard cheese, hard cheese, soft cheese, blue cheese, low-fat cheese spread, cheese spread, cottage cheese, feta cheese, mozzarella cheese, goat's cheese, other cheese	Highest quintile	Lowest quintile
Eggs	Whole eggs, omelettes or scrambled eggs, eggs in sandwiches, scotch eggs, other egg dishes	Highest quintile	Lowest quintile
Fish or seafood	Tinned tuna, oily fish, breaded fish, battered fish, white fish, prawns, lobster/crab, shellfish, other fish	Highest quintile	Lowest quintile
Meat	Sausage, beef, pork, lamb, crumbed or deep-fried poultry, poultry, bacon, ham, liver, other meat	Highest quintile	Lowest quintile
Miscellaneous animal-based foods	Pizza, Indian snacks	Highest quintile	Lowest quintile

Abbreviations: hPDI, healthful plant-based diet index.

Table S27. The components and scoring criteria of DASH

Component	Criteria for minimum score (1 point)	Criteria for maximum score (5 points)
Vegetables (excluding potatoes), servings/day	Lowest quintile	Highest quintile
Fruits, servings/day	Lowest quintile	Highest quintile
Whole grains, servings/day	Lowest quintile	Highest quintile
Nuts and legumes, servings/day	Lowest quintile	Highest quintile
Low-fat dairy products, servings/d	Lowest quintile	Highest quintile
Red/processed meat, servings/d	Highest quintile	Lowest quintile
Sugar-sweetened beverages and fruit juice, servings/d	Highest quintile	Lowest quintile
Sodium, mg/d	Highest quintile	Lowest quintile

Abbreviations: DASH, dietary approaches to stop hypertension.

Table S28. The components and scoring criteria of DRRD

Component	Criteria for minimum score (1 point)	Criteria for maximum score (5 points)
Fiber, grams/day	Lowest quintile	Highest quintile
Fruits, servings/day	Lowest quintile	Highest quintile
Coffee, servings/day	Lowest quintile	Highest quintile
Nuts, servings/day	Lowest quintile	Highest quintile
MUFA: SFA ratio	Lowest quintile	Highest quintile
Red/processed meat, servings/d	Highest quintile	Lowest quintile
Trans fatty acids, % of energy	Highest quintile	Lowest quintile
Sugar-sweetened beverages and fruit juice, servings/d	Highest quintile	Lowest quintile
Glycemic index	Highest quintile	Lowest quintile

Abbreviations: DRRD, diabetes risk reduction diet; MUFA, monounsaturated fatty acid; SFA, saturated fatty acid.

Table S29. Disease definitions in the UK Biobank study

	**ICD-9**	**ICD-10**	**OPCS-4**	**Self-reported (Field ID)**	**Examination**
**Dyslipidemia**	272	E78		6153, 6177, 20002, 20003	30760, 30870
**Hypertension**	401, 402, 403, 404, 405	I10, I11, I12, I13, I15, O10, O11		6150, 2966, 6153, 6177, 20002	4079, 4080, 93, 94
**Diabetes Mellitus**	250	E10-E14		2443, 2976, 6153, 6177, 20002	30740, 30750
**Cardiovascular disease***	410, 411, 412, 413, 414, 428, 4280, 4281, 4289, 430, 431, 434, 4340, 4341, 4349, 436	I20-25, I50, I500, I501, I509, I60, I61, I63, I64	A052-A054, K40-K46, K49, K50, K75, L351, L353, L343	3894, 6150, 6153, 6177, 20004, 20002	6150, 2966, 6153, 6177, 20002
**Cancer**		C00-C97, D00-D48, O00-O08		2453, 20001	

*Cardiovascular disease is composed of coronary heart disease, stroke, and heart failure.
Abbreviations: ICD, International Classification of Diseases; OPCS-4, Office of Population Censuses and Surveys Classification of Interventions and Procedures, version 4.

Table S30. Characteristics of longevity-associated SNPs in the UK Biobank

At or near gene	rsid	Chr	Position	A1	frequency of A1	Years of life gain	SE	*P* value
MAGI3	rs1230666	1	114173410	G	0.85	0.3224	0.06	6.4×10^{-9}
CELSR2/PSRC1	rs4970836	1	109821797	G	0.23	0.2234	0.0463	1.6E-09
GBX2/ASB18	rs10211471	2	237081854	C	0.80	0.2401	0.0493	2.3E-08
KCNK3	rs1275922	2	26932887	G	0.74	0.2579	0.04	6.0×10^{-9}
TMEM18	rs6744653	2	628524	A	0.17	0.2772	0.0511	7.0E-10
HTT	rs61348208	4	3089564	T	0.39	0.2299	0.04	5.8×10^{-9}
HLA-DQA1	rs34967069	6	32591248	T	0.07	0.5613	0.10	4.3×10^{-9}
IGF2R	rs111333005	6	160487196	G	0.98	0.8665	0.1577	6.6E-09
LPA	rs10455872	6	161010118	A	0.92	0.7639	0.07	8.5×10^{-25}
POM121C	rs113160991	7	75094329	G	0.78	0.2541	0.0495	7.5E-09
ZC3HC1	rs56179563	7	129685597	A	0.39	0.2107	0.0406	5.6E-09
ABO	rs2519093	9	136141870	C	0.81	0.2244	0.0497	1.9E-08
CDKN2B-AS1	rs1556516	9	22100176	G	0.5	0.2510	0.04	7.5×10^{-11}
ATXN2/BRAP	rs11065979	12	112059557	C	0.56	0.2798	0.04	1.0×10^{-12}
CHRNA3/5	rs8042849	15	78817929	T	0.65	0.4368	0.04	1.6×10^{-26}
FURIN/FES	rs6224	15	91423543	G	0.52	0.2507	0.04	1.3×10^{-10}
HP	rs12924886	16	72075593	A	0.8	0.2798	0.05	1.4×10^{-8}
LDLR	rs142158911	19	11190534	A	0.12	0.3550	0.06	8.1×10^{-9}
APOE	rs429358	19	45411941	T	0.85	1.0561	0.05	3.1×10^{-83}

Abbreviations: Chr, chromosome; A1, risk allele; SE, standard error; SNPs, single-nucleotide polymorphisms.

REFERENCES

1. National life tables—Life expectancy in the UK: 2018 to 2020; www.ons.gov.uk/peoplepopulationandcommunity/birthsdeathsandmarriages/lifeexpectancies/bulletins/nationallifetablesunitedkingdom/2018to2020.

2. GBD 2017 Diet Collaborators, Health effects of dietary risks in 195 countries, 1990-2017: A systematic analysis for the Global Burden of Disease Study 2017. *Lancet* **393**, 1958–1972 (2019).

3. F. B. Hu, Diet strategies for promoting healthy aging and longevity: An epidemiological perspective. *J. Intern. Med.* **295**, 508–531 (2024).

4. F. B. Hu, Dietary pattern analysis: A new direction in nutritional epidemiology. *Curr. Opin. Lipidol.* **13**, 3–9 (2002).

5. Z. Shan, F. Wang, Y. Li, M. Y. Baden, S. N. Bhupathiraju, D. D. Wang, Q. Sun, K. M. Rexrode, E. B. Rimm, L. Qi, F. K. Tabung, E. L. Giovannucci, W. C. Willett, J. E. Manson, Q. Qi, F. B. Hu, Healthy eating patterns and risk of total and cause-specific mortality. *JAMA Intern. Med.* **183**, 142–153 (2023).

6. P. Wang, M. Song, A. H. Eliassen, M. Wang, T. T. Fung, S. K. Clinton, E. B. Rimm, F. B. Hu, W. C. Willett, F. K. Tabung, E. L. Giovannucci, Optimal dietary patterns for prevention of chronic disease. *Nat. Med.* **29**, 719–728 (2023).

7. X. Shang, J. Liu, Z. Zhu, X. Zhang, Y. Huang, S. Liu, W. Wang, X. Zhang, S. Tang, Y. Hu, H. Yu, Z. Ge, M. He, Healthy dietary patterns and the risk of individual chronic diseases in community-dwelling adults. *Nat. Commun.* **14**, 6704 (2023).

8. Y.-W. Cai, J.-W. Gao, M.-X. Wu, Y.-X. Xie, S. You, G.-H. Liao, Z.-T. Chen, P.-M. Liu, J.-F. Wang, Y.-X. Chen, H.-F. Zhang, Adherence to EAT-Lancet diet, biological aging, and life expectancy in the UK Biobank: A cohort study. *Am. J. Clin. Nutr.* **122**, 29–38 (2025).

9. K. Zhu, R. Li, P. Yao, H. Yu, A. Pan, J. E. Manson, E. B. Rimm, W. C. Willett, G. Liu, Proteomic signatures of healthy dietary patterns are associated with lower risks of major chronic diseases and mortality. *Nat. Food* **6**, 47–57 (2025).

10. Q. Sun, D. Yu, J. Fan, C. Yu, Y. Guo, P. Pei, L. Yang, Y. Chen, H. Du, X. Yang, S. Sansome, Y. Wang, W. Zhao, J. Chen, Z. Chen, L. Zhao, J. Lv, L. Li, China Kadoorie Biobank Collaborative Group, Healthy lifestyle and life expectancy at age 30 years in the Chinese population: An observational study. *Lancet Public Health* **7**, e994–e1004 (2022).

11. J. Deelen, D. S. Evans, D. E. Arking, N. Tesi, M. Nygaard, X. Liu, M. K. Wojczynski, M. L. Biggs, A. van der Spek, G. Atzmon, E. B. Ware, C. Sarnowski, A. V. Smith, I. Seppälä, H. J. Cordell, J. Dose, N. Amin, A. M. Arnold, K. L. Ayers, N. Barzilai, E. J. Becker, M. Beekman, H. Blanché, K. Christensen, L. Christiansen, J. C. Collerton, S. Cubaynes, S. R. Cummings, K. Davies, B. Debrabant, J. F. Deleuze, R. Duncan, J. D. Faul, C. Franceschi, P. Galan, V. Gudnason, T. B. Harris, M. Huisman, M. A. Hurme, C. Jagger, I. Jansen, M. Jylhä, M. Kähönen, D. Karasik, S. L. R. Kardia, A. Kingston, T. B. L. Kirkwood, L. J. Launer, T. Lehtimäki, W. Lieb, L. P. Lyytikäinen, C. Martin-Ruiz, J. Min, A. Nebel, A. B. Newman, C. Nie, E. A. Nohr, E. S. Orwoll, T. T. Perls, M. A. Province, B. M. Psaty, O. T. Raitakari, M. J. T. Reinders, J. M. Robine, J. I. Rotter, P. Sebastiani, J. Smith, T. I. A. Sørensen, K. D. Taylor, A. G. Uitterlinden, W. van der Flier, S. J. van der Lee, C. M. van Duijn, D. van Heemst, J. W. Vaupel, D. Weir, K. Ye, Y. Zeng, W. Zheng, H. Holstege, D. P. Kiel, K. L. Lunetta, P. E. Slagboom, J. M. Murabito, A meta-analysis of genome-wide association studies identifies multiple longevity genes. *Nat. Commun.* **10**, 3669 (2019).

12. P. R. Timmers, N. Mounier, K. Lall, K. Fischer, Z. Ning, X. Feng, A. D. Bretherick, D. W. Clark, X. Shen, T. Esko, Z. Kutalik, J. F. Wilson, P. K. Joshi, Genomics of 1 million parent lifespans implicates novel pathways and common diseases and distinguishes survival chances. *eLife* **8**, e39856 (2019).

13. S. F. W. Meddens, R. de Vlaming, P. Bowers, C. A. P. Burik, R. K. Linnér, C. Lee, A. Okbay, P. Turley, C. A. Rietveld, M. A. Fontana, M. Ghanbari, F. Imamura, G. McMahon, P. J. van der Most, T. Voortman, K. H. Wade, E. L. Anderson, K. V. E. Braun, P. M. Emmett, T. Esko, J. R.

Gonzalez, J. C. Kiefte-de Jong, C. Langenberg, J. Luan, T. Muka, S. Ring, F. Rivadeneira, H. Snieder, F. J. A. van Rooij, B. H. R. Wolffenbuttel, G. D. Smith, O. H. Franco, N. G. Forouhi, M. A. Ikram, A. G. Uitterlinden, J. V. van Vliet-Ostaptchouk, N. J. Wareham, D. Cesarini, K. P. Harden, J. J. Lee, D. J. Benjamin, C. C. Chow, P. D. Koellinger, Genomic analysis of diet composition finds novel loci and associations with health and lifestyle. *Mol. Psychiatry* **26**, 2056–2069 (2021).

14. F. Y. Ideraabdullah, S. H. Zeisel, Dietary modulation of the epigenome. *Physiol. Rev.* **98**, 667–695 (2018).

15. A. V. Khera, C. A. Emdin, I. Drake, P. Natarajan, A. G. Bick, N. R. Cook, D. I. Chasman, U. Baber, R. Mehran, D. J. Rader, V. Fuster, E. Boerwinkle, O. Melander, M. Orho-Melander, P. M. Ridker, S. Kathiresan, Genetic risk, adherence to a healthy lifestyle, and coronary disease. *N. Engl. J. Med.* **375**, 2349–2358 (2016).

16. V. L. Chen, X. Du, A. Oliveri, Y. Chen, A. Kuppa, B. D. Halligan, M. A. Province, E. K. Speliotes, Genetic risk accentuates dietary effects on hepatic steatosis, inflammation and fibrosis in a population-based cohort. *J. Hepatol.* **81**, 379–388 (2024).

17. B. E. Harmon, C. J. Boushey, Y. B. Shvetsov, R. Ettienne, J. Reedy, L. R. Wilkens, L. Le Marchand, B. E. Henderson, L. N. Kolonel, Associations of key diet-quality indexes with mortality in the Multiethnic Cohort: The Dietary Patterns Methods Project. *Am. J. Clin. Nutr.* **101**, 587–597 (2015).

18. M. Sotos-Prieto, S. N. Bhupathiraju, J. Mattei, T. T. Fung, Y. Li, A. Pan, W. C. Willett, E. B. Rimm, F. B. Hu, Association of changes in diet quality with total and cause-specific mortality. *N. Engl. J. Med.* **377**, 143–153 (2017).

19. E. A. Hu, L. M. Steffen, J. Coresh, L. J. Appel, C. M. Rebholz, Adherence to the Healthy Eating Index-2015 and other dietary patterns may reduce risk of cardiovascular disease, cardiovascular mortality, and all-cause mortality. *J. Nutr.* **150**, 312–321 (2020).

20. C. R. Wang, T. Y. Hu, F. B. Hao, N. Chen, Y. Peng, J. J. Wu, P. F. Yang, G. C. Zhong, Type 2 diabetes–prevention diet and all-cause and cause-specific mortality: A prospective study. *Am. J. Epidemiol.* **191**, 472–486 (2022).

21. M. B. Lee, C. M. Hill, A. Bitto, M. Kaeberlein, Antiaging diets: Separating fact from fiction. *Science* **374**, eabe7365 (2021).

22. Y. Li, A. Pan, D. D. Wang, X. Liu, K. Dhana, O. H. Franco, S. Kaptoge, E. Di Angelantonio, M. Stampfer, W. C. Willett, F. B. Hu, Impact of healthy lifestyle factors on life expectancies in the US population. *Circulation* **138**, 345–355 (2018).

23. H. Lagström, S. Stenholm, T. Akbaraly, J. Pentti, J. Vahtera, M. Kivimäki, J. Head, Diet quality as a predictor of cardiometabolic disease-free life expectancy: The Whitehall II cohort study. *Am. J. Clin. Nutr.* **111**, 787–794 (2020).

24. D. Melzer, L. C. Pilling, L. Ferrucci, The genetics of human ageing. *Nat. Rev. Genet.* **21**, 88–101 (2020).

25. Y. Zeng, H. Chen, T. Ni, R. Ruan, C. Nie, X. Liu, L. Feng, F. Zhang, J. Lu, J. Li, Y. Li, W. Tao, S. G. Gregory, W. Gottschalk, M. W. Lutz, K. C. Land, A. Yashin, Q. Tan, Z. Yang, L. Bolund, Q. Ming, H. Yang, J. Min, D. C. Willcox, B. J. Willcox, J. Gu, E. Hauser, X. L. Tian, J. W. Vaupel, Interaction between the FOXO1A-209 genotype and tea drinking is significantly associated with reduced mortality at advanced ages. *Rejuvenation Res.* **19**, 195–203 (2016).

26. X. Jin, S. Xiong, C. Yuan, E. Gong, X. Zhang, Y. Yao, Y. Leng, Z. Niu, Y. Zeng, L. L. Yan, Apolipoprotein E genotype, meat, fish, and egg intake in relation to mortality among older adults: A longitudinal analysis in China. *Front. Med.* **8**, 697389 (2021).

27. N. R. Wray, T. Lin, J. Austin, J. J. McGrath, I. B. Hickie, G. K. Murray, P. M. Visscher, From basic science to clinical application of polygenic risk scores: A primer. *JAMA Psychiatry* **78**, 101–109 (2021).

28. B. H. Parmenter, A. S. Thompson, N. P. Bondonno, A. Jennings, K. Murray, A. Perez-Cornago, J. M. Hodgson, A. Tresserra-Rimbau, T. Kühn, A. Cassidy, High diversity of dietary flavonoid

intake is associated with a lower risk of all-cause mortality and major chronic diseases. *Nat. Food* **6**, 668–680 (2025).

29. M. He, R. M. van Dam, E. Rimm, F. B. Hu, L. Qi, Whole-grain, cereal fiber, bran, and germ intake and the risks of all-cause and cardiovascular disease-specific mortality among women with type 2 diabetes mellitus. *Circulation* **121**, 2162–2168 (2010).

30. H. C. Wastyk, G. K. Fragiadakis, D. Perelman, D. Dahan, B. D. Merrill, F. B. Yu, M. Topf, C. G. Gonzalez, W. Van Treuren, S. Han, J. L. Robinson, J. E. Elias, E. D. Sonnenburg, C. D. Gardner, J. L. Sonnenburg, Gut-microbiota-targeted diets modulate human immune status. *Cell* **184**, 4137–4153.e14 (2021).

31. Y.-L. Xiao, Y. Gong, Y.-J. Qi, Z.-M. Shao, Y.-Z. Jiang, Effects of dietary intervention on human diseases: Molecular mechanisms and therapeutic potential. *Signal Transduct. Target. Ther.* **9**, 59 (2024).

32. J. A. Ambrose, R. S. Barua, The pathophysiology of cigarette smoking and cardiovascular disease: An update. *J. Am. Coll. Cardiol.* **43**, 1731–1737 (2004).

33. M. B. Schulze, A. M. Minihane, R. N. M. Saleh, U. Risérus, Intake and metabolism of omega-3 and omega-6 polyunsaturated fatty acids: Nutritional implications for cardiometabolic diseases. *Lancet Diabetes Endocrinol.* **8**, 915–930 (2020).

34. Y. Zhang, K. Luo, B. A. Peters, Y. Mossavar-Rahmani, J. Y. Moon, Y. Wang, M. L. Daviglus, L. Van Horn, A. C. McClain, C. Cordero, J. S. Floyd, B. Yu, R. W. Walker, R. D. Burk, R. C. Kaplan, Q. Qi, Sugar-sweetened beverage intake, gut microbiota, circulating metabolites, and diabetes risk in Hispanic Community Health Study/Study of Latinos. *Cell Metab.* **37**, 578–591.e4 (2025).

35. B. Geidl-Flueck, M. Hochuli, Á. Németh, A. Eberl, N. Derron, H. C. Köfeler, L. Tappy, K. Berneis, G. A. Spinas, P. A. Gerber, Fructose- and sucrose- but not glucose-sweetened beverages promote hepatic de novo lipogenesis: A randomized controlled trial. *J. Hepatol.* **75**, 46–54 (2021).

36. D. J. A. Jenkins, W. C. Willett, S. Yusuf, F. B. Hu, A. J. Glenn, S. Liu, A. Mente, V. Miller, S. I. Bangdiwala, H. C. Gerstein, S. Sieri, P. Ferrari, A. V. Patel, M. L. McCullough, L. Le Marchand, N. D. Freedman, E. Loftfield, R. Sinha, X. O. Shu, M. Touvier, N. Sawada, S. Tsugane, P. A. van den Brandt, K. Shuval, T. A. Khan, M. Paquette, S. Sahye-Pudaruth, D. Patel, T. F. Y. Siu, K. Srichaikul, C. W. C. Kendall, J. L. Sievenpiper, Clinical Nutrition & Risk Factor Modification Centre Collaborators, Association of glycaemic index and glycaemic load with type 2 diabetes, cardiovascular disease, cancer, and all-cause mortality: A meta-analysis of mega cohorts of more than 100 000 participants. *Lancet Diabetes Endocrinol.* **12**, 107–118 (2024).

37. D. J. A. Jenkins, M. Dehghan, A. Mente, S. I. Bangdiwala, S. Rangarajan, K. Srichaikul, V. Mohan, A. Avezum, R. Díaz, A. Rosengren, F. Lanas, P. Lopez-Jaramillo, W. Li, A. Oguz, R. Khatib, P. Poirier, N. Mohammadifard, A. Pepe, K. F. Alhabib, J. Chifamba, A. H. Yusufali, R. Iqbal, K. Yeates, K. Yusoff, N. Ismail, K. Teo, S. Swaminathan, X. Liu, K. Zatońska, R. Yusuf, S. Yusuf, PURE Study Investigators, Glycemic index, glycemic load, and cardiovascular disease and mortality. *N. Engl. J. Med.* **384**, 1312–1322 (2021).

38. S. Penumutchu, B. J. Korry, K. Hewlett, P. Belenky, Fiber supplementation protects from antibiotic-induced gut microbiome dysbiosis by modulating gut redox potential. *Nat. Commun.* **14**, 5161 (2023).

39. C. Sudlow, J. Gallacher, N. Allen, V. Beral, P. Burton, J. Danesh, P. Downey, P. Elliott, J. Green, M. Landray, B. Liu, P. Matthews, G. Ong, J. Pell, A. Silman, A. Young, T. Sprosen, T. Peakman, R. Collins, UK Biobank: An open access resource for identifying the causes of a wide range of complex diseases of middle and old age. *PLoS Med.* **12**, e1001779 (2015).

40. B. Liu, H. Young, F. L. Crowe, V. S. Benson, E. A. Spencer, T. J. Key, P. N. Appleby, V. Beral, Development and evaluation of the Oxford WebQ, a low-cost, web-based method for assessment of previous 24 h dietary intakes in large-scale prospective studies. *Public Health Nutr.* **14**, 1998–2005 (2011).

41. D. C. Greenwood, L. J. Hardie, G. S. Frost, N. A. Alwan, K. E. Bradbury, M. Carter, P. Elliott, C. E. L. Evans, H. E. Ford, N. Hancock, T. J. Key, B. Liu, M. A. Morris, U. Z. Mulla, K.

Petropoulou, G. D. M. Potter, E. Riboli, H. Young, P. A. Wark, J. E. Cade, Validation of the Oxford WebQ Online 24-hour dietary questionnaire using biomarkers. *Am. J. Epidemiol.* **188**, 1858–1867 (2019).

42. A. Perez-Cornago, Z. Pollard, H. Young, M. van Uden, C. Andrews, C. Piernas, T. J. Key, A. Mulligan, M. Lentjes, Description of the updated nutrition calculation of the Oxford WebQ questionnaire and comparison with the previous version among 207,144 participants in UK Biobank. *Eur. J. Nutr.* **60**, 4019–4030 (2021).

43. S. E. Chiuve, T. T. Fung, E. B. Rimm, F. B. Hu, M. L. McCullough, M. Wang, M. J. Stampfer, W. C. Willett, Alternative dietary indices both strongly predict risk of chronic disease. *J. Nutr.* **142**, 1009–1018 (2012).

44. A. Trichopoulou, T. Costacou, C. Bamia, D. Trichopoulos, Adherence to a Mediterranean diet and survival in a Greek population. *N. Engl. J. Med.* **348**, 2599–2608 (2003).

45. A. Satija, S. N. Bhupathiraju, D. Spiegelman, S. E. Chiuve, J. E. Manson, W. Willett, K. M. Rexrode, E. B. Rimm, F. B. Hu, Healthful and unhealthful plant-based diets and the risk of coronary heart disease in U.S. adults. *J. Am. Coll. Cardiol.* **70**, 411–422 (2017).

46. Y. Lv, S. Rong, Y. Deng, W. Bao, Y. Xia, L. Chen, Plant-based diets, genetic predisposition and risk of non-alcoholic fatty liver disease. *BMC Med.* **21**, 351 (2023).

47. T. T. Fung, S. E. Chiuve, M. L. McCullough, K. M. Rexrode, G. Logroscino, F. B. Hu, Adherence to a DASH-style diet and risk of coronary heart disease and stroke in women. *Arch. Intern. Med.* **168**, 713–720 (2008).

48. J. J. Rhee, J. Mattei, M. D. Hughes, F. B. Hu, W. C. Willett, Dietary diabetes risk reduction score, race and ethnicity, and risk of type 2 diabetes in women. *Diabetes Care* **38**, 596–603 (2015).

49. C. Bycroft, C. Freeman, D. Petkova, G. Band, L. T. Elliott, K. Sharp, A. Motyer, D. Vukcevic, O. Delaneau, J. O'Connell, A. Cortes, S. Welsh, A. Young, M. Effingham, G. McVean, S. Leslie,

N. Allen, P. Donnelly, J. Marchini, The UK Biobank resource with deep phenotyping and genomic data. *Nature* **562**, 203–209 (2018).

50. Single-year life tables, UK: 1980 to 2020; www.ons.gov.uk/peoplepopulationandcommunity/birthsdeathsandmarriages/lifeexpectancies/datasets/singleyearlifetablesuk1980to2018.

51. R. Li, L. Chambless, Test for additive interaction in proportional hazards models. *Ann. Epidemiol.* **17**, 227–236 (2007).

52. D. B. Ibsen, A. S. D. Laursen, A. M. L. Würtz, C. C. Dahm, E. B. Rimm, E. T. Parner, K. Overvad, M. U. Jakobsen, Food substitution models for nutritional epidemiology. *Am. J. Clin. Nutr.* **113**, 294–303 (2021).

53. GBD 2020 Alcohol Collaborators, Population-level risks of alcohol consumption by amount, geography, age, sex, and year: A systematic analysis for the Global Burden of Disease Study 2020. *Lancet* **400**, 185–235 (2022).

54. H. Ma, Q. Xue, X. Wang, X. Li, O. H. Franco, Y. Li, Y. Heianza, J. E. Manson, L. Qi, Adding salt to foods and hazard of premature mortality. *Eur. Heart J.* **43**, 2878–2888 (2022).

55. C. L. Chiang, *Life Table and Mortality Analysis* (World Health Organization, 1979).

56. S. Woloshin, L. M. Schwartz, H. G. Welch, The risk of death by age, sex, and smoking status in the United States: Putting health risks in context. *J. Natl. Cancer Inst.* **100**, 845–853 (2008).
